# Divergent signaling requirements of dSARM in injury-induced degeneration and developmental glial phagocytosis

**DOI:** 10.1371/journal.pgen.1010257

**Published:** 2022-06-23

**Authors:** Kelsey A. Herrmann, Yizhou Liu, Arnau Llobet-Rosell, Colleen N. McLaughlin, Lukas J. Neukomm, Jaeda C. Coutinho-Budd, Heather T. Broihier

**Affiliations:** 1 Department of Neurosciences, Case Western Reserve University School of Medicine, Cleveland, Ohio, United States of America; 2 Department of Fundamental Neurosciences, University of Lausanne, Lausanne, Switzerland; 3 Department of Neuroscience, University of Virginia, Charlottesville, Virginia, United States of America; National University of Singapore, SINGAPORE

## Abstract

Elucidating signal transduction mechanisms of innate immune pathways is essential to defining how they elicit distinct cellular responses. Toll-like receptors (TLR) signal through their cytoplasmic TIR domains which bind other TIR domain-containing adaptors. dSARM/SARM1 is one such TIR domain adaptor best known for its role as the central axon degeneration trigger after injury. In degeneration, SARM1’s domains have been assigned unique functions: the ARM domain is auto-inhibitory, SAM-SAM domain interactions mediate multimerization, and the TIR domain has intrinsic NAD^+^ hydrolase activity that precipitates axonal demise. Whether and how these distinct functions contribute to TLR signaling is unknown. Here we show divergent signaling requirements for dSARM in injury-induced axon degeneration and TLR-mediated developmental glial phagocytosis through analysis of new knock-in domain and point mutations. We demonstrate intragenic complementation between reciprocal pairs of domain mutants during development, providing evidence for separability of dSARM functional domains in TLR signaling. Surprisingly, dSARM’s NAD^+^ hydrolase activity is strictly required for both degenerative and developmental signaling, demonstrating that TLR signal transduction requires dSARM’s enzymatic activity. In contrast, while SAM domain-mediated dSARM multimerization is important for axon degeneration, it is dispensable for TLR signaling. Finally, dSARM functions in a linear genetic pathway with the MAP3K Ask1 during development but not in degenerating axons. Thus, we propose that dSARM exists in distinct signaling states in developmental and pathological contexts.

## Author summary

Following injury, severed axons are actively destroyed by a protein called SARM1, or dSARM in Drosophila. It was recently shown that dSARM/SARM1 accomplishes this feat by degrading the key coenzyme NAD^+^, leading to energetic collapse in the axon. This was surprising since dSARM/SARM1 is a TIR-domain containing protein, and as such, was thought to act exclusively as an adaptor in Toll receptor-mediated pathways. We recently uncovered a developmental role for dSARM in glia where it promotes engulfment of dying neurons. To understand the relationship between this function for dSARM and its better-known function in axon degeneration, we set out to (1) compare the enzymatic requirement of dSARM in both settings, and (2) determine if signaling mechanisms are the same. To address these questions, we used CRISPR to generate new dSARM knock-in alleles. We find that the NAD^+^ hydrolase activity of dSARM is absolutely required for both its axon degeneration function and its signaling function. Pointing to differences in dSARM-mediated pathways in the two contexts, we provide evidence that Ask1 kinase is required for dSARM signaling in glia but not for axon degeneration. Thus, we propose that the NAD^+^ hydrolase activity of dSARM leads to Ask1 activation and subsequent signal transduction.

## Introduction

Brain homeostasis is maintained by cell-intrinsic and cell-extrinsic surveillance mechanisms. During normal development, a commonly cited estimate is that 50% of neurons die, and injury can precipitate the death of even more. The prevalence of neuron and neurite death during development highlights the importance of defining underlying molecular mechanisms as well as those active in phagocytic glia that engulf and dispose of neuronal corpses.

Wallerian degeneration is a specific type of axon degeneration in which the axon distal to an axotomy degenerates [1]. A spontaneous mouse mutant, Wallerian Degeneration Slow (Wld^S^), exhibits markedly delayed axon degeneration [2,3]. This phenotype argues that axon degeneration is an active process and not passive wasting of the injured nerve. The Wld^S^ mutation is a tandem triplication of the NAD^+^ synthetic enzyme Nicotinamide mononucleotide adenlyl transferase 1 (Nmnat1) and Ubiquitination factor e4b (Ube4b) [4]. While NAD^+^ levels normally plummet following injury [5], NAD^+^ depletion is blocked by Wld^S^ [6–9], hinting at a regulatory role for NAD^+^ in the decision to degenerate across evolution [10].

The discovery that Wallerian degeneration is an active destructive process prompted forward genetic screens for loss-of-function (LOF) mutants in which axons are protected following injury. Drosophila SARM1 was identified in such a screen as its loss confers robust protection of distal axons following axotomy [11]. Mice lacking SARM1 exhibit preservation of severed axons for weeks following injury [11,12], demonstrating conservation of function. Underscoring the importance of NAD^+^, SARM1 drives axonal death via intrinsic NAD^+^ hydrolase activity that is proposed to culminate in metabolic catastrophe [13,14]. *SARM1* encodes a protein with an N-terminal ARM domain, two tandem SAM domains, and a C-terminal TIR domain. Biochemical and genetic studies indicate that the TIR domain contains NAD^+^ hydrolase activity, the SAM domains are responsible for multimerization, and the ARM domain mediates auto-inhibition [12–16]. Recent structural studies provide a high-resolution view of SARM1 structure and demonstrate that it assembles into an octamer mediated by SAM domain oligomerization [17–19]. In its inactive conformation, the TIR domain is bound by the inhibitory ARM domain, while SARM1 activation leads to release of this auto-inhibition [18–22]. The TIR domains cleave NAD^+^ once released by the ARM domain in response to an increase in the NMN/NAD^+^ ratio [23].

SARM1 was first identified as an innate immune adaptor protein [24,25] and regulates neurodevelopment [16,26–30]. We recently demonstrated that dSARM is a component of a glial Toll receptor pathway required for clearance of neuronal corpses [29]. Loss of Toll-6 pathway components results in accumulation of apoptotic debris in the developing brain and early-onset neurodegeneration. The identification of this developmental function for dSARM raises important questions. To what extent are dSARM-mediated signaling mechanisms conserved between axon degeneration and glial phagocytic pathways? Specifically, is SAM domain-mediated multimerization and/or the NAD^+^ hydrolase activity of dSARM necessary for TLR-dependent signaling? And are dSARM’s downstream signaling mechanisms conserved in development and degeneration?

To date, functions of individual SARM1 domains have been assigned largely via *in vitro* assays and *in vivo* overexpression paradigms [12–15,17–22]. Given caveats associated with overexpression experiments, we interrogated SARM1 signaling requirements by mutating the endogenous locus. We used CRISPR/Cas9-mediated genome engineering to generate a *dSARM* knockout allele by replacing exons containing the ARM, SAM, and TIR domains with an attP recombination target. We then recombined in a series of domain mutants as well as a catalytically inactive point mutant (E1170A) and assessed the contributions of each domain to injury-induced degeneration and developmental phagocytosis. We find that the E1170A allele exhibits long-lived protection of axons following injury, demonstrating that dSARM’s NAD^+^ hydrolase activity accounts for its pro-degenerative function *in vivo*. Unexpectedly, the TIR-only allele can drive spontaneous axon degeneration over the course of days, indicating that SAM-mediated multimerization is not essential for dSARM activity in the absence of ARM domain-mediated inhibition. We next analyzed these new *dSARM* alleles in glial TLR signaling and find that dSARM’s NAD^+^ hydrolase activity is essential for this role. In contrast, the SAM domain is dispensable for signaling in glia. Finally, we explored signaling downstream of dSARM in glia and present evidence that dSARM functions through the MAP3K Ask1 in glia but not in degenerating axons. These findings argue that dSARM has distinct signaling modes in degenerative and non-degenerative signaling pathways.

## Results

### Genome engineering of the *dSARM* locus

**Fig 1:**
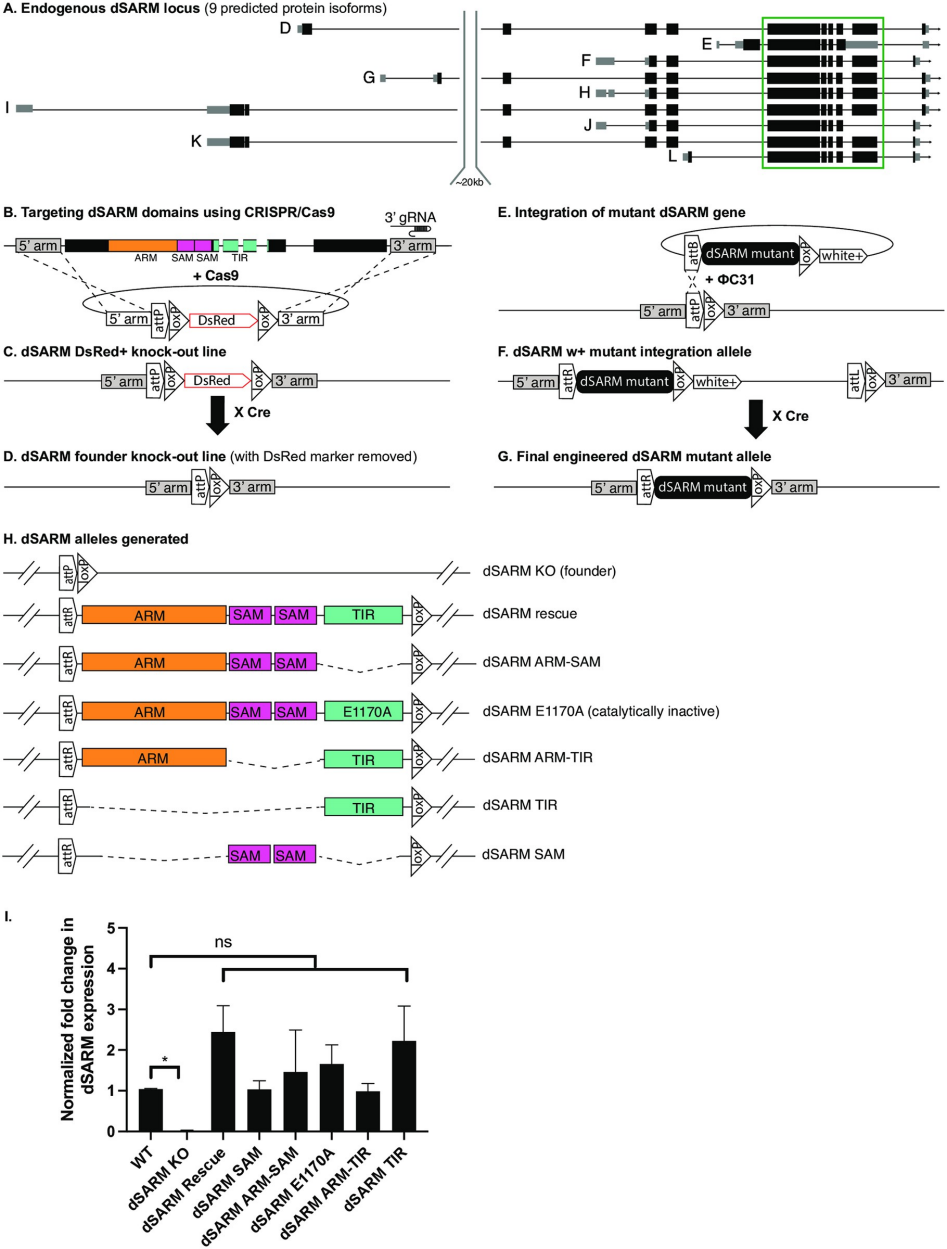
Genome engineering of the *dSARM* locus. (A) *dSARM* has 9 predicted isoforms. We designed a CRISPR/Cas9 strategy to knock out the ARM, SAM, and TIR domain encoding exons (boxed in green) in all isoforms. (B) A CRISPR/Cas9-induced homology-directed repair gene-targeting approach is used to delete the domain-encoding exons of *dSARM*. We identified a gRNA target sequence in the 3’ intron immediately following the exons in the green box in A. The homology-directed repair donor DNA plasmid contains 5’ and 3’ homologous arms flanking *dSARM* domains, a loxP-flanked DsRed selection marker, and an attP site of phiC31. The ARM (orange), SAM (magenta), and TIR (teal) domains are overlayed onto the dSARM locus. (C) In the “dSARM DsRed+ knock-out line”, the domain-encoding exons are replaced by the attP-loxP-DsRed-loxP cassette. (D) In the final *dSARM* founder knock-out line, the DsRed marker is removed by Cre recombinase, leaving attP and loxP sequences. (E) Genomic DNA is engineered to incorporate desired modifications (“*dSARM* mutant”) in the pGE-attB-w+ integration vector. The dSARM mutant is integrated into the founder line through phi-C31-mediated DNA integration via attP/attB recombination. (F) The resulting “dSARM w+ mutant integration allele” has the engineered mutant dSARM gene at its original genomic locus together with white+ and vector sequences. (G) w+ and extraneous vector sequences are removed by Cre recombinase to generate the “final engineered dSARM mutant allele” containing the engineered mutant flanked by attR and loxP sites in the adjacent introns. (H) A schematic showing the *dSARM* alleles that were generated. (I) qRT-PCR analysis of relative dSARM mRNA levels average ± SEM: wild type (Oregon R) (n = 28): 1.04±0.01; dSARM^KO^ (n = 4): 0.02±0.01; dSARM^Rescue^ (n = 4): 2.45±0.64; dSARM^E1170A^ (n = 4): 1.66±0.47; dSARM^ARM-SAM^ (n = 4): 1.46±1.03; dSARM^TIR^ (n = 4): 2.23±0.85; dSARM^ARM-TIR^ (n = 4): 0.99±0.18; dSARM^SAM^ (n = 4): 1.03±0.21 Error bars are SEM.

We sought to compare functional requirements of dSARM domains in developmental and degenerative contexts *in vivo* and so undertook a CRISPR/Cas9-mediated genome engineering approach. Using CRISPR/Cas9, we precisely deleted the ARM, SAM, and TIR domain-encoding exons of *dSARM* and replaced them with an attP site to create a founder knock-out allele (*dSARM*^*KO*^, Fig 1A–1D) [31,32]. We then utilized phiC31-mediated DNA integration at the attP site to create a series of *dSARM* alleles (Fig 1E–1G) [33]. These alleles retain endogenous intron-exon structure and differ only in the presence of a 50 nucleotide attR site in the intron preceding exon 17 and a 34 nucleotide loxP site in the intron succeeding exon 21/22 (exon numbering from FlyBase; Fig 1G). We successfully generated the following four domain mutants: *dSARM*^*ARM-TIR*^, *dSARM*^*ARM-SAM*^, *dSARM*^*TIR*^, and *dSARM*^*SAM*^, which are each named for the domain(s) present in the allele (Fig 1H). To test the function of dSARM’s NAD^+^ hydrolase activity in signaling, we mutated a key glutamic acid in the active site to alanine, which is equivalent to dSARM E893A in isoform E and human SARM1 E642A (*dSARM*^*E1170A*^; [14]; [34]. All of these new *dSARM* alleles are homozygous lethal. *dSARM*^*KO*^, *dSARM*^*ARM-TIR*^, *dSARM*^*ARM-SAM*^, *dSARM*^*SAM*^, and *dSARM*^*E1170A*^ animals die as wandering third-instar larvae (L3). The lethality of *dSARM*^*E1170A*^ animals indicates that the NAD^+^ hydrolase activity serves an essential developmental function. Interestingly, the lethal phase of *dSARM*^*TIR*^ homozygotes is at the first-instar larval stage (L1), suggesting that this allele has gain-of-function (GOF) activity. We also generated a *SARM*^*Rescue*^ line by recombining back in wild-type sequences (Fig 1H). *dSARM*^*Rescue*^ homozygotes are viable and fertile, serving as a control for the overall strategy. Alleles were validated by a combination of PCR and sequencing. Notably, after three rounds of injections into roughly 900 embryos, we were unable to recover *dSARM*^*SAM-TIR*^ transformants. This allele likely caused dominant lethality, as suggested by published work indicating that the ARM domain prevents unregulated activation of multimerized TIR domains [12,16]. To assay *dSARM* transcript abundance in mutant lines, we performed quantitative RT-PCR on first- or second-instar larvae, before the lethal phase of these animals. While *dSARM* transcripts are undetectable in the *dSARM*^*KO*^ founder line, we find normal levels of *dSARM* transcripts in all knock-in alleles (Fig 1I), indicating that stable transcripts are produced. Moreover, as shown below, we demonstrate intragenic complementation between pairs of alleles containing reciprocal domains demonstrating that the domain mutants are expressed and functional.

### Loss of dSARM’s NADase activity affords long-lived protection of injured ORN axons

We employed these *dSARM* alleles to test domain requirements of dSARM in axon degeneration. We undertook a MARCM-based approach to evaluate them in an olfactory receptor neuron (ORN) axotomy model, the same paradigm in which dSARM’s role in axon degeneration was first uncovered [11] In this genetic method, GFP-labeled homozygous mutant clones are generated in a heterozygous background. Since OR22a cell bodies are housed in the third antennal segment, distal axons can be readily detached from neuronal soma by removing the antenna. By performing a unilateral antennal ablation, the uninjured side serves as an internal control whereby the number of axons on the uninjured side are compared to the number on the injured side to quantify axon degeneration.

**Fig 2:**
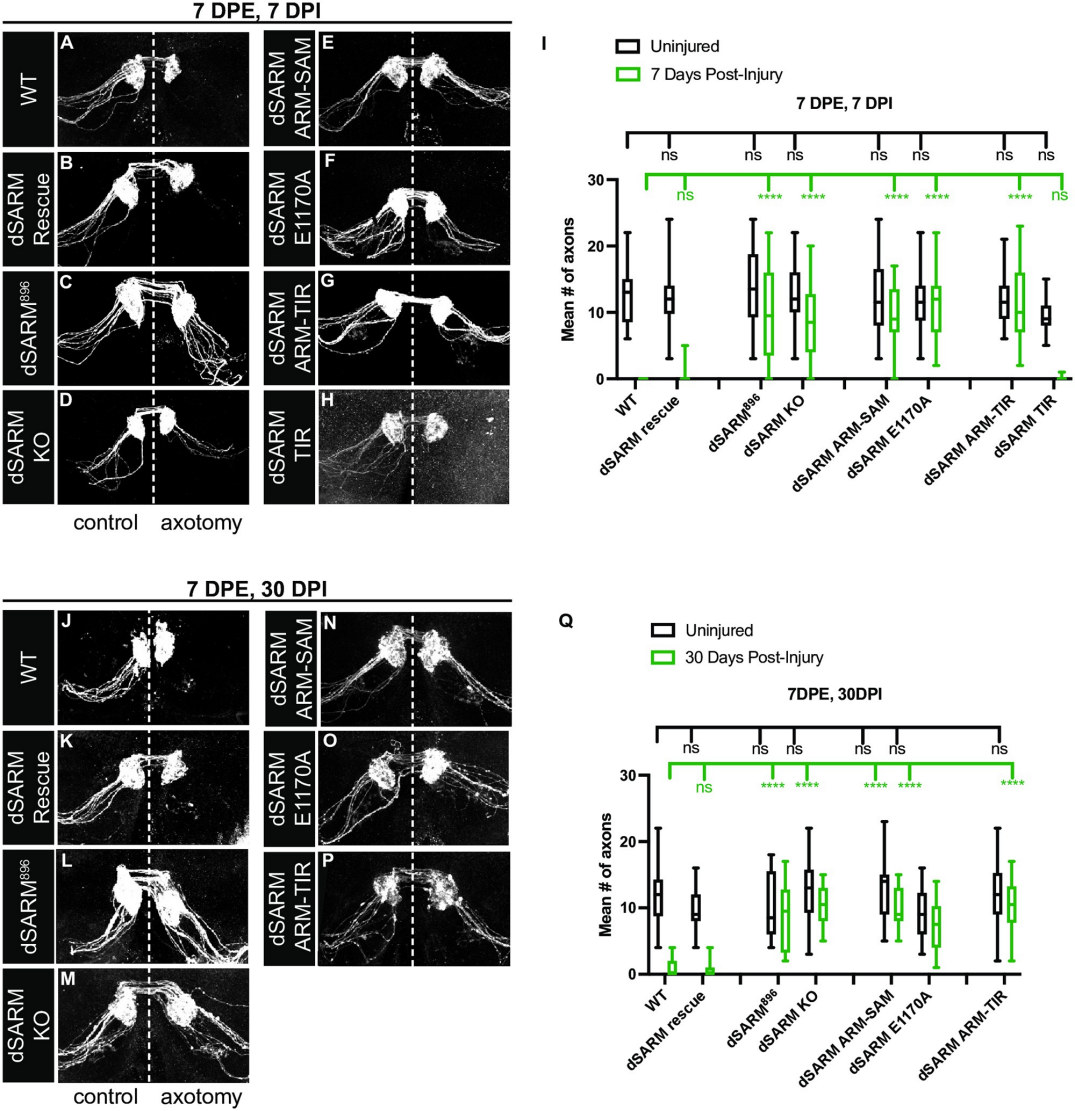
Loss of dSARM’s NADase activity affords long-lived protection of injured ORN axons. (A-H) Representative z-projections of OR22a ORNs of the indicated genotypes labeled with anti-GFP. The control side is on the left and the axotomized side is on the right. These animals were injured 7-day post-eclosion (7 DPE) and analyzed at 7-days post-injury (7 DPI). (I) Quantification of mean number of axons on uninjured control (black) and 7 days post-injury (green). Mean number of axons on uninjured control, injured sides: wild type (FRT2A) (n = 25): 12.32, 0.00; dSARM^896^ (n = 24): 13.92, 9.75; dSARM^Rescue^ (n = 30): 12.33, 0.40; dSARM^KO^ (n = 32): 12.56, 8.34; dSARM^ARM-SAM^ (n = 34): 12.42, 9.00; dSARM^E1170A^ (n = 24): 11.71, 11.26; dSARM^TIR^ (n = 35): 9.46, 0.04; dSARM^ARM-TIR^ (n = 24): 11.76, 10.89. (J-P) Representative z-projections of OR22a ORNs of the indicated genotypes labeled with anti-GFP. The control side is on the left and the axotomized side is on the right. These animals were injured 7 DPE and analyzed at 30 DPI. (Q) Quantification of mean number of axons on uninjured control (black) and 7 days post-injury (green). Mean number of axons on uninjured control, injured sides: wild type (FRT2A) (n = 30): 11.77, 1.10; dSARM^896^ (n = 20): 9.95, 8.80; dSARM^Rescue^ (n = 26): 9.27, 0.77; dSARM^KO^ (n = 28): 12.61, 10.29; dSARM^ARM-SAM^ (n = 22): 12.81, 10.00; dSARM^E1170A^ (n = 27): 9.32, 7.32; dSARM^ARM-TIR^ (n = 22): 11.91, 10.86. Error bars represent min and max data points. n.s. is not significantly different. ****, p < 0.0001.

We removed antennae in animals 7 days post-eclosion (DPE) and assessed axon protection 7 days post injury (DPI). To test if the knock-out/knock-in strategy itself interfered with wild-type dSARM function, we assayed whether homozygous *dSARM*^*Rescue*^ mutant clones display normal axon degeneration following injury (Fig 2A, 2B and 2I). We find complete axon degeneration at 7 DPI in *dSARM*^*Rescue*^ clones. In contrast, *dSARM*^*KO*^ clones display protection equivalent to a pre-existing *dSARM* allele (*dSARM*^*896*^; [11]; Fig 2C, 2D and 2I). Together, the behavior of the knockout and rescue alleles validates our strategy.

Structural and functional studies demonstrate that the TIR domain of SARM1 has intrinsic NADase activity and that this enzymatic activity is essential for axon degeneration [13–15,18,22,35,36]. Our new *dSARM* knock-in mutants enable critical *in vivo* tests of the function of dSARM’s catalytic activity and protein domains that to date have been largely assigned via *in vitro* assays and *in vivo* overexpression paradigms. We interrogated requirements of the TIR domain in general as well as the NAD^+^ hydrolase activity in particular [14]. We find that both *dSARM*^*ARM-SAM*^, in which the entire TIR domain is deleted, and *dSARM*^*E1170A*^, in which a key glutamic acid residue in the active site is mutated, provide perfect protection of distal axons at 7 DPI indicating a strict requirement for dSARM’s NADase activity in axon degeneration (Fig 2E, 2F and 2I). These findings differ somewhat from those in a recently published study [27] (see Discussion). We next tested whether SAM-mediated multimerization is required for degeneration by analyzing the phenotype of *dSARM*^*ARM-TIR*^ mutant clones. The SAM domains are important for dSARM’s pro-degeneration function *in vivo* since distal axons are fully protected in *dSARM*^*ARM-TIR*^ mutant axons (Fig 2G and 2I). Finally, we analyzed whether the TIR domain alone is sufficient for axon degeneration or whether it requires regulation via ARM and SAM domains. Surprisingly, *dSARM*^*TIR*^ mutant axons appear to exhibit timely degeneration of distal ORN axons (Fig 2H and 2I), indicating that the TIR domain alone is sufficient to drive injury-induced degeneration in this paradigm. However, the GFP fluorescence of both injured and un-injured ORN *dSARM*^*TIR*^ mutant clones is weaker than in controls (Fig 2H), a phenotype explored in more detail below.

We assayed the ability of our new *dSARM* alleles to provide extended protection to severed ORN axons at 30 days following an injury induced at 7 DPE. We find that both *dSARM*^*KO*^ and *dSARM*^*E1170A*^ mutant axons are fully protected from degeneration 30 DPI (Fig 2M, 2O and 2Q), demonstrating that the NADase activity of dSARM is essential for long-lived protection. *dSARM*^*ARM-TIR*^ mutant axons were also protected at 7 DPI (Fig 2G and 2I), but we wondered if this allele might retain low-level function reflected in axon loss at 30 DPI. However, *dSARM*^*ARM-TIR*^ mutant clones are also fully protected at 30 DPI (Fig 2P and 2Q), arguing that SAM-mediated multimerization is essential for dSARM activation when both ARM auto-inhibitory and TIR domains are present. These findings provide strong *in vivo* support for recent structural studies demonstrating that the NAD^+^ hydrolase activity of SARM1 is positively regulated by SAM-SAM domain interactions and negatively regulated by ARM domain-mediated inhibition [17–23].

### *dSARM*^*ARM-SAM*^ has dominant-negative activity following injury

**Fig 3:**
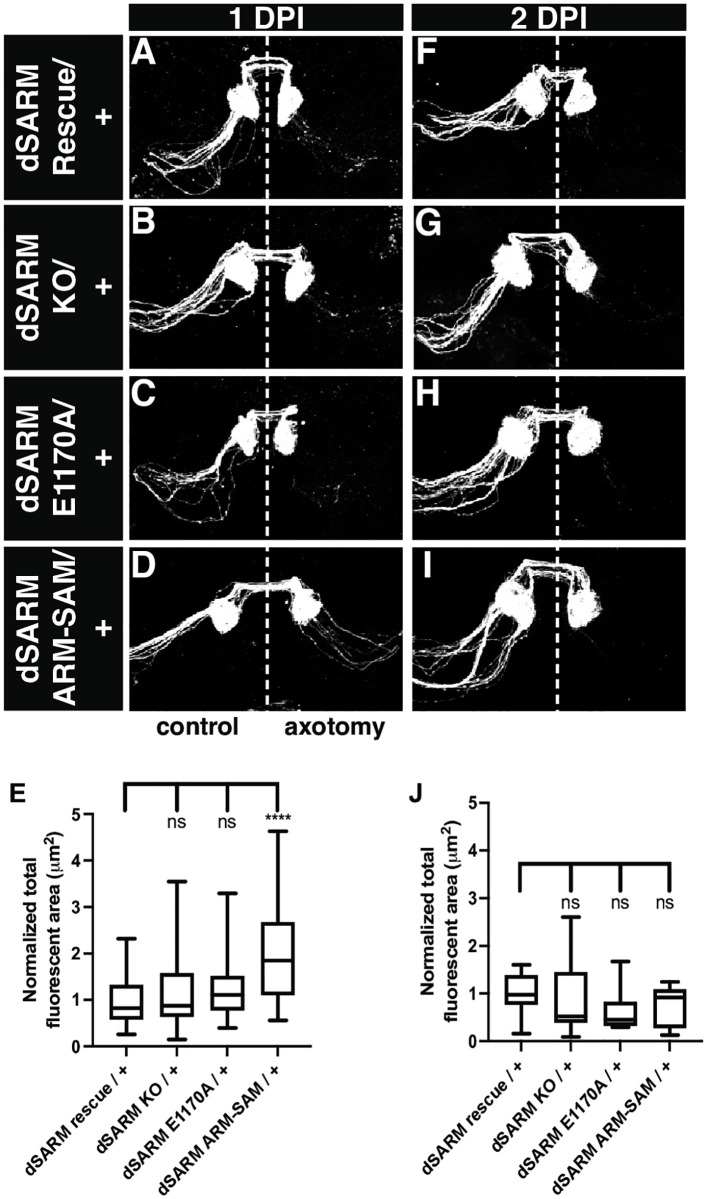
*dSARM*^*ARM-SAM*^ has dominant-negative activity following injury. (A-D, F-I) Representative z-projections of OR22a ORNs of the indicated genotypes labeled with anti-GFP. The control side is on the left and the axotomized side is on the right. These animals were injured 7 DPE and analyzed at 1 day (A-D) and 2 days (F-I) post-injury. (E) Quantification of total fluorescent area of debris on injured side at 1 DPI: dSARM^Rescue^ (n = 37): 1.00, dSARM^KO^ (n = 31): 1.16, dSARM^E1170A^ (n = 31): 1.27, and dSARM^ARM-SAM^ (n = 31): 2.07. (J) Quantification of total fluorescent area of debris on injured side at 2 DPI: dSARM^Rescue^ (n = 14): 1.00, dSARM^KO^ (n = 12): 0.90, dSARM^E1170A^ (n = 13): 0.62, and dSARM^ARM-SAM^ (n = 11): 0.72. Error bars represent min and max data points. n.s. is not significantly different. ****, p < 0.0001.

The octameric structure of SARM1 raises the possibility that mutants disrupting domain stoichiometry might display dominant-negative effects by inhibiting TIR domain multimerization [12]. Thus, we tested if reducing the number of TIR domains relative to ARM and SAM domains would slow axon degeneration. Normally, degeneration of distal ORN axons is efficient, with little debris remaining by 24 h post-injury (1 DPI; Fig 3A). Instead of counting axons, we quantified total axonal debris in these experiments to better capture axon fragmentation observed shortly after injury.

Using OR22aGal4 to drive UAS-mCD8::GFP in ORNs, we find that axons in whole animal *dSARM*^*KO*^ heterozygotes degenerate as quickly as in controls, indicating that loss of one copy of dSARM does not delay axon degeneration (Fig 3B and 3E). We next tested whether *dSARM*^*E1170A*^ or *dSARM*^*ARM-SAM*^ heterozygous axons exhibit delayed degeneration since they alter either TIR domain number (*dSARM*^*ARM-SAM*^) relative to ARM-SAM domains or TIR domain enzymatic activity (*dSARM*^*E1170A*^). Interestingly, while *dSARM*^*E1170A*^ heterozygotes degenerate as rapidly as controls, axon degeneration in *dSARM*^*ARM-SAM*^ heterozygotes is incomplete at 1 DPI (Fig 3A, 3C, 3D and 3E). Axons do degenerate in all backgrounds by 2 DPI (Fig 3F–3J). We propose that *dSARM*^*ARM-SAM*^ has modest dominant-negative activity because each octamer contains more auto-inhibitory ARM domains than TIR domains, which delays the formation of TIR-TIR dimers. The most parsimonious explanation of the finding that *dSARM*^*E1170A*^ is not a dominant-negative allele is that loss of NADase activity in one TIR monomer does not interfere with the NADase activity of other TIR monomers.

### *dSARM*^*TIR*^ mutant ORN clones exhibit injury-independent axon degeneration

While imaging 7 DPE animals, we noticed that both the uninjured and injured *dSARM*^*TIR*^ clones were less bright than the other genotypes and required more laser power to acquire an equivalent image, suggesting the possibility that the TIR domain alone caused axon degeneration. This prompted us to quantify the intensity of mutant clones for all alleles over time in the absence of injury.

**Fig 4:**
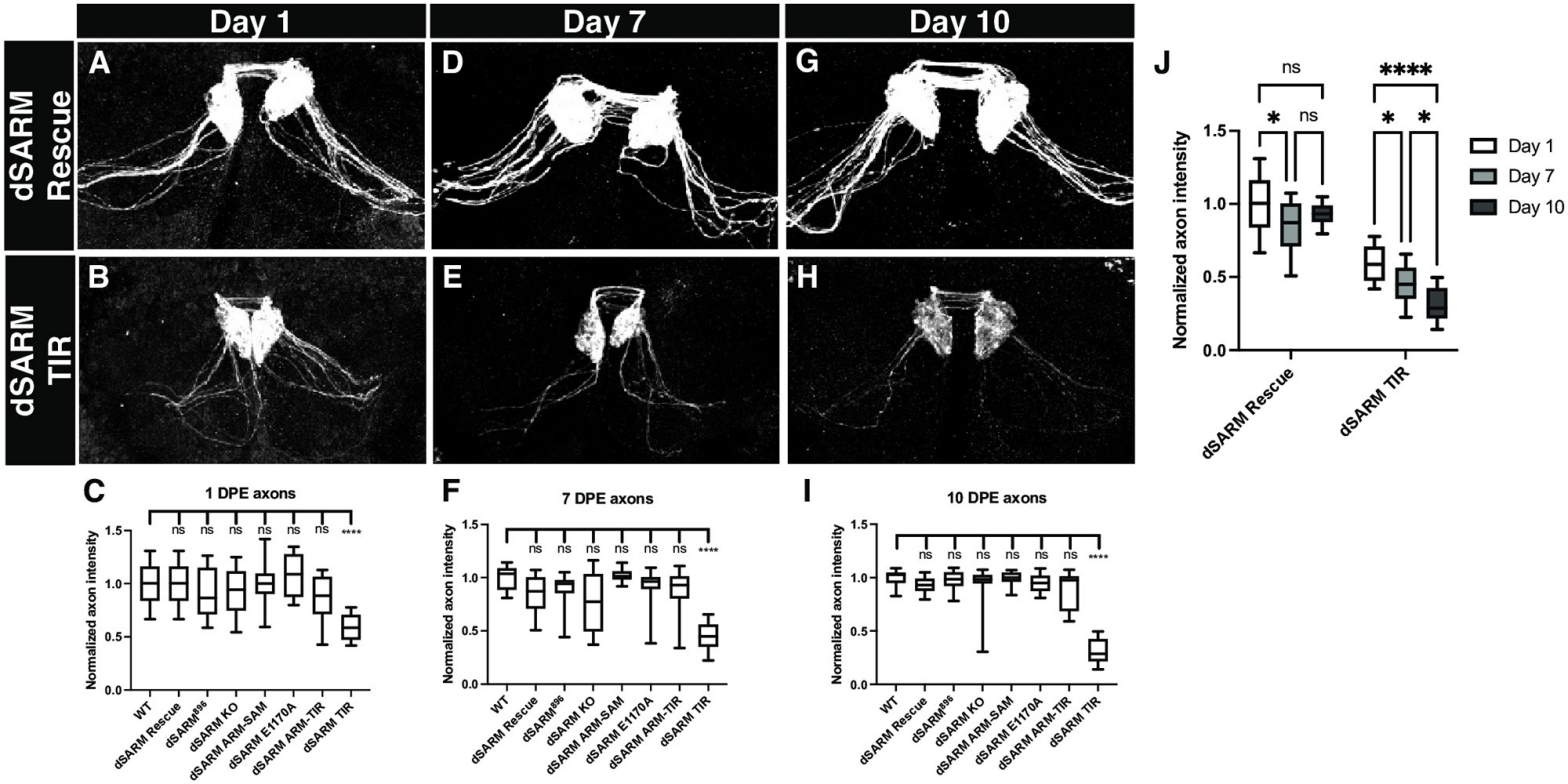
*dSARM*^*TIR*^ mutant clones exhibit injury-independent axon degeneration. (A,B) Representative z-projections of OR22a ORNs of the indicated genotypes labeled with anti-GFP at 1DPE. (C) Normalized mean axon intensity at 1 DPE: wild type (FRT2A): 1.00, dSARM^896^: 0.90, dSARM^Rescue^: 1.00, dSARM^KO^: 0.92, dSARM^E1170A^: 1.09, dSARM^ARM-SAM^: 1.00, dSARM^TIR^: 0.59, and dSARM^ARM-TIR^: 0.88. (D,E) Representative z-projections of OR22a ORNs of the indicated genotypes labeled with anti-GFP at 7 DPE. (F) Normalized mean axon intensity at 7 DPE: wild type (FRT2A): 1.00, dSARM^896^: 0.88, dSARM^Rescue^: 0.85, dSARM^KO^: 0.77, dSARM^E1170A^: 0.92, dSARM^ARM-SAM^: 1.03, dSARM^TIR^: 0.45, and dSARM^ARM-TIR^: 0.86. (G,H) Representative z-projections of OR22a ORNs of the indicated genotypes labeled with anti-GFP at 10 DPE. (I) Normalized mean axon intensity at 10 DPE: wild type (FRT2A): 1.00, dSARM^896^: 0.98, dSARM^Rescue^: 0.93, dSARM^KO^: 0.93, dSARM^E1170A^: 0.94, dSARM^ARM-SAM^: 0.99, dSARM^TIR^: 0.30, and dSARM^ARM-TIR^: 0.88. (J) Normalized mean axon intensity over time in dSARM^Rescue^ compared to dSARM^TIR^. dSARM^Rescue^: 1.00 (1DPE), 0.85 (7DPE), and 0.93 (10 DPE) and dSARM^TIR^: 0.59 (1 DPE), 0.45 (7DPE), and 0.30 (10 DPE). Error bars represent min and max data points. N = 16 for each genotype. n.s. is not significantly different. *, p < 0.05. ****, p < 0.0001.

First, we analyzed at 1 DPE (Fig 4A–4C) and found that *dSARM*^*TIR*^ mutant axons were already approximately 40% less bright than controls. Next, we looked at 7 DPE and found that *dSARM*^*TIR*^ mutant clones were approximately 55% less bright than controls (Fig 4D–4F). At 10 DPE the appearance of *dSARM*^*TIR*^ axons continued to wane, with an intensity 70% less than that of control genotypes (Fig 4G–4I). None of the other *dSARM* alleles affected fluorescence intensity at any of these time points. When we compared axon intensity among the three time points for *dSARM*^*TIR*^ mutant clones, we found a steady loss of axon integrity (Fig 4J), indicating that without ARM and SAM domains, the TIR domain drives axon loss over the course of 10 days. We speculate that without ARM domain-mediated inhibition, the TIR domains are free to associate with each other, cleave NAD^+^ and drive degeneration, but do so over a slower time scale then when they are tethered together by SAM domains. Together, these data argue that in ORN axons, SAM domain-mediated TIR multimerization drives high-level NADase activity to drive rapid axon degeneration on the order of hours following an acute injury. On the other hand, TIR monomers, or low frequency formation of TIR dimers, have low-level NADase activity capable of spontaneous axon degeneration over the course of 10 days.

We were intrigued by the unexpected finding that *dSARM*^*TIR*^ mutant ORN clones exhibit injury-induced degeneration (Fig 2H–2I). To look more carefully at the timing of injury-induced degeneration in these clones, we assessed axon degeneration 12 hours after injury. We performed the experiment at 1 DPE so that the mutant clones still appeared relatively healthy and quantified axons 12 h following axotomy in order to uncover small differences in timing. Surprisingly, we found no difference in degeneration rate in *dSARM*^*TIR*^ mutant clones relative to *dSARM*^*Rescue*^ clones (S1A–S1C Fig), arguing that free TIR domains can support timely injury-induced axon degeneration in neurons that are already undergoing slow and steady spontaneous degeneration.

### All *dSARM* domains are required for degeneration of injured wing sensory axons

**Fig 5:**
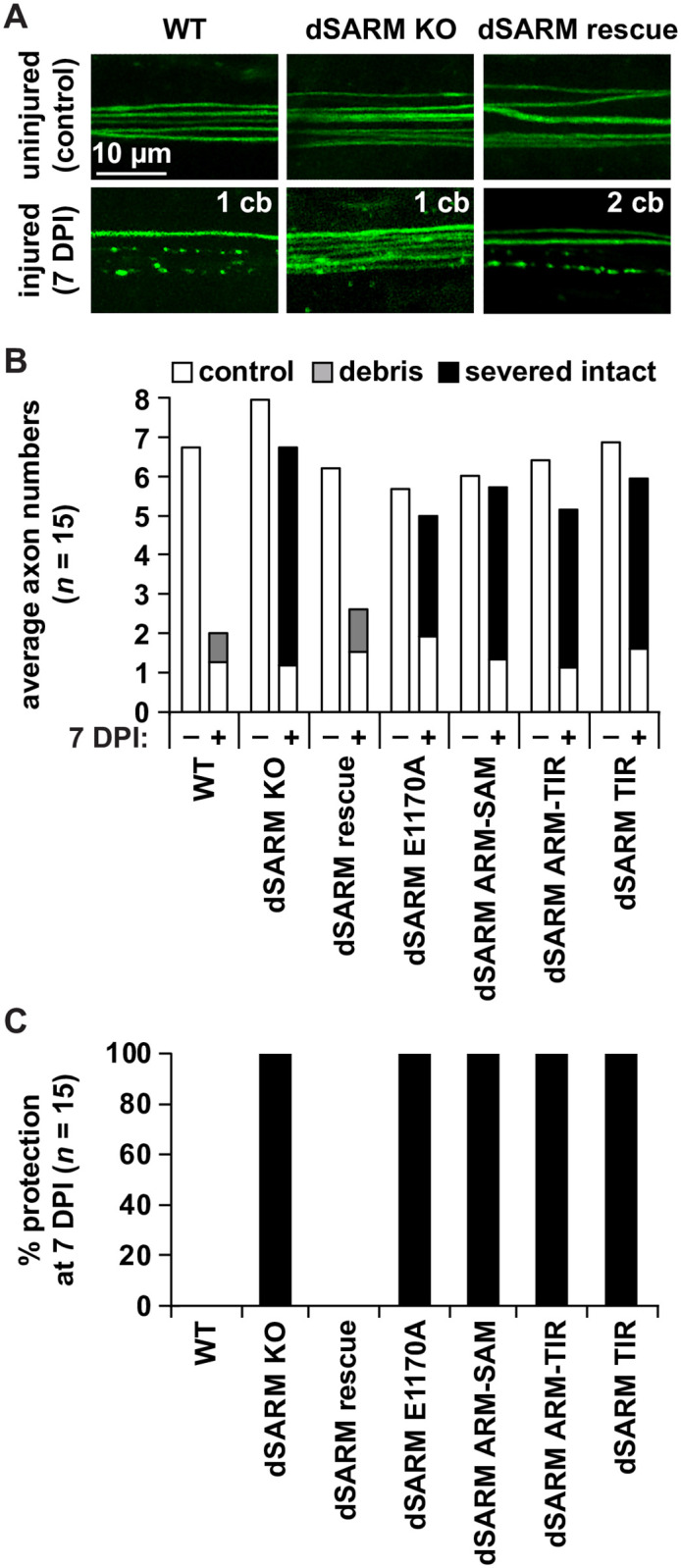
Full length dSARM is needed to trigger axon degeneration. (A) Representative pictures of control and 7 DPI axons of WT, dSARM KO and dSARM rescue. (B) Quantification of average axonal scores of uninjured control, debris, and severed intact axons (white, gray, and black, respectively). (C) Percentage of preserved severed axons at 7 DPI. N = 15 animals for each genotype.

To extend the observations in the ORN injury assay in a second axotomy model, we tested our engineered mutations in a previously described MARCM-based approach in wing sensory neurons [37]. In this paradigm, flies are aged for 5–7 DPE before being subjected to injury: one wing is partially injured, while the other serves as an uninjured control [38]. At the day of injury, the number of cell bodies is counted in the cut-off wing, which indicates how many axons are severed. At 7 DPI, the injured and control wings are assessed for axonal phenotypes. In the injured examples, the number of neuronal cell bodies (cb) that were not cut off are indicated in the upper right corner as readout of uninjured axons (Fig 5A). The phenotypes (e.g., uninjured control axons, axonal debris, and severed intact axons, respectively) were quantified (Fig 5B), and the percentage of protected severed axons assessed at 7 DPI (Fig 5C).

We observed 100% protection of injured axons in *dSARM*^*KO*^ clones, while axons in *dSARM*^*rescue*^ clones fully degenerated (Fig 5A, 5B and 5C). *dSARM*^*E1170A*^ and *dSARM*^*ARM-SAM*^ also failed to execute injury-induced axon degeneration, further confirming the necessity of the NAD^+^ hydrolase activity in axon degeneration (Fig 5B and 5C). *dSARM*^*ARM-TIR*^ mutant clones displayed equivalent axon protection to *dSARM*^*KO*^, supporting that SAM-SAM multimerization is indispensable for dSARM activation after injury (Fig 5B and 5C). Lastly, we investigated the behavior of the TIR-only *dSARM* allele, which displayed both spontaneous and injury-induced axon degeneration in injured ORNs. In *dSARM*^*TIR*^ wing mutant clones, we did not observe signs of spontaneous degeneration, and clones showed full protection at 7 DPI (Fig 5B and 5C). These findings suggest that in the absence of ongoing spontaneous degeneration as in ORNs, the TIR domain does not support rapid injury-induced degeneration. Why the TIR-only mutant behaves differently in olfactory receptor neurons versus wing sensory neurons remains an open question that we speculate relates to differential timing of clone induction in the two systems (see Discussion). Taken together, these findings indicate that the NADase activity and SAM domain-mediated oligomerization are necessary for dSARM to activate and function as a neurodegenerative agent in injury-induced axon degeneration.

### dSARM signaling in glia requires its NADase activity, but not its SAM domains

We recently uncovered a requirement for a glial dSARM-mediated TLR pathway in clearing neuronal debris during development. In loss-of-function (LOF) mutants of Toll-6, FoxO or dSARM, levels of Dcp-1-positive apoptotic debris are increased in the L3 brain [29]. We demonstrated that this pathway promotes phagocytosis by activating transcription of the key engulfment receptor Draper (Drpr) in a specific population of neuronal cell body-associated glia called cortex glia [39]. The discovery of a function for dSARM as a TLR pathway component raised important questions: (1) is dSARM multimerization required for TLR signaling? (2) does dSARM act solely as a TIR adaptor in this pathway or is its NAD^+^ hydrolase activity required? And (3) to what extent is signaling downstream of dSARM conserved in development versus degeneration? We previously demonstrated that RNAi-mediated knockdown of *dSARM* in cortex glia results in an identical increase in Dcp-1 debris as observed in *dSARM* nulls, while pan-neuronal dSARM knockdown does not affect corpse clearance [29]. Thus, the Dcp-1 phenotype observed in *dSARM* alleles can be attributed solely to dSARM’s function in cortex glia.

**Fig 6:**
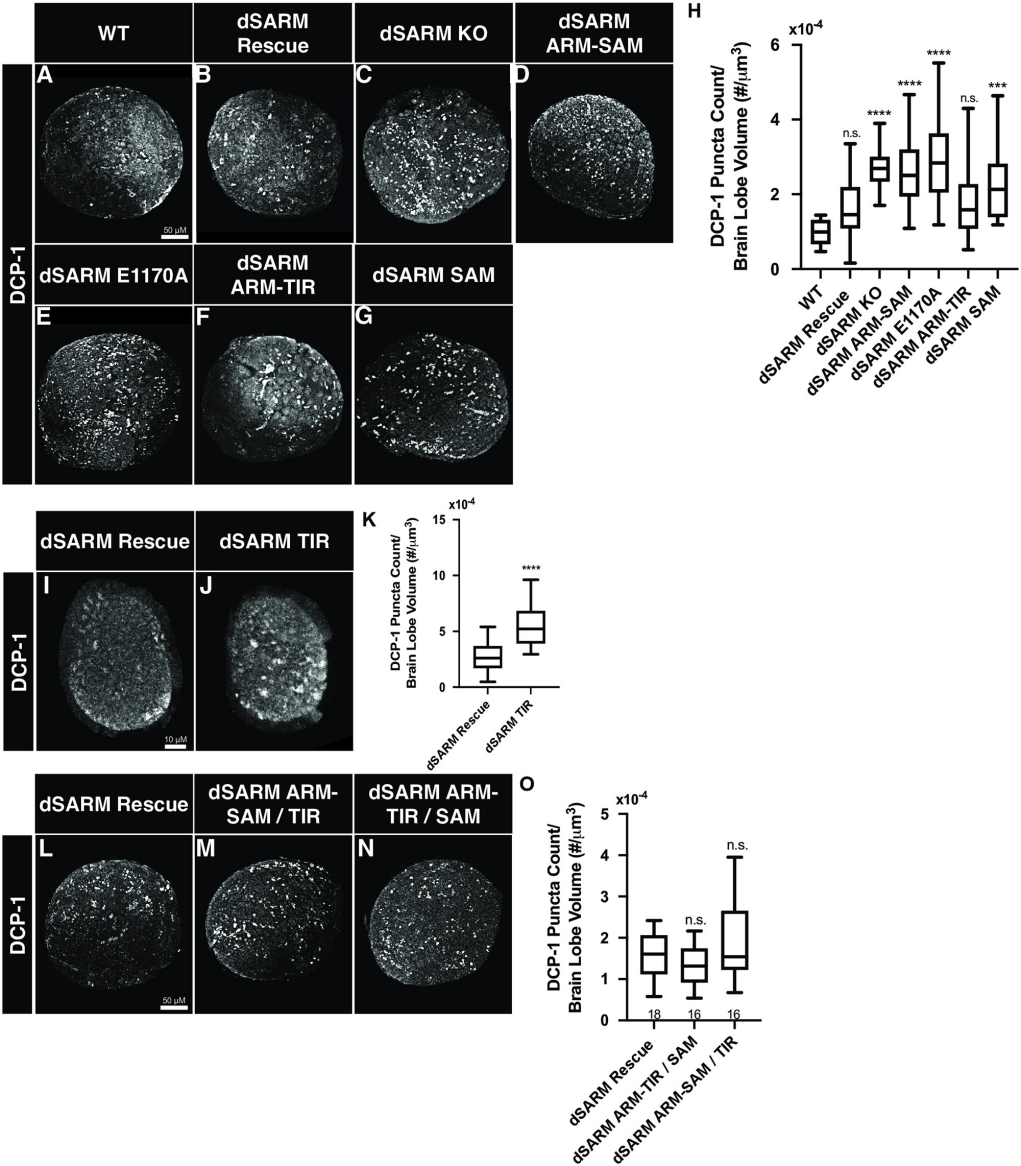
dSARM signaling in glia requires its NADase activity, but not its SAM domains. (A-G, I-J, and L-N) Representative z-projections of brain lobes of the indicated genotypes labeled with anti-Dcp-1. (H) Quantification of the number of Dcp-1 puncta normalized to brain lobe volume: wild type (Oregon R) (n = 28): 9.82x10^-5^, dSARM^Rescue^ (n = 80): 1.64x10^-4^, dSARM^KO^ (n = 30): 2.73x10^-4^, dSARM^ARM-SAM^ (n = 20): 2.69x10^-4^. dSARM^E1170A^ (n = 28): 2.91x10^-4^, dSARM^ARM-TIR^ (n = 38): 1.75x10^-4^, and dSARM^SAM^ (n = 17): 2.35x10^-4^. (K) Quantification of the number of Dcp-1 puncta normalized to brain lobe volume: dSARM^Rescue^ (n = 26): 2.61x10^-4^, and dSARM^TIR^ (n = 34): 5.22x10^-4^. (O) Quantification of the number of Dcp-1 puncta normalized to brain lobe volume: dSARM^Rescue^ (n = 18): 1.57x10^-4^, dSARM^ARM-SAM/TIR^ (n = 16): 1.92x10^-4^, and dSARM^ARM-TIR/SAM^ (n = 16): 1.32x10^-4^. n values can be found on each graph. n.s., not significant; *, p < 0.05; **, p < 0.01; ***, p < 0.001; ****, p < 0.0001.

To investigate functional requirements of individual dSARM domains in this pathway, we quantified the amount of Dcp-1 debris in L3 brains in all new *dSARM* alleles. For all alleles except *dSARM*^*TIR*^ (see below), we conducted this analysis at early L3 before the lethal phase of these animals. We developed an Imaris imaging pipeline to automate and standardize debris quantification, where to account for differences in brain size, Dcp-1 puncta count is normalized to individual brain lobe volume (see Materials and methods; Dcp-1 puncta visible as white spots in brain lobes in Fig 6). Using this method, we find that neuronal corpses are cleared normally in *dSARM*^*Rescue*^ animals as evidenced by normal levels of Dcp-1 punta in this background (Fig 6A, 6B and 6H). In contrast, we observe a roughly two-fold increase in apoptotic debris in *dSARM*^*KO*^ homozygotes relative to *dSARM*^*Rescue*^ animals (Fig 6C and 6H). This phenotype is consistent with that observed in the original *dSARM* LOF alleles and also with RNAi-mediated *dSARM* knockdown in cortex glia [29]. The phenotypes of the rescue and knockout in apoptotic debris clearance establishes the utility of our dSARM allelic series in dissecting signaling requirements of dSARM domains in this glial pathway.

We continued by interrogating the contributions of individual dSARM domains to developmental signaling. We find excessive neuronal debris in both *dSARM*^*ARM-SAM*^ and *dSARM*^*SAM*^ homozygotes (Fig 6D, 6G and 6H), indicating a TIR domain requirement in TLR signaling. Moreover, *dSARM*^*E1170A*^ homozygotes display an equivalent increase in Dcp-1 debris (Fig 6E and 6H), indicating that the NAD^+^ hydrolase activity is essential for the signaling role of dSARM in this setting. We next tested whether SAM-mediated dSARM multimerization is required by quantifying Dcp-1 puncta in *dSARM*^*ARM-TIR*^ homozygotes. Strikingly, apoptotic debris remains at control levels in *dSARM*^*ARM-TIR*^ mutants (Fig 6F and 6H), indicating that the SAM domain is dispensable for dSARM’s signaling role. We wanted to test whether *dSARM*^*TIR*^ homozygotes display a glial phagocytosis phenotype at the L3 stage, but these mutants do not live this stage of development. Thus, we quantified apoptotic debris at L1. *dSARM*^*TIR*^ homozygotes display a roughly two-fold increase in neuronal debris relative to *dSARM*^*Rescue*^ animals at this stage (Fig 6I–6K). Given the likely GOF activity observed in *dSARM*^*TIR*^ mutants, it is unclear whether the increased debris in these animals reflects dSARM function in cortex glia or increased neuronal death caused by a different mechanism. Regardless, these data imply that isolated TIR domains are insufficient to carry out dSARM’s function in cortex glia. To test if *dSARM*^*TIR*^ behaves as a dominant allele in this assay, we tested if *dSARM*^*TIR*^ heterozyogtes display increased levels of Dcp-1 debris at the L3 stage. We find normal levels of Dcp-1 debris in *dSARM*^*TIR*^ heterozygotes and in the rest of our new dSARM alleles (S3 Fig), demonstrating that none of the alleles have dominant activity in this context. These experiments suggest two main conclusions. (1) The finding that *dSARM*^*ARM-TIR*^ behaves as a null in injury-induced axon degeneration yet supports developmental signaling indicates a differential requirement for the SAM domains these two contexts. (2) The enzymatic activity of dSARM is essential for signaling, thus extending the known roles of the NADase activity of dSARM to signal transduction.

In pathological axon degeneration, the ARM, SAM, and TIR domains have all been assigned unique, separable functions. The extent to which these domains are distinct functional elements in TLR signal transduction has not been investigated. Intragenic complementation provides a classic genetic test of domain separability [40], and the generation of a series of complementary domain mutants of dSARM (Fig 1H) provides a unique opportunity to investigate this question. We generated *dSARM*^*ARM-SAM*^*/dSARM*^*TIR*^ and *dSARM*^*ARM-TIR*^*/dSARM*^*SAM*^ heteroallelic animals to test whether the domains can complement each other and restore wild-type dSARM function. We find that heteroallelic combinations of both pairs of reciprocal mutants are viable until the mid-pupal stage, while all homozygous mutants die as wandering third-instar larvae. The finding that ARM-SAM suppresses the early lethality observed in TIR-only homozygotes argues that ARM-SAM inhibits the GOF activity observed in this allele in trans. To look more specifically at the function of these reciprocal pairs of mutants in signaling, we quantified Dcp-1 apoptotic debris. We find that Dcp-1 counts are at wild-type levels in both heteroallelic combinations (Fig 6L–6O). This result is not surprising in the case of *dSARM*^*ARM-TIR*^*/dSARM*^*SAM*^, since *dSARM*^*ARM-TIR*^ homozygotes do not display a debris clearance phenotype. However, the rescue of debris clearance in *dSARM*^*ARM-SAM*^*/dSARM*^*TIR*^ animals indicates that the ARM and TIR domains need not be covalently bound to restore wild-type *dSARM* function in a TLR pathway. Together, these findings indicate that the ARM, SAM, and TIR domains have separable functions during development and that the ARM domain can restrain the activity of the TIR domain in trans.

### The MAP3K Ask1 is required for glial TLR signaling, but not for axon degeneration

The discovery that dSARM’s NADase activity is required for pro-phagocytic signaling begs the question of how information is transmitted by dSARM’s enzymatic activity. As a first step toward answering this question, we sought to identify downstream signaling components. We hypothesized that dSARM activation engages a MAPK cascade in cortex glia, a model initially based on Toll-6 signal transduction in motorneurons, where Toll-6 and dSARM drive JNK activation [30]. We looked for evidence of MAPK involvement in cortex glia, focusing first on the Drosophila MAP3K Ask1. Ask1 was a particularly attractive candidate because elegant genetic analyses in *C*. *elegans* demonstrated that Tir-1 (the worm homolog of dSARM/SARM1) activates Nsy-1 (the worm homolog of Ask1) to specify asymmetric odorant receptor expression [16]. More recently, dSARM and Ask1 have both been shown to be required to block vesicle trafficking after nerve injury [27].

**Fig 7:**
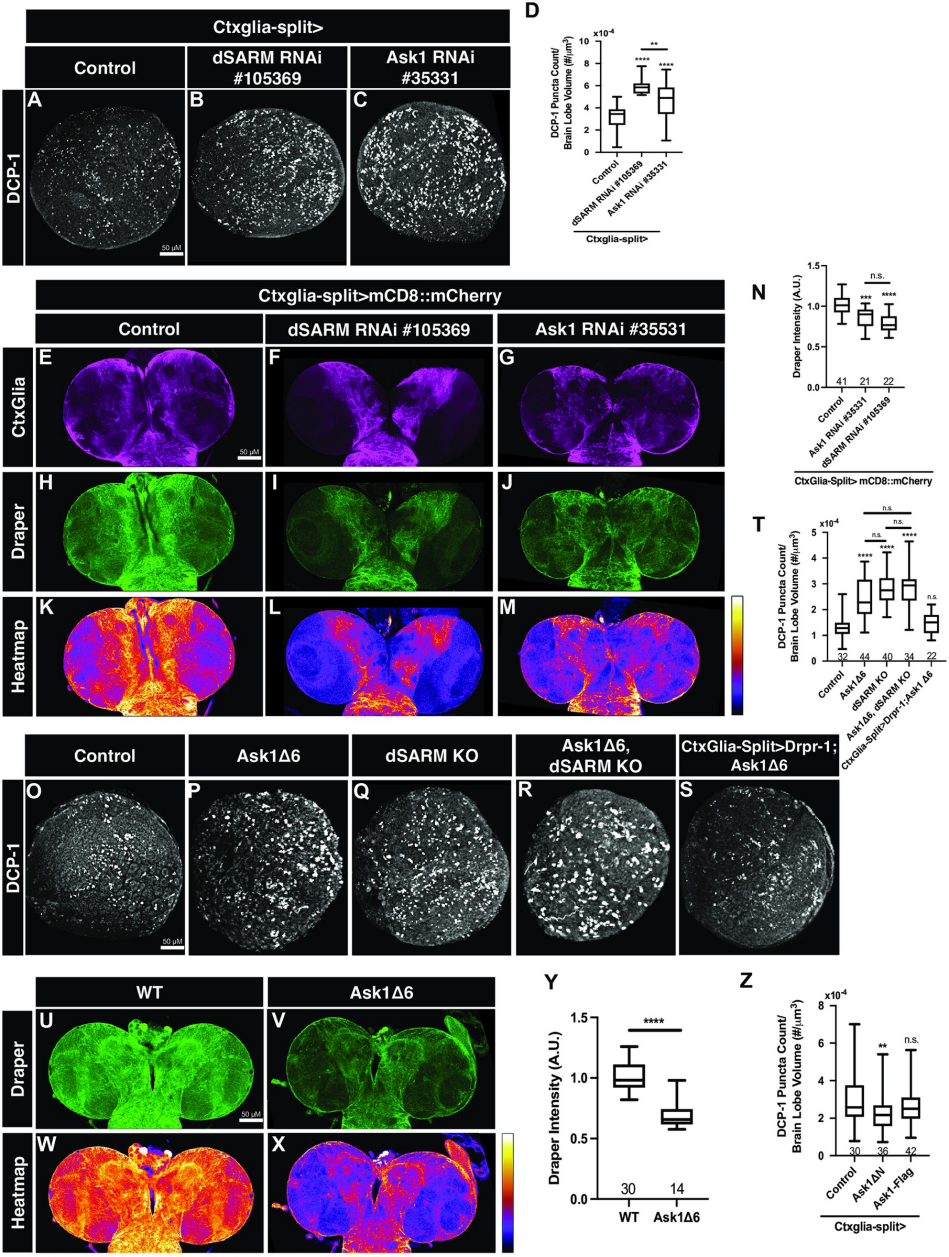
dSARM and Ask1 are in a linear genetic pathway in cortex glia. (A-C) Representative z-projections of brain lobes of the indicated genotypes labeled with anti-Dcp-1. (D) Quantification of the mean number of Dcp-1 puncta per brain lobe normalized to brain lobe volume: Control (Ctxglia-split>OR) (n = 44): 3.05x10^-4^, Ctxglia-split>dSARM RNAi #105369 (n = 18): 5.98x10^-4^, and Ctxglia-split>Ask1 RNAi #35331 (n = 42): 4.62x10^-4^. (E-G) Representative 3 mm z-projections of larval brains of indicated genotypes labeled with anti-mCherry. (H-J) Representative 3 mm z-projections of larval brains of indicated genotypes labeled with anti-Drpr. (K-M) Representative 3 mm z-projections of larval brains of indicated genotypes labeled with anti-Drpr represented as a heatmap. (N) Quantification of mean Drpr intensity normalized to Control (Ctxglia-split>mCD8::mCherry, LacZ): 1.00, Ctxglia-split>mCD8::mCherry, Ask1 RNAi #35331: 0.86, and Ctxglia-split>mCD8::mCherry, dSARM RNAi #105369: 0.75. (O-S) Representative z-projections of brain lobes of the indicated genotypes labeled with anti-Dcp-1. (T) Quantification of the mean number of Dcp-1 puncta per brain lobe normalized to brain lobe volume: Control (Oregon R): 1.28x10^-4^, Ask1Δ6: 2.42x10^-4^, dSARM KO: 2.81x10^-4^, and Ask1Δ6, dSARM KO: 2.78x10^-4^, and Ctxglia-split>Drpr-1; Ask1Δ6: 1.45x10^-4^. (U,V) Representative 3 mm z-projections of larval brains of indicated genotypes labeled with anti-Drpr. (W,X) Representative 3 mm z-projections of larval brains of indicated genotypes labeled with anti-Drpr represented as a heatmap. (Y) Quantification of mean Drpr intensity normalized to control: Control (Oregon R): 1.00 and Ask1Δ6: 0.70. (Z) Quantification of the mean number of Dcp-1 puncta per brain lobe normalized to brain lobe volume: Ctxglia-split>Control (Oregon R): 2.99x10^-4^, Ctxglia-split>Ask1ΔN: 2.22x10^-4^, and Ctxglia-split>Ask1-Flag: 2.66x10^-4^. n values can be found on each graph. n.s., not significant; *, p < 0.05; **, p < 0.01; ***, p < 0.001; ****, p < 0.0001.

To test for Ask1 involvement in glial TLR signaling, we investigated whether RNAi-mediated silencing of Ask1 in cortex glia increases Dcp-1 debris in the L3 brain. Indeed, reducing Ask1 expression in cortex glia resembles loss of dSARM and results in a two-fold increase in Dcp1 puncta (Fig 7A–7D). Glial TLR signaling promotes efficient phagocytosis via upregulation of Drpr [29,41], so we investigated whether cortex glial silencing of Ask1 resulted in reduced Drpr levels comparable to that of other pathway members. We find that RNAi-mediated loss of Ask1 in cortex glia reduces Drpr expression levels in cortex glia comparable to loss of dSARM (Fig 7E–7N). Since an *ask1* null allele did not exist, we employed CRISPR to generate a 6 KB deletion of the *ask1* locus to enable a genetic analysis (*ask1*^*Δ6*^; see Materials and methods). We find that loss of Ask1 results in elevated Dcp-1 puncta and mimics loss of *dSARM* (Fig 7O–7Q and 7T). Loss of Ask1 also results in decreased baseline Drpr levels (Fig 7U–7Y), without changing the gross morphology of cortex glia. The phenotypic similarities between *dSARM* and *ask1* nulls are consistent with the idea that the genes are in the same genetic pathway. Indeed, *ask1*^*Δ6*^ *dSARM*^*KO*^ double mutants display similar levels of Dcp-1 debris to either of the individual single mutants (Fig 7P–7R and 7T), providing genetic evidence that the genes function in a linear pathway. We previously demonstrated that Drpr overexpression in cortex glia is sufficient to suppress the phagocytosis phenotypes observed in *dSARM* mutants [29]. If Ask1 is required in this signaling pathway, Drpr overexpression should also suppress the *ask1* Dcp-1 phenotype. Indeed, cortex glial Drpr overexpression restores normal debris clearance to *ask1* homozygotes (Fig 7O, 7R and 7S), consistent with the identification of Ask1 as a novel component of this TLR pathway.

Ask1 is a conserved MAP3K in innate immune pathways where it is key to defense systems activated in response to harmful stimuli [42,43]. Ask1 orthologs contain an inhibitory N-terminal domain, and Ask1 proteins lacking this domain can be constitutively active (Ask1ΔN, [42]. We previously found that Toll-6 overexpression in glia is sufficient to decrease neuronal debris, presumably as a result of accelerated debris clearance [29]. Thus, we wondered if overexpression of Ask1 or Ask1ΔN might speed engulfment. We find that cortex glial overexpression of Ask1ΔN, but not full-length Ask1, results in a significant reduction in the number of Dcp1 puncta in the brain relative to controls (Fig 7Z). These findings underscore the involvement of Ask1 in glial TLR signaling and argue that the N-terminal domain of Ask1 plays an inhibitory role in regulating Ask1 activation.

After demonstrating a function for Ask1 in TLR signaling in glial phagocytosis, we were curious as to whether Ask1 contributes to axon degeneration. Thus, we asked whether either loss or overexpression of Ask1 protected ORN axons following axotomy. Here, we quantified total axonal debris to establish any possible delay in axon fragmentation. In the absence of Ask1, we do not see a change in axonal debris indicating there is no delay in degeneration (S2A–S2C Fig). Similarly, when we overexpress wild-type Ask1 or constitutively active Ask1ΔN, we do not see change in the rate of axonal fragmentation (S2D–S2G Fig). These findings demonstrate that Ask1 does not play a role in axon degeneration. Taken together, these findings indicate that Ask1 plays a role in developmental TLR signal transduction, but not in axon degeneration.

## Discussion

Mechanisms of TLR signal transduction are many, but a common feature is that intracellular TIR domains of TLR receptors engage TIR domain-containing adaptors [44]. In TLR pathways, TIR domains have been considered solely as protein-protein interaction domains. We recently defined a glial function for the TIR adaptor dSARM downstream of a TLR that promotes engulfment of neuronal corpses during development [29]. We wondered to what extent dSARM’s contribution to TLR signaling is related to its function in pathological axon degeneration [11,13,14]. To address this question, we generated a series of dSARM domain and point mutants via CRISPR/Cas9-mediated genome engineering. We compared the behavior of these new *dSARM* alleles in development and degeneration and find that while dSARM’s enzymatic activity is essential in both contexts, SAM domain-mediated multimerization is critical for axon degeneration but dispensable for glial TLR signaling. Conversely, the MAP3K Ask1 is required for glial TLR signaling, but not axon degeneration. These findings align well with the independent companion manuscript from the DiAntonio lab [34]. Together, these studies expand the repertoire of TLR signal transduction mechanisms to include deployment of the dSARM NADase for Ask1 activation. Here we discuss the dichotomous functions of dSARM in developmental and degenerative settings.

### An absolute requirement for the NADase activity of dSARM in axon degeneration *in vivo*

We found no difference in the protection afforded to either ORN or wing axons by deletion of *dSARM* or mutation of a key glutamic acid residue in dSARM’s active site (E1170A; [14,34]. Thus, the NAD^+^ hydrolase activity is strictly required for axon degeneration in these two *in vivo* paradigms. These results differ from a recently published study [27] that independently generated a *dSARM*^*E1170A*^ knock-in allele. These authors found that *dSARM*^*E1170A*^ mutant clones exhibit a weaker phenotype than *dSARM* nulls. In a wing axotomy assay, these *dSARM*^*E1170A*^ mutant clones provided only 50% protection of severed axons at 7 DPI relative to full protection in *dSARM* nulls [27,37]. This discrepancy may stem from distinct CRISPR strategies or, alternatively, differences in genetic background. To validate our approach, we both sequenced the entire *dSARM* locus in this background and demonstrated that recombination of the wild-type sequence into the founder chromosome fully rescues viability and fertility. The finding of an essential requirement for dSARM’s NAD^+^ hydrolase activity in axon degeneration in Drosophila is also in excellent agreement with data from mammalian models [14,45].

Unexpectedly, the behavior of the *dSARM*^*TIR*^ allele differed between ORN and wing sensory axon paradigms. In uninjured ORN axons, *dSARM*^*TIR*^ mutant clones exhibit spontaneous degeneration over the course of days arguing that SAM-mediated multimerization is not essential for NAD^+^ hydrolysis in the absence of the ARM domain. We propose that free TIR monomers have low-level constitutive activity leading to NAD^+^ loss, metabolic failure, and cellular demise. Consistent with unregulated activity of isolated TIR domains, the lethal phase of homozygous *dSARM*^*TIR*^ animals is significantly earlier than that of *dSARM*^*KO*^ homozygotes: early L1 for *dSARM*^*TIR*^, wandering L3 for *dSARM*^*KO*^. Surprisingly, *dSARM*^*TIR*^ mutant ORN clones not only exhibited slow, injury-independent degeneration, but were also capable of timely injury-induced degeneration. We suggest that *dSARM*^*TIR*^ clones are primed to degenerate given ongoing TIR domain activity, and that in this case, TIR monomers can support rapid axon destruction following axotomy. In contrast, *dSARM*^*TIR*^ behaved equivalently to the null allele in wing sensory axons. We did not see evidence of spontaneous axon degeneration in these neurons and mutant clones were fully protected for at least 7 days following axotomy.

It is possible that this difference reveals an underlying differential genetic sensitivity to NADase activity in these two neuronal populations. However, we hypothesize that the differential behavior of this allele may reflect a difference in timing of clone induction.

The generation of MARCM clones relies on enhancer-driven Flippase (FLP) activity that recombines Flippase Recombination Targets (FRTs) on a chromosomal arm [46]. ORN clones are induced by *ey-FLP* in the eye-antennal imaginal disc: *ey* activity starts in the eye-disc primordium (stage 15 embryo) and is maintained until the late third instar larvae [47]. In contrast, wing sensory neuron clones are induced by *ase-FLP* activity in the wing imaginal disc [37]. The activity of *ase* initiates in sensory organ precursors, which develop in third instar larvae and the first 10 h after puparium formation [48]. Thus, a likely explanation for the differential *dSARM*^*TIR*^ observation is that ORN clones are generated significantly earlier relative to wing sensory neuron clones, which might result in relatively higher levels of *dSARM*^*TIR*^ in adult neuron clones.

### The NADase activity of dSARM promotes TLR signal transduction

We uncovered a differential requirement for dSARM’s SAM domain in injury-induced degeneration and glial TLR signaling. dSARM/SARM1 assembles into an octamer via SAM-SAM mediated interactions [17–19], which is required for axon degeneration [12,18,20]. We generated a *dSARM* allele lacking only the SAM domains and found that it behaves as a null in axon degeneration. Thus, SAM domain-mediated dSARM multimerization is essential for axon degeneration *in vivo*. In contrast, *dSARM*^*ARM-TIR*^ homozygotes display normal glial TLR signaling, demonstrating that the SAM domains are not required in this context. We propose that in TLR signal transduction, dSARM’s TIR domains heterodimerize with TIR domains on TLR receptors. In this model, TIR-TIR interactions between TLRs and dSARM support the NADase activity of dSARM, which is consistent with the finding that catalytically inactive dSARM variants retain the TIR-TIR interactions leading to NADase activation (this work; [49]. These findings raise the possibility that dSARM exists in at least two distinct signaling complexes: a dSARM homomultimer that drives pathological axon degeneration and a dSARM-TLR heteromultimer that promotes signal transduction. It is alternatively possible that TLR activation drives dSARM homodimerization and NADase activity. It will be interesting to determine whether there are distinct pools of dSARM dedicated for each signaling state. While dSARM’s SAM domains are dispensable for glial TLR signaling, they must be required for other developmental functions of dSARM since *dSARM*^*ARM-TIR*^ homozygotes die at the L3/pupal transition. The SAM domains of TIR-1/SARM1 are proposed to regulate its synaptic localization in C. elegans [16], suggesting that dSARM’s SAM domains may likewise promote synaptic functions in motorneurons [30].

Evidence from multiple groups now argues for a dSARM/SARM1-Ask1 signaling cassette (this work; [16,27,34]. We do not yet know how dSARM activates Ask1, but we demonstrate that dSARM’s NADase activity is required for activation of Ask1 in glia. Ask1 orthologs are widely implicated in ROS-mediated signaling [50,51]. Specifically, they are ROS-activated by Thioredoxin (Trx), the first identified Ask1-binding protein and its major cellular inhibitor [42,52]. ROS signaling activates Ask1 because local oxidation relieves Trx-mediated repression and drives Ask1 activation [42,53]. Ask1 proteins lacking the Trx-binding domains (Ask1ΔN) can be constitutively active [42]. Indeed, we found that glial expression of Ask1ΔN activates TLR signaling, arguing that Trx may contribute to Ask1 activation in this context. It is conceivable that NAD^+^ hydrolysis mediated by dSARM could interfere with the maintenance of a reduced Trx pool, thus promoting activation of Ask1. Taken together with the companion study [34], these findings indicate that activation of the dSARM NAD^+^ hydrolase does not necessarily drive irreversible axon destruction, but rather that NAD^+^ hydrolysis is deployed for signaling in both neurons and glia.

## Methods

### Drosophila stocks

Drosophila melanogaster stocks were raised on standard molasses formulation. Both male and female flies were included in all analyses. Sex was not considered since phenotypes were generally evenly distributed, suggesting no detectable sex contribution. The following stocks were used: OregonR (OR; wild type), vasa>cas9 (BDSC, #55821), MKRS, hs-FLP / TM6B, Cre, Tb (BDSC, #1501), ey-flp, UAS-mCD8::GFP [11], OR22a-Gal4, UAS-mCD8::GFP [11], dSARM^896^ [11], FRT2A, 82B (BDSC, #8218), Wrapper932i-Gal4DBD, Nrv2-VP16AD (Ctxglia-split; [39], Wrapper932i-Gal4DBD, Nrv2-VP16AD, UAS-mCD8::mCherry (Ctxglia-split>UAS-mCD8::mCherry; [39]), dSARM RNAi #105369 (VDRC, #105369), Ask1 RNAi #35331 (BDSC, #35331), UAS-LacZ, UAS-Ask1-Flag (Kyoto DGRC, #109845), and UAS-Ask1ΔN (Kyoto DGRC, #109846).

### CRISPR/Cas9 generation of *dSARM* attP KO

The *dSARM attP KO* was designed following published methods [31,54]. Nucleotides 3L:8064263..8064447 of the endogenous *dSARM* gene were replaced with an attP and loxP site through homology-directed repair using a donor template. The attP site facilitates the knock-in of *dSARM* domain alleles. The pHD-DsRed-w+ (Addgene, #80898) was used to generate the donor template for homology-directed repair. Briefly, ~1 kb targeting homology arms flanking the domain-encoding exons of the *dSARM* gene, the pHD-DsRed-w+ backbone, and attP-loxP-DsRed-loxP insert were generated by PCR. These four products were then assembled using NEB HiFi DNA assembly (NEB, # E5520S). A dSARM targeting chimeric RNA (chiRNA) was cloned into the pU6-BbsI-chiRNA (DGRC, #1362) vector using site-directed mutagenesis for the following target sequence: 5’- AAGGTTGTAAGGGTCCCCAGGGG -3’ (NEB, # E0554S). Both the chiRNA and the pHD-DsRed-w+ plasmids were injected (BestGene) into vasa>Cas9 embryos (BDSC, #55821) to produce *dSARM* deletions. Successful events were screened for by the presence of DsRed positive eyes. The allele was named *dSARM attP KO*. Genomic PCR bands corresponding to primers flanking the targeted exons confirmed the deletion of the domain encoding exons of *dSARM attP KO*. The DsRed selection marker was removed by crossing to flies expressing Cre recombinase (MKRS, hs-FLP/TM6B, Cre, Tb) (BDSC, #1501).

### phiC31-mediated recombination of *dSARM* rescue and mutants

As a control, an integration plasmid (pGE-attB-GMR) [33] containing wild-type dSARM nucleotides 3L:8064263–8064447 were injected (Rainbow Transgenic Flies) into *dSARM attP KO* embryos and integrated at the *dSARM* locus through phiC31-mediated recombination. Successful events were screened for by the presence of red eyes, and the red (w+) containing cassette was removed by Cre recombinase. The resulting *dSARM rescue* flies are homozygous-viable, fertile, and display no apparent phenotypes. To generate dSARM E1170A, we mutagenized the dSARM rescue plasmid, converting the NADase catalytic glutamic acid residue (GAA) to an alanine (GCC) (nucleotides 3L:8105127–8105129). To generate dSARM ARM-SAM, we mutagenized the dSARM rescue plasmid to remove the nucleotides 3L:8104756–8105455 corresponding to the TIR domain. To generate dSARM TIR, we mutagenized the dSARM rescue plasmid to remove the nucleotides 3L:8103445–8104722 corresponding to the ARM and SAM domains. To generate dSARM ARM-TIR, we mutagenized the dSARM rescue plasmid to remove the nucleotides 3L:8104303–8104722 corresponding to the SAM domains. To generate dSARM SAM, we mutagenized the dSARM ARM-SAM plasmid to remove the nucleotides 3L:8103445–8104299 corresponding to the ARM domain.

### CRISPR/Cas9 generation of Ask1Δ6

The Ask1Δ6 mutant was designed following published methods [31,54]. The endogenous Ask1 gene (nucelotides 3R:19879704–19886056) was replaced with an attP site through homology-directed repair. The pHD-DsRed-w+ (Addgene, #80898) was used to generate the donor template for homology-directed repair. Briefly, ~1 kb targeting homology arms flanking the 5’ and 3’ UTRs of the Ask1 gene, the pHD-DsRed-w+ backbone, and attP-loxP-DsRed-loxP insert were generated by PCR. These four products were assembled using NEB HiFi DNA assembly (NEB, # E5520S). An Ask1 targeting chimeric RNA (chiRNA) was cloned into the pU6-BbsI-chiRNA (DGRC, #1362) vector using site-directed mutagenesis for the following CRISPR target sequence: 5’- GTATTTCTTGTTAACTGGAAAGG -3’ (NEB, # E0554S). The chiRNA and the pHD-DsRed-w+ plasmids were injected into vasa>Cas9 embryos (BDSC, #55821). Successful events were screened for by the presence of DsRed-positive eyes. The allele was named Ask1Δ6 for the 6350 bp deletion. Genomic PCR bands corresponding to primers flanking the Ask1 gene confirmed the absence of Ask1Δ6. This approach resulted in the deletion of the entire Ask1 gene (upstream flanking sequence: 5′- GTATATTGCTGGTAGCTGTG-3′, downstream flanking sequence: 5′- GAAAGGATTTATAGCTTCTG-3′). The dsRed selection marker was removed by crossing to flies expressing Cre recombinase (MKRS, hs-FLP / TM6B, Cre, Tb).

### Antennal injury protocol

We induced antennal injury using a modification of a previously described protocol [11,55]. Animals were aged 1, 7, or 30 days at 25°C. Only the right antennal segment was surgically ablated using forceps. Injured flies were aged at 25°C for the indicated time (12 hours, 1, 2, 7, or 30 days). Number of axons was scored as previously described [8].

### Wing injury protocol

We performed wing injuries using a modification of a previously described protocol [38]. One wing per anesthetized fly was cut approximately in the middle. The distal, cut-off part of the wing was mounted in Halocarbon Oil 27 on a microscopy slide, covered with a coverslip, and immediately used to count the amount of cut-off cell bodies (as readout for the number of injured axons) under an epifluorescence microscope. Flies were returned to individual vials. At 7 DPI, wings were mounted onto a slide, and imaged with a spinning disk microscope to assess for intact or degenerated injured axons, as well as the remaining uninjured intact neurons.

### Adult immunohistochemistry

Adult heads were fixed with 4% formaldehyde (Thermo Scientific, #28906) in 1x PBS and 0.1% Triton X-100 (PTX) for 30 min and washed 5 times, 2 minutes each with PTX before dissection. Brains were dissected in PTX. Dissected brains were subsequently fixed in 4% formaldehyde in PTX for 10 min and washed 5 times, 2 minutes each with PTX. Primary antibody anti-chicken GFP (Aves Labs, #GFP-1020) was used at 1:500 in PTX and rocked overnight at 4°C. Brains were washed 3 times, 10 minutes each with PTX. Alexa Fluor 488 goat anti-chicken (Invitrogen, #A11039) was used at 1:100 in PTX and rocked overnight at 4°C. Following antibody incubation brains were washed 3 times, 10 minutes each with PTX. Brains were mounted in ProLong Gold with DAPI (Invitrogen, #P36935).

### Confocal microscopy

Fluorescent 16-bit images were acquired on an upright Zeiss LSM 800 (Carl Zeiss) using Zen software (Carl Zeiss). The following objectives were used: 20x Plan-Apochromat (0.8 NA) air objective (larval experiments) or 40x Fluar (1.3 NA) oil immersion objective (adult experiments).

### Axonal intensity and axonal debris measurements

Maximum intensity Z-projections were analyzed in Image J/Fiji. Axons were isolated from the image (the glomeruli were not analyzed) one side at a time. A threshold using the default settings was applied to highlight the axons. This threshold was then used to create a selection. From this selection, the average intensity was measured. For axonal debris, we started with a maximum intensity Z-projection in Image J/Fiji. We generated a region of interest on the injured side by applying a threshold to detect the fluorescent signal and generated a selection. From this selection, we used the analyze particles function to get total fluorescent area.

### qRT-PCR

Fifty first or second instar larvae of each genotype were collected in an 0.6mL Eppendorf tube in nuclease-free water on ice. The larvae were homogenized in the 0.6 mL tube with a pestle. RNA was isolated using the RNeasy plus kit (Qiagen). cDNA was synthesized from 1 mg total RNA using the iScript kit (BioRad) and the cDNA was diluted 1:5. qRT-PCR was performed using Taqman probes from the Life Technologies database: Rpll140 (Dm02134594_g1) and dSARM (Dm01840803_m1). These were utilized in a StepOnePlus Real-Time PCR system (Applied Scientific/ Thermo Scientific). Rpll140 was used as the reference gene for the DDC_T_ comparison protocol to quantify relative changes in gene expression, all in technical triplicate. No-reverse transcriptase controls were conducted to confirm the purity of each cDNA sample, and lack of gDNA contamination in the qRT-PCR. We performed 4 biological replicates per mRNA and 3 technical replicates per biological replicate.

### Larval immunohistochemistry

Rapid dissections were performed in PBS to expose 3^rd^ instar larval (L3) CNS. Extracted brains were fixed in 4% paraformaldehyde (PFA) in PBS for 20 minutes. Fixed brains were washed 3x 5min in PBS and blocked in PTN (1X PBS, 0.1% Triton X-100, 1% NGS) for 30 minutes and incubated in primary antibody for two overnights at 4°C. Following incubation in primary antibody, brain samples were washed 3x 5min in PTN and incubated in secondary antibody for 2 hours at room temperature. After incubation in secondary antibody, samples were washed 3x 5min in PBS and mounted in Prolong Gold antifade reagent with DAPI (Thermo Fisher). Mounted samples were solidified at room temperature overnight before image acquisition. The following primary and secondary antibodies were used: rabbit anti-cleaved Drosophila Dcp-1 (Asp216) (Cell Signaling Technology; at 1:100), goat anti-rabbit IgG (Alexa-conjugated fluor 568) (Thermo Fisher; at 1:300), and mouse anti-Draper (5D14; DSHB; at 1:300).

### Larval Dcp-1 counting

Acquired confocal images of 3^rd^ instar larval (L3) CNS were analyzed using Imaris Microscopy Image Analysis Software (Oxford Instruments). To reduce background noise, a background subtraction filter (2.5 μm in width) under Image Processing function was first applied to the image. After image processing, contour of both brain lobes was traced across top to bottom slices using the surface function to define a region of interest. Each region of interest recapitulates the entirety of an individual brain lobe and measures its volume. Within each region of interest, Spots Detection function (at 2.0 XY diameter and 2.0 Z-axis diameter, with Quality filter) was deployed to automatically label individual Dcp-1 puncta based on a local contrast screening of relative intensity and to generate a total count in each brain lobe. Total Dcp-1 puncta count is then divided by individual brain lobe volume to yield normalized Dcp-1 puncta count (#/μm^3^).

### Draper quantification

Larval brains were imaged and processed as previously described [29]. Briefly, control and mutant genotypes were imaged using identical acquisition settings. Z-stacks were acquired with optimized confocal settings to ensure that oversaturation did not occur. Average intensity was quantified from 3 um thick z-projections using Fiji (National Institutes of Health). For cortex glia knockdown experiments, an ROI was defined by mCherry labeling cortex glia expressing UAS-mCD8::mCherry. For analysis of Ask1Δ6, we quantified Draper intensity around the neuropil corresponding to cortex glial processes.

### Quantification and statistical analysis

All statistical analyses were performed and graphs generated using Prism 9 (GraphPad Software). In all box and whisker plots, the whiskers represent minimum and maximum data points, and the line within the box depicts median values. In all bar graphs, error bars are mean ± SEM. All pairwise sample comparisons were performed using a Mann-Whitney test. For data in a group of three or more, a Kruskal-Wallis test was used to compare each sample with other samples, and a Dunn’s multiple comparison test was subsequently performed. A two-way ANOVA followed by a Dunnett’s multiple comparisons test was performed on adult ORN axon degeneration data (Figs 2I, 2Q and 4J). In all figures, p-values for statistical tests are as follow: n.s., not significant; *, P<0.05; **, P<0.01; ***, P<0.001; ****, P<0.0001. n values are found on each graph and/or figure legends.

## Supporting information

S1 Fig *dSARM*^*TIR*^ homozygous mutant OR22a axons exhibit timely degeneration following injury. (A,B) Representative z-projections of OR22a ORNs of the indicated genotypes labeled with anti-GFP at 12 Hours post injury (HPI). (C) Normalized mean axon intensity at 12 HPI: dSARM rescue: 0.80 (n = 24), dSARM^TIR^: 0.56 (n = 22).
(PDF)

S2 Fig Ask1 is not required for injury-induced axon degeneration. (A,B) Representative z-projections of OR22a ORNs of the indicated genotypes labeled with anti-GFP at 7 DPE, 1 DPI. (C) Normalized mean axon intensity at 1 DPI: wild type (FRT2A): 0.91, Ask1Δ6: 0.86, (D-F) Representative z-projections of OR22a ORNs of the indicated genotypes labeled with anti-GFP at 7 DPE, 1 DPI. (G) Normalized mean axon intensity at 1 DPI: wild type (FRT2A): 0.85 (n = 35); OR22a-Gal4, UAS-Ask1-Flag: 0.76 (n = 24); OR22a-Gal4, UAS-Ask1ΔN: 0.61 (n = 26). n.s., not significant.
(PDF)

S3 Fig *dSARM* alleles do not have dominant effects on Dcp-1 levels in the L3 brain. (A-G) Representative z-projections of brain lobes of the indicated genotypes labeled with anti-Dcp-1. (H) Quantification of the number of Dcp-1 puncta normalized to brain lobe volume. Mean number of Dcp-1 puncta/brain lobe volume: dSARM^Rescue^: 1.56x10^-4^, dSARM^KO^: 1.45x10^-4^, dSARM^ARM-SAM^: 1.53x10^-4^, dSARM^ARM-TIR^: 1.36x10^-4^, dSARM^E1170A^: 1.72x10^-4^, dSARM^SAM^: 1.60x10^-4^, and dSARM^TIR^: 1.89x10^-4^. n values can be found on the graph. n.s., not significant.
(PDF)

S1 Data Underlying numerical data. (PDF)
