# Supplementary figures and images for: Divergent signaling requirements of dSARM in injury-induced degeneration and developmental glial phagocytosis
Source: PLoS Genet. 2022 Jun 23;18(6):e1010257. doi: 10.1371/journal.pgen.1010257 (PMC9223396; doi:10.1371/journal.pgen.1010257)

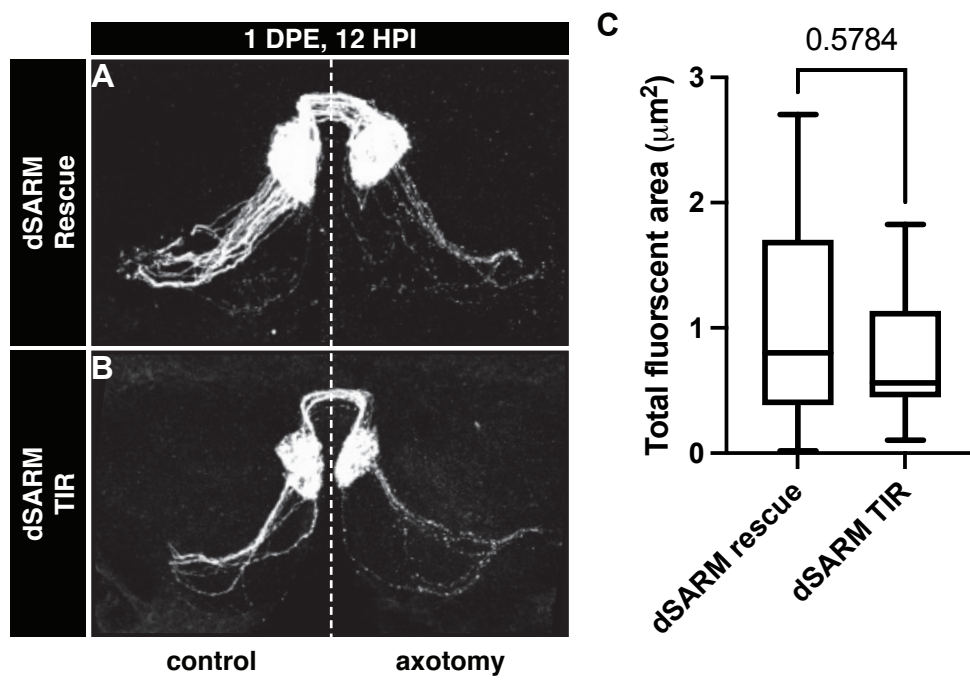

Figure S1

Supplement: S1 Fig — (A,B) Representative z-projections of OR22a ORNs of the indicated genotypes labeled with anti-GFP at 12 Hours post injury (HPI). (C) Normalized mean axon intensity at 12 HPI: dSARM rescue: 0.80 (n = 24), dSARMTIR: 0.56 (n = 22). (PDF) [file pgen.1010257.s001.pdf]

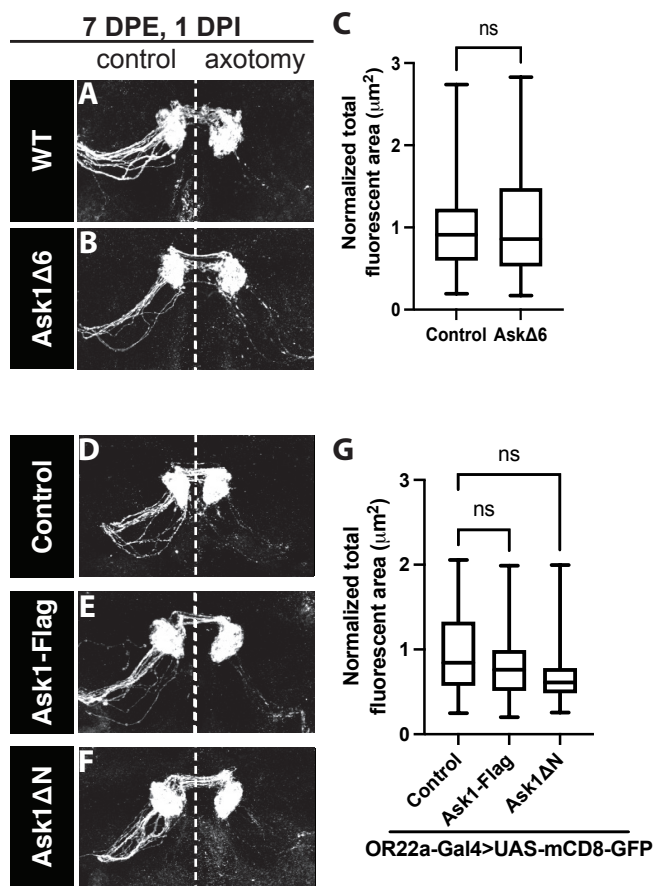

**Figure S2**

Supplement: S2 Fig — (A,B) Representative z-projections of OR22a ORNs of the indicated genotypes labeled with anti-GFP at 7 DPE, 1 DPI. (C) Normalized mean axon intensity at 1 DPI: wild type (FRT2A): 0.91, Ask1Δ6: 0.86, (D-F) Representative z-projections of OR22a ORNs of the indicated genotypes labeled with anti-GFP at 7 DPE, 1 DPI. (G) Normalized mean axon intensity at 1 DPI: wild type (FRT2A): 0.85 (n = 35); OR22a-Gal4, UAS-Ask1-Flag: 0.76 (n = 24); OR22a-Gal4, UAS-Ask1ΔN: 0.61 (n = 26). n.s., not significant. (PDF) [file pgen.1010257.s002.pdf]

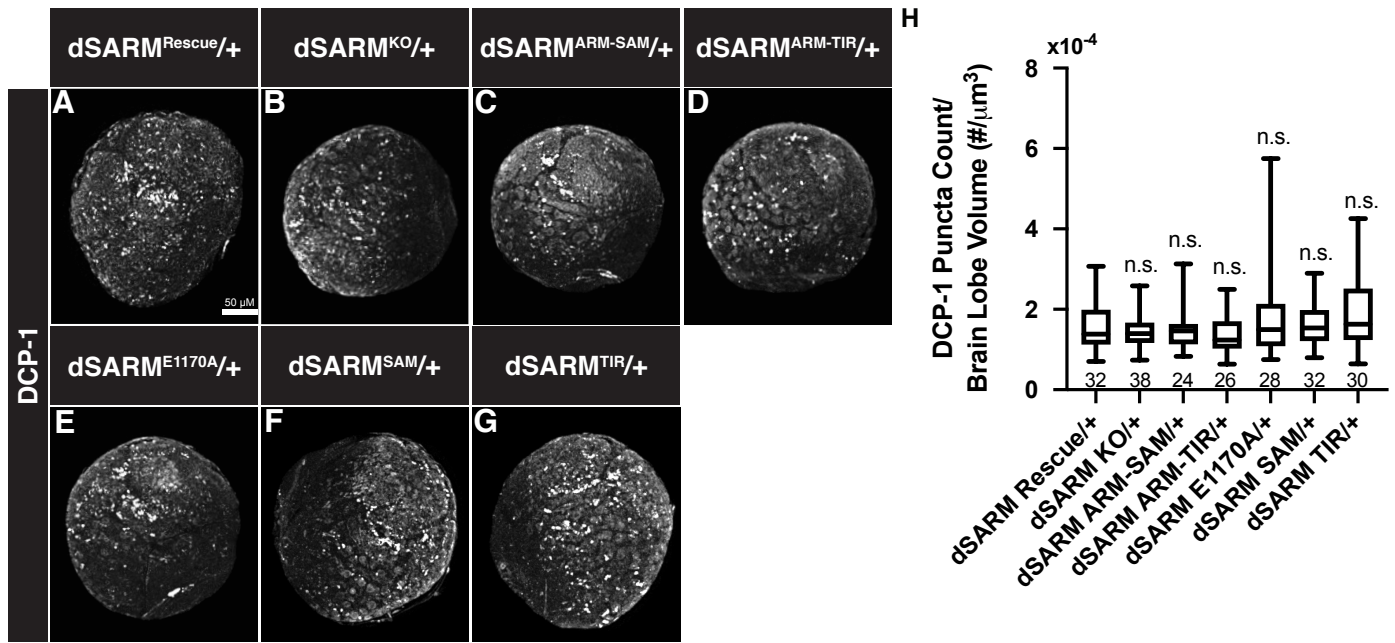

Figure S3

Supplement: S3 Fig — (A-G) Representative z-projections of brain lobes of the indicated genotypes labeled with anti-Dcp-1. (H) Quantification of the number of Dcp-1 puncta normalized to brain lobe volume. Mean number of Dcp-1 puncta/brain lobe volume: dSARMRescue: 1.56x10^-4^, dSARMKO: 1.45x10^-4^, dSARMARM-SAM: 1.53x10^-4^, dSARMARM-TIR: 1.36x10^-4^, dSARME1170A: 1.72x10^-4^, dSARMSAM: 1.60x10^-4^, and dSARMTIR: 1.89x10^-4^. n values can be found on the graph. n.s., not significant. (PDF) [file pgen.1010257.s003.pdf]
