# Supplementary material for: Divergent signaling requirements of dSARM in injury-induced degeneration and developmental glial phagocytosis
Source: PLoS Genet. 2022 Jun 23;18(6):e1010257. doi: 10.1371/journal.pgen.1010257 (PMC9223396; doi:10.1371/journal.pgen.1010257)
Supplement: S1 Data — (PDF) [file pgen.1010257.s004.pdf]

sample	target gene	housekeeping	delta ct	mean delta ct	delta delta ct	fold change normalized to control
	dSARM	Rpll140				2^-ddct
OR #1	25.4698677	24.1800842	1.28978348	1.72683652	-0.437053	1.35383606
OR #1	25.1873016	23.9849586	1.20234299		-0.5244935	1.43842852
OR #1	25.5297775	22.8413944	2.6883831		0.96154658	0.51350614
dSARM rescue #1	23.3076973	23.2670097	0.04068756		-1.686149	3.21796573
dSARM rescue #1	22.7559452	23.4741917	-0.7182465		-2.445083	5.44556968
dSARM rescue #1	22.7361946	22.8886337	-0.1524391		-1.8792756	3.678903
OR #2	23.8722916	24.1204662	-0.2481747	0.00327365	-0.2514483	1.19040155
OR #2	23.7340145	23.210659	0.52335548		0.52008184	0.69733228
OR #2	23.5336418	23.7990017	-0.2653599		-0.2686335	1.20466627
dSARM rescue #2	22.8863029	22.8446465	0.04165649		0.03838285	0.97374583
dSARM rescue #2	22.8839817	23.0571098	-0.1731281		-0.1764018	1.13006188
dSARM rescue #2	23.2049923	22.9500122	0.25498009		0.25170644	0.83990238
OR #3	24.8095703	23.8630047	0.94656563	0.86893272	0.0776329	0.94761116
OR #3	24.6082993	23.4894886	1.11881065		0.24987793	0.84096757
OR #3	23.565649	23.0242271	0.54142189		-0.3275108	1.25484645
dSARM rescue #3	24.2911606	24.2934093	-0.0022488		-0.8711815	1.82916027
dSARM rescue #3	24.1789207	24.526329	-0.3474083		-1.216341	2.32356663
dSARM rescue #3	23.5329971	24.2670536	-0.7340565		-1.6029892	3.03772063
OR #4	23.6874199	23.6102009	0.07721901	1.06587156	-0.9886525	1.98433079
OR #4	23.9619122	22.3442879	1.61762428		0.55175273	0.68219083
OR #4	23.3996811	21.8969097	1.50277138		0.43689982	0.73872033
dSARM rescue #4	23.6023388	23.2333336	0.3690052		-0.6968664	1.62098007
dSARM rescue #4	22.9124775	22.5882034	0.32427406		-0.7415975	1.67202625
dSARM rescue #4	22.5090637	23.2979259	-0.7888622		-1.8547338	3.61685005

averge fold change (normalized to control)	Notes
1.10192357	
4.11414614	
1.03080003	
0.9812367	
1.01447506	
2.39681585	
1.13508065	
2.30328545	

OR	dSARM
1.10192357	4.11414614
1.03080003	0.9812367
1.01447506	2.39681585
1.13508065	2.30328545

sample	target gene dSARM	housekeeping Rpll140	delta ct	mean delta ct	delta delta ct	fold change normalized to control 2^-ddct
OR #1	25.7226906	24.1615639	1.56112671	1.65977478	-0.0986481	1.07076959
OR #1	25.5705109	24.3351669	1.23534393		-0.4244308	1.34204295
OR #1	25.8038483	23.6209946	2.1828537		0.52307892	0.69588513
dSARM E642A #1	24.57901	22.8572445	1.72176552		0.06199074	0.95794137
dSARM E642A #1	24.6487064	23.515728	1.13297844		-0.5267963	1.44072635
dSARM E642A #1	24.5076103	23.5888138	0.91879654		-0.7409782	1.67130871
OR #2	24.7282619	23.811615	0.91664696	0.9255422	-0.0088952	1.00618476
OR #2	24.6813335	23.6142941	1.06703949		0.14149729	0.90657778
OR #2	24.5868778	23.7939377	0.79294014		-0.1326021	1.09626916
dSARM E642A #2	24.9097061	23.6447544	1.26495171		0.33940951	0.79036474
dSARM E642A #2	24.8170662	23.6337509	1.18331528		0.25777308	0.83637794
dSARM E642A #2	24.781065	23.7574062	1.02365875		0.09811656	0.93425186
OR #3	25.6588898	23.0400639	2.61882591	2.357687	0.26113892	0.83442893
OR #3	25.5689278	23.1350632	2.43386459		0.0761776	0.94856754
OR #3	25.3324032	23.3120327	2.02037048		-0.3373165	1.26340441
dSARM E642A #3	25.6920891	23.7468433	1.94524574		-0.4124413	1.33093605
dSARM E642A #3	25.4219589	23.6181087	1.80385017		-0.5538368	1.46798459
dSARM E642A #3	25.5287361	23.7356319	1.79310417		-0.5645828	1.47895979
OR #4	24.6653194	21.963438	2.70188141	2.4557546	0.24612681	0.84315699
OR #4	24.8212566	22.3954506	2.42580605		-0.0299486	1.02097572
OR #4	24.6994057	22.4598293	2.23957634		-0.2161783	1.16165226
dSARM E642A #4	24.8116875	23.3135662	1.49812126		-0.9576333	1.94212133
dSARM E642A #4	23.7303772	22.9631519	0.76722527		-1.6885293	3.22327959
dSARM E642A #4	23.9022293	23.3902645	0.5119648		-1.9437898	3.84714925

averge fold change (normalized to control)	Notes
1.03623256	
1.35665881	
1.00301056	
0.85366485	
1.01546696	
1.42596014	
1.00859499	
3.00418339	

sample	target gene	housekeeping	delta ct	mean delta ct	delta delta ct	fold change normalized to control
	dSARM	Rpll140				2^-ddct
OR #1	26.1632805	23.7002106	2.46306992	2.28240649	0.18066343	0.88229718
OR #1	26.0100327	24.1653347	1.84469795		-0.4377085	1.35445131
OR #1	25.9089642	23.3695126	2.5394516		0.25704511	0.83680008
dSARM^TIR#1	25.3255596	21.801033	3.5245266		1.24212011	0.42275095
dSARM^TIR#1	24.9977493	22.4032288	2.59452057		0.31211408	0.8054606
dSARM^TIR#1	24.7939396	22.1395855	2.6543541		0.37194761	0.77273861
OR #2	25.5855789	23.3303928	2.25518608	2.20720673	0.04797935	0.96729017
OR #2	25.5070515	23.2113323	2.29571915		0.08851242	0.940492
OR #2	25.450901	23.3801861	2.07071495		-0.1364918	1.09922885
dSARM^TIR#2	25.6264839	22.5228596	3.10362434		0.89641762	0.53721905
dSARM^TIR#2	25.5737953	22.3999672	3.17382813		0.9666214	0.511703
dSARM^TIR#2	25.6054249	21.8568974	3.74852753		1.5413208	0.34357077
OR #3	25.971302	23.0540085	2.91729355	3.09980774	-0.1825142	1.13485989
OR #3	26.2687111	22.3435974	3.92511368		0.82530594	0.56436251
OR #3	25.8965569	23.4395409	2.45701599		-0.6427917	1.56134759
dSARM^TIR#3	24.0661068	23.338995	0.72711182		-2.3726959	5.17908028
dSARM^TIR#3	24.1394081	22.8709927	1.26841545		-1.8313923	3.55880353
dSARM^TIR#3	23.8533497	23.0397873	0.81356239		-2.2862453	4.87784984
OR #4	25.1446304	22.6209412	2.52368927	2.49277178	0.03091749	0.97879763
OR #4	25.1716251	22.2890282	2.88259697		0.38982519	0.76322208
OR #4	24.9794254	22.9073963	2.07202911		-0.4207427	1.33861647
dSARM^TIR#4	26.1516438	21.7676849	4.38395882		1.89118703	0.26958516
dSARM^TIR#4	26.2761288	20.7907696	5.48535919		2.99258741	0.1256439
dSARM^TIR#4	25.8772488	20.8360825	5.04116631		2.54839452	0.17094516

averge fold change (normalized to control)	Notes
1.02451619	
0.66698339	
1.00233701	
0.46416427	
1.08685666	
4.53857788	
1.02687873	
0.18872474	

sample	target gene	housekeeping	delta ct	mean delta ct	delta delta ct	fold change normalized to control
	dSARM	Rpll140				2^-ddct
OR #5	23.7649021	23.4753094	0.28959274	0.43139267	-0.1417999	1.10328073
OR #5	23.5886307	23.3861217	0.20250893		-0.2288837	1.17192784
OR #5	23.7312527	22.9291763	0.80207634		0.37068367	0.7734159
dSARM KO#1	29.841114	22.764349	7.07676506		6.64537239	0.0099895
dSARM KO#1	29.7629833	22.8696194	6.89336395		6.46197128	0.01134365
dSARM KO#1	30.2348881	22.9965744	7.23831367		6.80692101	0.00893126
OR #6	24.6259804	23.7646961	0.86128426	0.82842445	0.0328598	0.97748075
OR #6	24.7020473	23.4775333	1.22451401		0.39608955	0.75991526
OR #6	24.3792496	23.9797745	0.3994751		-0.4289494	1.34625281
dSARM KO#2	29.753231	24.2178917	5.53533936		4.7069149	0.0382893
dSARM KO#2	29.5148888	24.1403866	5.37450218		4.54607773	0.04280497
dSARM KO#2	29.7439365	24.2112503	5.53268623		4.70426178	0.03835978
OR #7	24.1146431	23.4496307	0.66501236	0.36087799	0.30413437	0.80992804
OR #7	23.7603836	23.3322315	0.42815208		0.06727409	0.95443967
OR #7	23.2530689	23.2635994	-0.0105305		-0.3714085	1.29361513
dSARM KO#3	31.0027008	24.3080864	6.69461441		6.33373642	0.01239811
dSARM KO#3	31.2202568	24.1562824	7.06397438		6.70309639	0.00959769
dSARM KO#3	30.5324421	24.18783	6.34461212		5.98373413	0.01580216
OR #8	25.9336987	27.2987881	-1.3650894	0.22919146	-1.5942809	3.01943974
OR #8	25.8973618	24.8491955	1.04816628		0.81897481	0.5668446
OR #8	25.9501534	24.9456558	1.00449753		0.77530607	0.58426466
dSARM KO#4	32.5116425	24.6523895	7.85925293		7.63006147	0.00504804
dSARM KO#4	32.4473457	24.1644688	8.28287697		8.05368551	0.00376356
dSARM KO#4	33.4644051	24.4672527	8.99715233		8.76796087	0.00229393

averge fold change (normalized to control)	Notes
1.01620816	
0.01008813	
1.02788294	
0.03981802	
1.01932761	
0.01259932	
1.390183	
0.00370184	

sample	target gene dSARM	housekeeping Rpll140	delta ct	mean delta ct	delta delta ct	fold change normalized to control 2^-ddct
OR #1	22.8536415	24.0800877	-1.2264462	-1.2529882	0.02654203	0.98177067
OR #1	23.155674	24.1794071	-1.0237331		0.22925504	0.85307528
OR #1	22.5640755	24.0728607	-1.5087852		-0.2557971	1.19399523
dSARM^SAM #1	24.8204441	24.6402607	0.18018341		1.43317159	0.3703159
dSARM^SAM #1	24.5882473	24.8236942	-0.2354469		1.01754125	0.49395748
dSARM^SAM #1	23.7490139	24.7787228	-1.0297089		0.22327932	0.85661609
OR #2	23.9990711	24.6032581	-0.604187	-0.2980302	-0.3061568	1.23640963
OR #2	24.3663483	24.4518394	-0.0854912		0.21253904	0.86301705
OR #2	24.5341129	24.7385254	-0.2044125		0.09361776	0.93716972
dSARM^SAM #2	24.6105728	25.0846424	-0.4740696		-0.1760394	1.12977805
dSARM^SAM #2	24.1791	24.6694622	-0.4903622		-0.1923319	1.14260912
dSARM^SAM #2	24.421875	24.3715572	0.05031776		0.34834798	0.78548303
OR #3	23.4108143	24.1870213	-0.776207	-1.1252785	0.3490715	0.78508921
OR #3	23.3818359	24.2583637	-0.8765278		0.24875069	0.84162491
OR #3	22.5023804	24.225481	-1.7231007		-0.5978222	1.51343025
dSARM^SAM #3	23.8992519	25.1511726	-1.2519207		-0.1266422	1.09174977
dSARM^SAM #3	24.0307178	24.5739574	-0.5432396		0.58203888	0.66801904
dSARM^SAM #3	23.6452522	24.6909599	-1.0457077		0.07957077	0.94633916
OR #4	25.8366737	25.5708656	0.26580811	-0.3542531	0.62006124	0.65064331
OR #4	25.3125362	25.3880177	-0.0754814		0.27877172	0.8242925
OR #4	24.2419701	25.4950562	-1.2530861		-0.898833	1.86455707
dSARM^SAM #4	24.2809448	25.3472729	-1.066328		-0.7120749	1.63815846
dSARM^SAM #4	23.8919678	24.7347813	-0.8428135		-0.4885604	1.4030441
dSARM^SAM #4	24.118906	24.918438	-0.7995319		-0.4452788	1.36157722

averge fold change (normalized to control)	Notes
1.00961372	
0.57362982	
1.0121988	
1.01929007	
1.04671479	
0.90203599	
1.1131643	
1.46759326	

sample	target gene dSARM	housekeeping Rpll140	delta ct	mean delta ct	delta delta ct	fold change normalized to control 2^-ddct
OR #1	23.5477066	22.874382	0.67332458	0.930041	-0.2567164	1.19475633
OR #1	23.6172485	22.4495373	1.16771126		0.23767026	0.84811378
OR #1	23.2459545	22.2968674	0.94908714		0.01904615	0.98688498
dSARM^ARM^SAM #1	23.9657631	23.3301926	0.63557053		-0.2944705	1.22643474
dSARM^ARM^SAM #1	23.6353931	23.6161804	0.01921272		-0.9108283	1.8801246
dSARM^ARM^SAM #1	24.3024883	23.7606277	0.54186058		-0.3881804	1.30874172
OR #2	24.3871441	22.9354801	1.45166397	1.6863308	-0.2346668	1.17663497
OR #2	24.6052132	22.632803	1.9724102		0.28607941	0.82012777
OR #2	24.5606575	22.9257393	1.63491821		-0.0514126	1.03627908
dSARM^ARM^SAM #2	26.2230301	24.8803806	1.34264946		-0.3436813	1.26899056
dSARM^ARM^SAM #2	25.9073296	24.8074169	1.09991264		-0.5864182	1.50151423
dSARM^ARM^SAM #2	26.2591038	24.7935352	1.46556854		-0.2207623	1.16534914
OR #3	23.8532295	22.6633778	1.18985176	0.86684227	0.32300949	0.79940057
OR #3	23.8546047	23.2724018	0.58220291		-0.2846394	1.21810573
OR #3	23.3160877	22.4876156	0.82847214		-0.0383701	1.02695298
dSARM^ARM^SAM #3	24.9632816	24.6913967	0.27188492		-0.5949574	1.51042793
dSARM^ARM^SAM #3	25.2608967	24.4844093	0.77648735		-0.0903549	1.06463206
dSARM^ARM^SAM #3	24.7628136	24.4163609	0.34645271		-0.5203896	1.4343425
OR #4	25.9041996	23.8478146	2.05638504	2.00232188	0.05406316	0.96321972
OR #4	25.844408	23.9212437	1.92316437		-0.0791575	1.05640096
OR #4	25.6296806	23.6022644	2.02741623		0.02509435	0.98275633
dSARM^ARM^SAM #4	24.854311	25.0105591	-0.1562481		-2.15857	4.46472084
dSARM^ARM^SAM #4	24.4570885	24.648756	-0.1916676		-2.1939894	4.57569039
dSARM^ARM^SAM #4	24.480608	24.8898029	-0.4091949		-2.4115168	5.32033403

averge fold change (normalized to control)	Notes
1.00991836	
1.47176702	
1.01101394	
1.31195131	
1.01481976	
1.3364675	
1.00079233	
4.78691508	

sample	target gene dSARM	housekeeping Rpll140	delta ct	mean delta ct	delta delta ct	fold change normalized to control 2^-ddct
OR #1	27.1151752	25.4380302	1.677145	1.68358103	-0.006436	1.00447108
OR #1	26.9914074	25.3823624	1.60904503		-0.074536	1.05302231
OR #1	26.8975792	25.1330261	1.76455307		0.08097204	0.94542044
dSARM^ARM^TIR#1	24.7029171	22.9969501	1.70596695		0.02238592	0.98460303
dSARM^ARM^TIR#1	24.6692677	23.2511787	1.41808891		-0.2654921	1.20204601
dSARM^ARM^TIR#1	24.2899895	23.1294193	1.16057014		-0.5230109	1.43695102
OR #2	24.5258121	23.1574917	1.36832047	1.48255475	-0.1142343	1.08240041
OR #2	24.7429085	22.9297295	1.81317902		0.33062426	0.79519232
OR #2	24.698122	23.4319572	1.26616478		-0.21639	1.16182274
dSARM^ARM^TIR#2	25.3030281	23.4861565	1.81687164		0.33431689	0.79315961
dSARM^ARM^TIR#2	25.3349228	23.4079018	1.92702103		0.44446627	0.73485613
dSARM^ARM^TIR#2	25.563673	23.3395824	2.22409058		0.74153582	0.5981023
OR #3	25.0283642	23.43901	1.58935418	1.44997354	0.13938065	0.90790884
OR #3	25.1837196	23.46579	1.71792964		0.2679561	0.8304953
OR #3	24.4923668	23.44973	1.04263679		-0.4073367	1.32623529
dSARM^ARM^TIR#3	24.4902554	23.7126999	0.77755547		-0.6724181	1.59374197
dSARM^ARM^TIR#3	24.6384602	23.5886459	1.04981422		-0.4001593	1.31965363
dSARM^ARM^TIR#3	24.3306465	23.660635	0.67001152		-0.779962	1.71708566
OR #4	25.9796505	24.4012299	1.57842064	1.36516953	0.21325111	0.86259119
OR #4	25.7971573	24.3286152	1.4685421		0.10337257	0.9308544
OR #4	25.3531418	24.3045959	1.04854584		-0.3166237	1.24541252
dSARM^ARM^TIR#4	25.4790077	23.6302586	1.84874916		0.48357964	0.71520085
dSARM^ARM^TIR#4	25.3162918	23.1069603	2.20933151		0.84416199	0.55703428
dSARM^ARM^TIR#4	25.2568722	23.4746799	1.78219223		0.41702271	0.74896868

averge fold change (normalized to control)	Notes
1.00097128	
1.20786669	
0.20689541	
1.01313849	
0.70870601	
-0.3044325	
1.02154647	
1.54349375	
0.52194728	
1.0129527	
0.6737346	
-0.3392181	
0.02129803	

	WT	KO	dSARM rescue	dSARM E1170A	dSARM ARM-SAM	dSARM TIR
	1.01620816	0.01008813	4.11414614	1.35665881	0.66698339	1.47176702
	1.02788294	0.03981802	0.9812367	0.85366485	0.46416427	1.31195131
	1.01932761	0.03981802	2.39681585	1.42596014	4.53857788	1.3364675
	1.390183	0.00370184	2.30328545	3.00418339	0.18872474	4.78691508
	1.10192357					
	1.03080003					
	1.01447506					
	1.13508065					
	1.03623256					
	1.00301056					
	1.01546696					
	1.00859499					
	1.02451619					
	1.00233701					
	1.08685666					
	1.02687873					
	1.00961372					
	1.0121988					
	1.04671479					
	1.1131643					
	1.00991836					
	1.01101394					
	1.01481976					
	1.00079233					
	1.00097128					
	1.01313849					
	1.02154647					
	1.0129527					
AVG	1.043093558	0.023356503	2.448871035	1.660116798	1.46461257	2.226775228
ST DEV	0.076602757	0.019186094	1.284652563	0.931628653	2.058661506	1.708206087
SEM	0.01447656	0.009593047	0.642326281	0.465814326	1.029330753	0.854103044
	WT	KO	dSARM rescue	dSARM E1170A	dSARM ARM-SAM	dSARM TIR

dSARM ARM-TIR	dSARM SAM
0.57362982	1.20786669
1.01929007	0.70870601
0.90203599	1.54349375
1.46759326	0.6737346

0.990637285	1.033450263
0.369708624	0.418497167
0.184854312	0.209248584
dSARM ARM-TIR	dSARM SAM

	02/22/21		2/25/21	
Filename	Uninjured	Injured	Uninjured	Injured
FRT2A82B_1dpe_2dpi_1.czi				
FRT2A82B_1dpe_2dpi_2				
FRT2A82B_1dpe_2dpi_3				
FRT2A82B_1dpe_2dpi_4				
FRT2A82B_1dpe_2dpi_5				
FRT2A82B_1dpe_2dpi_6				
FRT2A82B_1dpe_2dpi_7				
FRT2A82B_1dpe_2dpi_8				
FRT2A82B_1dpe_2dpi_9				
FRT2A82B_1dpe_2dpi_10				
FRT2A82B_1dpe_2dpi_11				
FRT2A82B_1dpe_2dpi_12				
FRT2A82B_1dpe_2dpi_13				
FRT2A82B_1dpe_2dpi_14				
FRT2A82B_1dpe_2dpi_15				
FRT2A82B_1dpe_2dpi_16				
FRT2A82B_1dpe_2dpi_17				
FRT2A82B_1dpe_2dpi_18				
FRT2A82B_1dpe_2dpi_19				
FRT2A82B_1dpe_2dpi_20				
AVG				
dSarm896_1dpe_2dpi_1.czi				
dSarm896_1dpe_2dpi_2.czi				
dSarm896_1dpe_2dpi_3.czi				
dSarm896_1dpe_2dpi_4.czi				
dSarm896_1dpe_2dpi_5.czi				
dSarm896_1dpe_2dpi_6.czi				
dSarm896_1dpe_2dpi_7				
dSarm896_1dpe_2dpi_8				
dSarm896_1dpe_2dpi_9				
dSarm896_1dpe_2dpi_10				
dSarm896_1dpe_2dpi_11				
dSarm896_1dpe_2dpi_12				
dSarm896_1dpe_2dpi_13				
dSarm896_1dpe_2dpi_14				
dSarm896_1dpe_2dpi_15				
dSarm896_1dpe_2dpi_16				
dSarm896_1dpe_2dpi_17				
dSarm896_1dpe_2dpi_18				
dSarm896_1dpe_2dpi_19				
dSarm896_1dpe_2dpi_20				
AVG				

dSarmRescue_1dpe_2dpi_1.czi	18.0	2.0	9.0	0.0
dSarmRescue_1dpe_2dpi_2.czi	18.0	2.0	13.0	0.0
dSarmRescue_1dpe_2dpi_3.czi	18.0	3.0	10.0	0.0
dSarmRescue_1dpe_2dpi_4.czi	20.0	2.0	15.0	0.0
dSarmRescue_1dpe_2dpi_5.czi	13.0	0.0	19.0	2.0
dSarmRescue_1dpe_2dpi_6.czi	20.0	1.0	24.0	4.0
dSarmRescue_1dpe_2dpi_7.czi	15.0	0.0	24.0	2.0
dSarmRescue_1dpe_2dpi_8.czi	17.0	0.0	7.0	0.0
dSarmRescue_1dpe_2dpi_9.czi	17.0	0.0	14.0	0.0
dSarmRescue_1dpe_2dpi_10.czi			11.0	2.0
dSarmRescue_1dpe_2dpi_11.czi			21.0	0.0
dSarmRescue_1dpe_2dpi_12.czi			17.0	1.0
dSarmRescue_1dpe_2dpi_13.czi			10.0	0.0
dSarmRescue_1dpe_2dpi_14.czi				
dSarmRescue_1dpe_2dpi_15.czi				
dSarmRescue_1dpe_2dpi_16.czi				
dSarmRescue_1dpe_2dpi_17				
dSarmRescue_1dpe_2dpi_18				
dSarmRescue_1dpe_2dpi_19				
dSarmRescue_1dpe_2dpi_20				
AVG	17.3	1.1	14.9	0.8
dSarmattPKO_1dpe_2dpi_1.czi	11.0	3.0	18.0	8.0
dSarmattPKO_1dpe_2dpi_2.czi	18.0	10.0	10.0	0.0
dSarmattPKO_1dpe_2dpi_3.czi	8.0	12.0	15.0	0.0
dSarmattPKO_1dpe_2dpi_4.czi	12.0	9.0	9.0	0.0
dSarmattPKO_1dpe_2dpi_5.czi	13.0	7.0	14.0	14.0
dSarmattPKO_1dpe_2dpi_6.czi	22.0	13.0	7.0	0.0
dSarmattPKO_1dpe_2dpi_7.czi			8.0	14.0
dSarmattPKO_1dpe_2dpi_8.czi			11.0	5.0
dSarmattPKO_1dpe_2dpi_9.czi			9.0	0.0
dSarmattPKO_1dpe_2dpi_10.czi			18.0	0.0
dSarmattPKO_1dpe_2dpi_11.czi				
dSarmattPKO_1dpe_2dpi_12.czi				
dSarmattPKO_1dpe_2dpi_13.czi				
dSarmattPKO_1dpe_2dpi_14.czi				
dSarmattPKO_1dpe_2dpi_15.czi				
dSarmattPKO_1dpe_2dpi_16.czi				
dSarmattPKO_1dpe_2dpi_17.czi				
dSarmattPKO_1dpe_2dpi_18				
dSarmattPKO_1dpe_2dpi_19				
dSarmattPKO_1dpe_2dpi_20				
AVG	14.0	9.0	11.9	4.1

dSarmE642A_1dpe_2dpi_1.czi	11.0	7.0	12	18
dSarmE642A_1dpe_2dpi_2.czi	12.0	11.0	16.0	10.0
dSarmE642A_1dpe_2dpi_3.czi	12.0	7.0	7	9
dSarmE642A_1dpe_2dpi_4.czi	11.0	8.0	10	5
dSarmE642A_1dpe_2dpi_5.czi	16.0	12.0	19.0	3.0
dSarmE642A_1dpe_2dpi_6.czi	12.0	10.0	12	10
dSarmE642A_1dpe_2dpi_7.czi	16.0	10.0	14	10
dSarmE642A_1dpe_2dpi_8.czi	14.0	11.0	9	13
dSarmE642A_1dpe_2dpi_9.czi	16.0	15.0	15	11
dSarmE642A_1dpe_2dpi_10.czi	18.0	14.0	20	7
dSarmE642A_1dpe_2dpi_11.czi	20.0	10.0	17	9
dSarmE642A_1dpe_2dpi_12.czi	15.0	13.0	12	13
dSarmE642A_1dpe_2dpi_13.czi	15.0	13.0	10	15
dSarmE642A_1dpe_2dpi_14.czi	20.0	7.0		
dSarmE642A_1dpe_2dpi_15.czi	12.0	6.0		
dSarmE642A_1dpe_2dpi_16.czi	16.0	7.0		
dSarmE642A_1dpe_2dpi_17.czi	18.0	0.0		
dSarmE642A_1dpe_2dpi_18	14.0	9.0		
dSarmE642A_1dpe_2dpi_19	19.0	15.0		
dSarmE642A_1dpe_2dpi_20				
AVG	15.1	9.7	13.3	10.2
dSarm^ARM^SAM_1dpe_2dpi_1.czi	12	0	10	0
dSarm^ARM^SAM_1dpe_2dpi_2-2.czi	13	0	10	0
dSarm^ARM^SAM_1dpe_2dpi_2.czi	16	0	10	0
dSarm^ARM^SAM_1dpe_2dpi_3.czi	16	0	6	3
dSarm^ARM^SAM_1dpe_2dpi_4.czi	18	0	9	0
dSarm^ARM^SAM_1dpe_2dpi_5.czi	17	0	13	0
dSarm^ARM^SAM_1dpe_2dpi_6.czi	18	1	8	0
dSarm^ARM^SAM_1dpe_2dpi_7.czi	17	0	9	2
dSarm^ARM^SAM_1dpe_2dpi_8.czi	19	0	7	0
dSarm^ARM^SAM_1dpe_2dpi_9.czi	14	3	7	0
dSarm^ARM^SAM_1dpe_2dpi_10.czi	13	0	14	0
dSarm^ARM^SAM_1dpe_2dpi_11.czi	11	2		
dSarm^ARM^SAM_1dpe_2dpi_12.czi	11	0		
dSarm^ARM^SAM_1dpe_2dpi_13.czi	11	4		
dSarm^ARM^SAM_1dpe_2dpi_14.czi	14	0		
dSarm^ARM^SAM_1dpe_2dpi_15.czi				
dSarm^ARM^SAM_1dpe_2dpi_16.czi				
dSarm^ARM^SAM_1dpe_2dpi_17				
dSarm^ARM^SAM_1dpe_2dpi_18				
dSarm^ARM^SAM_1dpe_2dpi_19				
dSarm^ARM^SAM_1dpe_2dpi_20				
AVG	14.66667	0.66666667	9.36363636	0.45454545

dSarm^SAM_1dpe_2dpi_1.czi			10	0
dSarm^SAM_1dpe_2dpi_2.czi	14.0	2.0	14	0
dSarm^SAM_1dpe_2dpi_3.czi	16.0	0	11	0
dSarm^SAM_1dpe_2dpi_4.czi	15.0	0.0	13	12
dSarm^SAM_1dpe_2dpi_5.czi	11.0	0.0	12	0
dSarm^SAM_1dpe_2dpi_6.czi	11.0	0.0	8	0
dSarm^SAM_1dpe_2dpi_7.czi	19.0	0.0	8	0
dSarm^SAM_1dpe_2dpi_8.czi	12.0	1.0	10	0
dSarm^SAM_1dpe_2dpi_9.czi	14.0	0.0	14	0
dSarm^SAM_1dpe_2dpi_10.czi			12	0
dSarm^SAM_1dpe_2dpi_11.czi			11	0
dSarm^SAM_1dpe_2dpi_12			11	0
dSarm^SAM_1dpe_2dpi_13				
dSarm^SAM_1dpe_2dpi_14				
dSarm^SAM_1dpe_2dpi_15				
dSarm^SAM_1dpe_2dpi_16				
dSarm^SAM_1dpe_2dpi_17				
dSarm^SAM_1dpe_2dpi_18				
dSarm^SAM_1dpe_2dpi_19				
dSarm^SAM_1dpe_2dpi_20				
AVG	14.0	0.4	11.2	1.0
dSarm^TIR_1dpe_2dpi_1.czi	4.0	12.0	10	15
dSarm^TIR_1dpe_2dpi_2.czi	18.0	10.0	20	13
dSarm^TIR_1dpe_2dpi_3.czi	15.0	7.0	14	10
dSarm^TIR_1dpe_2dpi_4.czi	12.0	15.0	10	15
dSarm^TIR_1dpe_2dpi_5	13.0	9.0	15	8
dSarm^TIR_1dpe_2dpi_6	15.0	1.0	22	15
dSarm^TIR_1dpe_2dpi_7	13.0	3.0	18	6
dSarm^TIR_1dpe_2dpi_8	16.0	8.0	8	9
dSarm^TIR_1dpe_2dpi_9	14.0	10.0	13	7
dSarm^TIR_1dpe_2dpi_10	14.0	8.0		
dSarm^TIR_1dpe_2dpi_11	11.0	4.0		
dSarm^TIR_1dpe_2dpi_12	15.0	11.0		
dSarm^TIR_1dpe_2dpi_13				
dSarm^TIR_1dpe_2dpi_14				
dSarm^TIR_1dpe_2dpi_15				
dSarm^TIR_1dpe_2dpi_16				
dSarm^TIR_1dpe_2dpi_17				
dSarm^TIR_1dpe_2dpi_18				
dSarm^TIR_1dpe_2dpi_19				
dSarm^TIR_1dpe_2dpi_20				
AVG	13.3	8.2	14.4	10.9

02/28/21		04/13/21		04/14/21	
Uninjured	Injured	Uninjured	Injured	Uninjured	Injured
		17	0	12	0
		15	0	15	0
		15	0	10	1
		19	0	14	0
		22	0	15	0
		15	1	17	0
		14	0	11	0
		17	0	13	0
		18	1	13	0
				16	0
				11	0
				13	0
		16.8888889	0.22222222	13.3333333	0.08333333
		18	9	12	16
		14	5	9	6
		15	0	12	3
		13	11	7	6
		17	6	14	4
		9	6	12	7
		13	11	15	1
		16	2	18	10
		11	9	21	10
		16	11	7	6
		23	10	13	8
		21	3	11	2
		23	2	12	2
				16	14
		16.0769231	6.53846154	12.7857143	6.78571429

19.0	0.0
11.0	0.0
12.0	0.0
11.0	0.0
18.0	1.0
15.0	4.0
13.0	0.0
17.0	0.0
12.0	0.0
14.2	0.6
14	6
12	6
17	3
24.0	9
13.0	5
15.0	0
23.0	0.0
16.9	4.1

13	13
11.0	11.0
19	12.0
14.0	18.0
15	1.0
14.0	10.0
16	6.0
23.0	18.0
15.6	11.1
11	0
6	9
20	0
13	0
7	0
14	4
9	0
12	0
10	7
11.3333333	2.22222222

16	0
17	0
11	7
11	11
14	0
26	6
15.8	4.0
9	8
13	9
18	5
15	11
10	9
16	13
9	7
15	11
13	1
9	8
12.7	8.2

17	18	0	9
15	14	0	5
15	15	0	0
19	13	0	11
22	17	0	6
15	9	1	6
14	13	0	11
17	16	0	2
18	11	1	9
12	16	0	11
15	23	0	10
10	21	1	3
14	23	0	2
15	12	0	16
17	9	0	6
11	12	0	3
13	7	0	6
13	14	0	4
16	12	0	7
11	15	0	1
13	18	0	10
	21		10
	7		6
	13		8
	11		2
	12		2
	16		14

	02/28/21		03/03/21		03/06
Filename	Uninjured	Injured	Uninjured	Injured	Uninjured
FRT2A82B_1dpe_7dpi_1.czi					
FRT2A82B_1dpe_7dpi_2					
FRT2A82B_1dpe_7dpi_3					
FRT2A82B_1dpe_7dpi_4					
FRT2A82B_1dpe_7dpi_5					
FRT2A82B_1dpe_7dpi_6					
FRT2A82B_1dpe_7dpi_7					
FRT2A82B_1dpe_7dpi_8					
FRT2A82B_1dpe_7dpi_9					
FRT2A82B_1dpe_7dpi_10					
FRT2A82B_1dpe_7dpi_11					
FRT2A82B_1dpe_7dpi_12					
FRT2A82B_1dpe_7dpi_13					
FRT2A82B_1dpe_7dpi_14					
FRT2A82B_1dpe_7dpi_15					
FRT2A82B_1dpe_7dpi_16					
FRT2A82B_1dpe_7dpi_17					
FRT2A82B_1dpe_7dpi_18					
FRT2A82B_1dpe_7dpi_19					
FRT2A82B_1dpe_7dpi_20					
AVG	#DIV/0!	#DIV/0!	#DIV/0!	#DIV/0!	#DIV/0!
dSarm896_1dpe_7dpi_1.czi					
dSarm896_1dpe_7dpi_2.czi					
dSarm896_1dpe_7dpi_3.czi					
dSarm896_1dpe_7dpi_4.czi					
dSarm896_1dpe_7dpi_5.czi					
dSarm896_1dpe_7dpi_6.czi					
dSarm896_1dpe_7dpi_7					
dSarm896_1dpe_7dpi_8					
dSarm896_1dpe_7dpi_9					
dSarm896_1dpe_7dpi_10					
dSarm896_1dpe_7dpi_11					
dSarm896_1dpe_7dpi_12					
dSarm896_1dpe_7dpi_13					
dSarm896_1dpe_7dpi_14					
dSarm896_1dpe_7dpi_15					
dSarm896_1dpe_7dpi_16					
dSarm896_1dpe_7dpi_17					
dSarm896_1dpe_7dpi_18					
dSarm896_1dpe_7dpi_19					
dSarm896_1dpe_7dpi_20					
AVG	#DIV/0!	#DIV/0!	#DIV/0!	#DIV/0!	#DIV/0!

dSarmRescue_1dpe_7dpi_1.czi	8	0	8	0	17.0
dSarmRescue_1dpe_7dpi_2.czi	6	0	6	0	13.0
dSarmRescue_1dpe_7dpi_3.czi	13	0	13	0	19.0
dSarmRescue_1dpe_7dpi_4.czi	12	2	12	2	11.0
dSarmRescue_1dpe_7dpi_5.czi			10.0	0.0	10.0
dSarmRescue_1dpe_7dpi_6.czi			12.0	0.0	12.0
dSarmRescue_1dpe_7dpi_7.czi			14.0	0.0	6.0
dSarmRescue_1dpe_7dpi_8.czi					6.0
dSarmRescue_1dpe_7dpi_9.czi			13.0	0.0	4.0
dSarmRescue_1dpe_7dpi_10.czi			9.0	0.0	
dSarmRescue_1dpe_7dpi_11.czi			12.0	0.0	
dSarmRescue_1dpe_7dpi_12.czi			12.0	0.0	
dSarmRescue_1dpe_7dpi_13.czi			13.0	0.0	
dSarmRescue_1dpe_7dpi_14.czi			9.0	1.0	
dSarmRescue_1dpe_7dpi_15.czi			11.0	0.0	
dSarmRescue_1dpe_7dpi_16.czi			8.0	0.0	
dSarmRescue_1dpe_7dpi_17			11.0	1.0	
dSarmRescue_1dpe_7dpi_18			4.0	0.0	
dSarmRescue_1dpe_7dpi_19			11.0	0.0	
dSarmRescue_1dpe_7dpi_20					
AVG	9.75	0.5	10.44444	0.22222	10.88889
dSarmattPKO_1dpe_7dpi_1.czi	11	0	11.0	0.0	9.0
dSarmattPKO_1dpe_7dpi_2.czi	14	6	14.0	7.0	11.0
dSarmattPKO_1dpe_7dpi_3.czi			17.0	16.0	5.0
dSarmattPKO_1dpe_7dpi_4.czi	5	11	11.0	0.0	9.0
dSarmattPKO_1dpe_7dpi_5.czi	11	9	10.0	0.0	12.0
dSarmattPKO_1dpe_7dpi_6.czi	9	13	16.0	21.0	13.0
dSarmattPKO_1dpe_7dpi_7.czi			9.0	0.0	4.0
dSarmattPKO_1dpe_7dpi_8.czi			11.0	0.0	7.0
dSarmattPKO_1dpe_7dpi_9.czi			17.0	13.0	4.0
dSarmattPKO_1dpe_7dpi_10.czi			12.0	0.0	
dSarmattPKO_1dpe_7dpi_11.czi			12.0	1.0	
dSarmattPKO_1dpe_7dpi_12.czi			17.0	7.0	
dSarmattPKO_1dpe_7dpi_13.czi			12.0	0.0	
dSarmattPKO_1dpe_7dpi_14.czi			11.0	6.0	
dSarmattPKO_1dpe_7dpi_15.czi			10.0	10.0	
dSarmattPKO_1dpe_7dpi_16.czi			15.0	11.0	
dSarmattPKO_1dpe_7dpi_17.czi			11.0	0.0	
dSarmattPKO_1dpe_7dpi_18			13.0	18.0	
dSarmattPKO_1dpe_7dpi_19			13.0	0.0	
dSarmattPKO_1dpe_7dpi_20					
AVG	10	7.8	12.73684	5.78947	8.222222

dSarmE642A_1dpe_7dpi_1.czi	14	9	12	1	
dSarmE642A_1dpe_7dpi_2.czi	12	13	13.0	12.0	
dSarmE642A_1dpe_7dpi_3.czi			9	12	
dSarmE642A_1dpe_7dpi_4.czi			18	11	
dSarmE642A_1dpe_7dpi_5.czi			12.0	16.0	
dSarmE642A_1dpe_7dpi_6.czi			22	11	
dSarmE642A_1dpe_7dpi_7.czi			17	18	
dSarmE642A_1dpe_7dpi_8.czi			10	10	
dSarmE642A_1dpe_7dpi_9.czi			12	11	
dSarmE642A_1dpe_7dpi_10.czi			23	9	
dSarmE642A_1dpe_7dpi_11.czi			12	4	
dSarmE642A_1dpe_7dpi_12.czi			12	6	
dSarmE642A_1dpe_7dpi_13.czi			10	7	
dSarmE642A_1dpe_7dpi_14.czi			17	8	
dSarmE642A_1dpe_7dpi_15.czi			12	10	
dSarmE642A_1dpe_7dpi_16.czi			17	7	
dSarmE642A_1dpe_7dpi_17.czi			19	6	
dSarmE642A_1dpe_7dpi_18			9	12	
dSarmE642A_1dpe_7dpi_19					
dSarmE642A_1dpe_7dpi_20					
AVG	13	11	14.22222	9.5	#DIV/0!
dSarm^ARM^SAM_1dpe_2dpi_1.czi			16	5	13
dSarm^ARM^SAM_1dpe_2dpi_2-2.czi			11	0	4
dSarm^ARM^SAM_1dpe_2dpi_2.czi			15	0	
dSarm^ARM^SAM_1dpe_2dpi_3.czi			17	0	
dSarm^ARM^SAM_1dpe_2dpi_4.czi			18	0	
dSarm^ARM^SAM_1dpe_2dpi_5.czi			8	0	
dSarm^ARM^SAM_1dpe_2dpi_6.czi			12	0	
dSarm^ARM^SAM_1dpe_2dpi_7.czi			23	8	
dSarm^ARM^SAM_1dpe_2dpi_8.czi			16	13	
dSarm^ARM^SAM_1dpe_2dpi_9.czi			12	0	
dSarm^ARM^SAM_1dpe_2dpi_10.czi			13	7	
dSarm^ARM^SAM_1dpe_2dpi_11.czi					
dSarm^ARM^SAM_1dpe_2dpi_12.czi					
dSarm^ARM^SAM_1dpe_2dpi_13.czi					
dSarm^ARM^SAM_1dpe_2dpi_14.czi					
dSarm^ARM^SAM_1dpe_2dpi_15.czi					
dSarm^ARM^SAM_1dpe_2dpi_16.czi					
dSarm^ARM^SAM_1dpe_2dpi_17					
dSarm^ARM^SAM_1dpe_2dpi_18					
dSarm^ARM^SAM_1dpe_2dpi_19					
dSarm^ARM^SAM_1dpe_2dpi_20					
AVG	#DIV/0!	#DIV/0!	14.63636	3	8.5

dSarm^SAM_1dpe_7dpi_1.czi	15	13	10	16	7
dSarm^SAM_1dpe_7dpi_2.czi	13	4	23.0	10.0	6.0
dSarm^SAM_1dpe_7dpi_3.czi	11	5	25	9	14
dSarm^SAM_1dpe_7dpi_4.czi	13	10	13	0	20
dSarm^SAM_1dpe_7dpi_5.czi			18	0	
dSarm^SAM_1dpe_7dpi_6.czi			21	10	
dSarm^SAM_1dpe_7dpi_7.czi			14	0	
dSarm^SAM_1dpe_7dpi_8.czi			16	8	
dSarm^SAM_1dpe_7dpi_9.czi			18	10	
dSarm^SAM_1dpe_7dpi_10.czi			11.0	0.0	
dSarm^SAM_1dpe_7dpi_11.czi			12.0	0.0	
dSarm^SAM_1dpe_7dpi_12			15	0	
dSarm^SAM_1dpe_7dpi_13					
dSarm^SAM_1dpe_7dpi_14					
dSarm^SAM_1dpe_7dpi_15					
dSarm^SAM_1dpe_7dpi_16					
dSarm^SAM_1dpe_7dpi_17					
dSarm^SAM_1dpe_7dpi_18					
dSarm^SAM_1dpe_7dpi_19					
dSarm^SAM_1dpe_7dpi_20					
AVG	13	8	16.33333	5.25	11.75
dSarm^TIR_1dpe_7dpi_1.czi	12	9	11	12	8
dSarm^TIR_1dpe_7dpi_2.czi	8	0	23	0	7
dSarm^TIR_1dpe_7dpi_3.czi	13	9	13	5	15
dSarm^TIR_1dpe_7dpi_4.czi	9	15	18	9	11
dSarm^TIR_1dpe_7dpi_5	7	6	13	8	9
dSarm^TIR_1dpe_7dpi_6			14	14	10
dSarm^TIR_1dpe_7dpi_7			11	11	10
dSarm^TIR_1dpe_7dpi_8			16	7	12
dSarm^TIR_1dpe_7dpi_9			18	9	15
dSarm^TIR_1dpe_7dpi_10			23	13	10
dSarm^TIR_1dpe_7dpi_11			9	5	12
dSarm^TIR_1dpe_7dpi_12			17	9	14
dSarm^TIR_1dpe_7dpi_13			16	8	11
dSarm^TIR_1dpe_7dpi_14			15	6	4
dSarm^TIR_1dpe_7dpi_15			13	6	
dSarm^TIR_1dpe_7dpi_16			7	13	
dSarm^TIR_1dpe_7dpi_17			8	0	
dSarm^TIR_1dpe_7dpi_18					
dSarm^TIR_1dpe_7dpi_19					
dSarm^TIR_1dpe_7dpi_20					
AVG	9.8	7.8	14.41176	7.94118	10.57143

[illegible]/21
Injured

#DIV/0!

#DIV/0!

0.0
0.0
3.0
0.0
0.0
0.0
0.0
0.0
0.0

0.33333	10.5	0.3

6.0
6.0
4.0
0.0
0.0
0.0
0.0
0.0
0.0

1.77778	11.1	5.0

#DIV/0!	14.1	9.7
0		
0		
0	13.5	2.5

1		
7.0		
6		
2		
4	14.8	5.6
12		
11		
10		
2		
6		
10		
0		
2		
9		
0		
8		
5		
14		
12		
7.21429	12.3	7.6

	03/26/21		03/27/21	
Filename	Uninjured	Injured	Uninjured	Injured
FRT2A82B_7dpe_7dpi_1.czi				
FRT2A82B_7dpe_7dpi_2				
FRT2A82B_7dpe_7dpi_3				
FRT2A82B_7dpe_7dpi_4				
FRT2A82B_7dpe_7dpi_5				
FRT2A82B_7dpe_7dpi_6				
FRT2A82B_7dpe_7dpi_7				
FRT2A82B_7dpe_7dpi_8				
FRT2A82B_7dpe_7dpi_9				
FRT2A82B_7dpe_7dpi_10				
FRT2A82B_7dpe_7dpi_11				
FRT2A82B_7dpe_7dpi_12				
FRT2A82B_7dpe_7dpi_13				
FRT2A82B_7dpe_7dpi_14				
FRT2A82B_7dpe_7dpi_15				
FRT2A82B_7dpe_7dpi_16				
FRT2A82B_7dpe_7dpi_17				
FRT2A82B_7dpe_7dpi_18				
FRT2A82B_7dpe_7dpi_19				
FRT2A82B_7dpe_7dpi_20				
AVG	#DIV/0!	#DIV/0!	#DIV/0!	#DIV/0!
dSarm896_7dpe_7dpi_1.czi				
dSarm896_7dpe_7dpi_2.czi				
dSarm896_7dpe_7dpi_3.czi				
dSarm896_7dpe_7dpi_4.czi				
dSarm896_7dpe_7dpi_5.czi				
dSarm896_7dpe_7dpi_6.czi				
dSarm896_7dpe_7dpi_7				
dSarm896_7dpe_7dpi_8				
dSarm896_7dpe_7dpi_9				
dSarm896_7dpe_7dpi_10				
dSarm896_7dpe_7dpi_11				
dSarm896_7dpe_7dpi_12				
dSarm896_7dpe_7dpi_13				
dSarm896_7dpe_7dpi_14				
dSarm896_7dpe_7dpi_15				
dSarm896_7dpe_7dpi_16				
dSarm896_7dpe_7dpi_17				
dSarm896_7dpe_7dpi_18				
dSarm896_7dpe_7dpi_19				
dSarm896_7dpe_7dpi_20				
AVG	#DIV/0!	#DIV/0!	#DIV/0!	#DIV/0!

dSarmRescue_7dpe_7dpi_1.czi	14	0	13.0	0.0
dSarmRescue_7dpe_7dpi_2.czi	10	0	7.0	0.0
dSarmRescue_7dpe_7dpi_3.czi	12	1	17.0	4.0
dSarmRescue_7dpe_7dpi_4.czi	7	0	10.0	0.0
dSarmRescue_7dpe_7dpi_5.czi			14.0	0.0
dSarmRescue_7dpe_7dpi_6.czi			7.0	0.0
dSarmRescue_7dpe_7dpi_7.czi			10.0	0.0
dSarmRescue_7dpe_7dpi_8.czi				
dSarmRescue_7dpe_7dpi_9.czi				
dSarmRescue_7dpe_7dpi_10.czi				
dSarmRescue_7dpe_7dpi_11.czi				
dSarmRescue_7dpe_7dpi_12.czi				
dSarmRescue_7dpe_7dpi_13.czi				
dSarmRescue_7dpe_7dpi_14.czi				
dSarmRescue_7dpe_7dpi_15.czi				
dSarmRescue_7dpe_7dpi_16.czi				
dSarmRescue_7dpe_7dpi_17				
dSarmRescue_7dpe_7dpi_18				
dSarmRescue_7dpe_7dpi_19				
dSarmRescue_7dpe_7dpi_20				
AVG	10.8	0.3	11.1	0.6
dSarmattPKO_7dpe_7dpi_1.czi			7.0	9.0
dSarmattPKO_7dpe_7dpi_2.czi			6.0	3.0
dSarmattPKO_7dpe_7dpi_3.czi			8.0	12.0
dSarmattPKO_7dpe_7dpi_4.czi				
dSarmattPKO_7dpe_7dpi_5.czi				
dSarmattPKO_7dpe_7dpi_6.czi				
dSarmattPKO_7dpe_7dpi_7.czi				
dSarmattPKO_7dpe_7dpi_8.czi				
dSarmattPKO_7dpe_7dpi_9.czi				
dSarmattPKO_7dpe_7dpi_10.czi				
dSarmattPKO_7dpe_7dpi_11.czi				
dSarmattPKO_7dpe_7dpi_12.czi				
dSarmattPKO_7dpe_7dpi_13.czi				
dSarmattPKO_7dpe_7dpi_14.czi				
dSarmattPKO_7dpe_7dpi_15.czi				
dSarmattPKO_7dpe_7dpi_16.czi				
dSarmattPKO_7dpe_7dpi_17.czi				
dSarmattPKO_7dpe_7dpi_18				
dSarmattPKO_7dpe_7dpi_19				
dSarmattPKO_7dpe_7dpi_20				
AVG	#DIV/0!	#DIV/0!	7.0	8.0

dSarmE642A_7dpe_7dpi_1.czi			12	9
dSarmE642A_7dpe_7dpi_2.czi			11.0	9.0
dSarmE642A_7dpe_7dpi_3.czi			9	6
dSarmE642A_7dpe_7dpi_4.czi			4	8
dSarmE642A_7dpe_7dpi_5.czi			20.0	4.0
dSarmE642A_7dpe_7dpi_6.czi			9	8
dSarmE642A_7dpe_7dpi_7.czi			14	7
dSarmE642A_7dpe_7dpi_8.czi			11	4
dSarmE642A_7dpe_7dpi_9.czi			22	7
dSarmE642A_7dpe_7dpi_10.czi			18	8
dSarmE642A_7dpe_7dpi_11.czi			9	10
dSarmE642A_7dpe_7dpi_12.czi			16	12
dSarmE642A_7dpe_7dpi_13.czi				
dSarmE642A_7dpe_7dpi_14.czi				
dSarmE642A_7dpe_7dpi_15.czi				
dSarmE642A_7dpe_7dpi_16.czi				
dSarmE642A_7dpe_7dpi_17.czi				
dSarmE642A_7dpe_7dpi_18				
dSarmE642A_7dpe_7dpi_19				
dSarmE642A_7dpe_7dpi_20				
AVG	#DIV/0!	#DIV/0!	12.9	7.7
dSarm^SAM_7dpe_7dpi_1.czi	11.0	6.0	8	8
dSarm^SAM_7dpe_7dpi_2.czi	15.0	13.0	16.0	1.0
dSarm^SAM_7dpe_7dpi_3.czi	10.0	3.0		
dSarm^SAM_7dpe_7dpi_4.czi	4.0	9.0		
dSarm^SAM_7dpe_7dpi_5.czi				
dSarm^SAM_7dpe_7dpi_6.czi				
dSarm^SAM_7dpe_7dpi_7.czi				
dSarm^SAM_7dpe_7dpi_8.czi				
dSarm^SAM_7dpe_7dpi_9.czi				
dSarm^SAM_7dpe_7dpi_10.czi				
dSarm^SAM_7dpe_7dpi_11.czi				
dSarm^SAM_7dpe_7dpi_12				
dSarm^SAM_7dpe_7dpi_13				
dSarm^SAM_7dpe_7dpi_14				
dSarm^SAM_7dpe_7dpi_15				
dSarm^SAM_7dpe_7dpi_16				
dSarm^SAM_7dpe_7dpi_17				
dSarm^SAM_7dpe_7dpi_18				
dSarm^SAM_7dpe_7dpi_19				
dSarm^SAM_7dpe_7dpi_20				
AVG	#DIV/0!	#DIV/0!	#DIV/0!	#DIV/0!
dSarm^TIR_7dpe_7dpi_1.czi	13.0	12.0	12	16

dSarm^TIR_7dpe_7dpi_2.czi	16.0	6.0	10	9
dSarm^TIR_7dpe_7dpi_3.czi	5.0	4.0	8	11
dSarm^TIR_7dpe_7dpi_4.czi			16	12
dSarm^TIR_7dpe_7dpi_5			6	12
dSarm^TIR_7dpe_7dpi_6			16	12
dSarm^TIR_7dpe_7dpi_7				
dSarm^TIR_7dpe_7dpi_8				
dSarm^TIR_7dpe_7dpi_9				
dSarm^TIR_7dpe_7dpi_10				
dSarm^TIR_7dpe_7dpi_11				
dSarm^TIR_7dpe_7dpi_12				
dSarm^TIR_7dpe_7dpi_13				
dSarm^TIR_7dpe_7dpi_14				
dSarm^TIR_7dpe_7dpi_15				
dSarm^TIR_7dpe_7dpi_16				
dSarm^TIR_7dpe_7dpi_17				
dSarm^TIR_7dpe_7dpi_18				
dSarm^TIR_7dpe_7dpi_19				
dSarm^TIR_7dpe_7dpi_20				
AVG	11.3	7.3	11.3	12.0

02/22/21	
Uninjured	Injured
#DIV/0!	#DIV/0!
#DIV/0!	#DIV/0!

#DIV/0!	#DIV/0!

#DIV/0!	#DIV/0!

#DIV/0!	#DIV/0!
#DIV/0!	c

#DIV/0! c

	02/13/21		02/18/21		05/0
Filename	Uninjured	Injured	Uninjured	Injured	Uninjured
FRT2A82B_7dpe_2dpi_1.czi	9.0	0.0	8	0	19
FRT2A82B_7dpe_2dpi_2	9.0	0.0	15	0	
FRT2A82B_7dpe_2dpi_3	21.0	0.0	7	0	
FRT2A82B_7dpe_2dpi_4	10.0	0.0	9	0	
FRT2A82B_7dpe_2dpi_5			14	0	
FRT2A82B_7dpe_2dpi_6			7	0	
FRT2A82B_7dpe_2dpi_7					
FRT2A82B_7dpe_2dpi_8					
FRT2A82B_7dpe_2dpi_9					
FRT2A82B_7dpe_2dpi_10					
FRT2A82B_7dpe_2dpi_11					
FRT2A82B_7dpe_2dpi_12					
FRT2A82B_7dpe_2dpi_13					
FRT2A82B_7dpe_2dpi_14					
FRT2A82B_7dpe_2dpi_15					
FRT2A82B_7dpe_2dpi_16					
FRT2A82B_7dpe_2dpi_17					
FRT2A82B_7dpe_2dpi_18					
FRT2A82B_7dpe_2dpi_19					
FRT2A82B_7dpe_2dpi_20					
AVG	12.3	0.0	10.0	0.0	
dSarm896_7dpe_2dpi_1.czi	18.0	17.0	19	0	
dSarm896_7dpe_2dpi_2.czi	8.0	18.0	10	1	
dSarm896_7dpe_2dpi_3.czi	12.0	12.0	26	1	
dSarm896_7dpe_2dpi_4.czi	20.0	9.0	0	7	
dSarm896_7dpe_2dpi_5.czi	9.0	4.0	18	0	
dSarm896_7dpe_2dpi_6.czi	6.0	20.0	8	6	
dSarm896_7dpe_2dpi_7	11.0	21.0	19	21	
dSarm896_7dpe_2dpi_8	11.0	11.0	11	15	
dSarm896_7dpe_2dpi_9	9.0	11.0			
dSarm896_7dpe_2dpi_10	14.0	12.0			
dSarm896_7dpe_2dpi_11	8.0	14.0			
dSarm896_7dpe_2dpi_12	15.0	15.0			
dSarm896_7dpe_2dpi_13					
dSarm896_7dpe_2dpi_14					
dSarm896_7dpe_2dpi_15					
dSarm896_7dpe_2dpi_16					
dSarm896_7dpe_2dpi_17					
dSarm896_7dpe_2dpi_18					
dSarm896_7dpe_2dpi_19					
dSarm896_7dpe_2dpi_20					
AVG	11.8	13.7	13.9	6.4	

dSarmRescue_7dpe_2dpi_1.czi	17.0	0.0	13.0	0.0	6.0
dSarmRescue_7dpe_2dpi_2.czi	11.0	0.0	18.0	1.0	9.0
dSarmRescue_7dpe_2dpi_3.czi	12.0	0.0	8.0	4.0	12.0
dSarmRescue_7dpe_2dpi_4.czi	17.0	2.0	15.0	0	9.0
dSarmRescue_7dpe_2dpi_5.czi			16.0	2.0	13.0
dSarmRescue_7dpe_2dpi_6.czi	6.0	0.0	17.0	0.0	16.0
dSarmRescue_7dpe_2dpi_7.czi	9.0	0.0	13.0	1	14.0
dSarmRescue_7dpe_2dpi_8.czi			9.0	0	16.0
dSarmRescue_7dpe_2dpi_9.czi			8.0	0	14.0
dSarmRescue_7dpe_2dpi_10.czi					14.0
dSarmRescue_7dpe_2dpi_11.czi					13.0
dSarmRescue_7dpe_2dpi_12.czi					7.0
dSarmRescue_7dpe_2dpi_13.czi					
dSarmRescue_7dpe_2dpi_14.czi					
dSarmRescue_7dpe_2dpi_15.czi					
dSarmRescue_7dpe_2dpi_16.czi					
dSarmRescue_7dpe_2dpi_17					
dSarmRescue_7dpe_2dpi_18					
dSarmRescue_7dpe_2dpi_19					
dSarmRescue_7dpe_2dpi_20					
AVG	12.0	0.3	13.0	0.9	11.9
dSarmattPKO_7dpe_2dpi_1.czi	9.0	12.0	12.0	20.0	
dSarmattPKO_7dpe_2dpi_2.czi	10.0	9	23.0	6.0	
dSarmattPKO_7dpe_2dpi_3.czi	10.0	10.0	17.0	4.0	
dSarmattPKO_7dpe_2dpi_4.czi	2.0	19.0	13.0	10	
dSarmattPKO_7dpe_2dpi_5.czi	6.0	16.0	6.0	17	
dSarmattPKO_7dpe_2dpi_6.czi	11.0	15.0	12.0	16	
dSarmattPKO_7dpe_2dpi_7.czi	14.0	6.0	10.0	13	
dSarmattPKO_7dpe_2dpi_8.czi	1.0	9.0	18.0	12	
dSarmattPKO_7dpe_2dpi_9.czi	11.0	19.0			
dSarmattPKO_7dpe_2dpi_10.czi	10.0	7.0			
dSarmattPKO_7dpe_2dpi_11.czi	9.0	10.0			
dSarmattPKO_7dpe_2dpi_12.czi	12.0	2.0			
dSarmattPKO_7dpe_2dpi_13.czi	16.0	7.0			
dSarmattPKO_7dpe_2dpi_14.czi	16.0	12.0			
dSarmattPKO_7dpe_2dpi_15.czi	7.0	11.0			
dSarmattPKO_7dpe_2dpi_16.czi					
dSarmattPKO_7dpe_2dpi_17.czi					
dSarmattPKO_7dpe_2dpi_18					
dSarmattPKO_7dpe_2dpi_19					
dSarmattPKO_7dpe_2dpi_20					
AVG	9.6	10.9	13.9	12.3	

dSarmE642A_7dpe_2dpi_1.czi	10.0	5.0	8.0	14	
dSarmE642A_7dpe_2dpi_2.czi	18.0	20.0	13.0	16	
dSarmE642A_7dpe_2dpi_3.czi	15.0	13.0	8	0	
dSarmE642A_7dpe_2dpi_4.czi	19.0	7.0	14.0	12	
dSarmE642A_7dpe_2dpi_5.czi	6.0	17.0	12.0	13.0	
dSarmE642A_7dpe_2dpi_6.czi	2.0	16.0	14.0	11	
dSarmE642A_7dpe_2dpi_7.czi	10.0	12.0	15.0	13	
dSarmE642A_7dpe_2dpi_8.czi	12.0	0.0	15.0	16	
dSarmE642A_7dpe_2dpi_9.czi	14.0	0.0	14.0	7	
dSarmE642A_7dpe_2dpi_10.czi					
dSarmE642A_7dpe_2dpi_11.czi	18.0	12.0			
dSarmE642A_7dpe_2dpi_12.czi	12.0	9.0			
dSarmE642A_7dpe_2dpi_13.czi	12.0	7.0			
dSarmE642A_7dpe_2dpi_14.czi	17.0	3.0			
dSarmE642A_7dpe_2dpi_15.czi	15.0	13.0			
dSarmE642A_7dpe_2dpi_16.czi	6.0	10.0			
dSarmE642A_7dpe_2dpi_17.czi	9.0	21.0			
dSarmE642A_7dpe_2dpi_18	16.0	15.0			
dSarmE642A_7dpe_2dpi_19					
dSarmE642A_7dpe_2dpi_20					
AVG	12.4	10.6	12.6	11.3	#DIV/0!
dSarm^SAM_7dpe_2dpi_1.czi					12
dSarm^SAM_7dpe_2dpi_2.czi					8.0
dSarm^SAM_7dpe_2dpi_3.czi					11
dSarm^SAM_7dpe_2dpi_4.czi					24
dSarm^SAM_7dpe_2dpi_5.czi					12
dSarm^SAM_7dpe_2dpi_6.czi					12
dSarm^SAM_7dpe_2dpi_7.czi					5
dSarm^SAM_7dpe_2dpi_8.czi					14
dSarm^SAM_7dpe_2dpi_9.czi					16
dSarm^SAM_7dpe_2dpi_10.czi					9.0
dSarm^SAM_7dpe_2dpi_11.czi					13.0
dSarm^SAM_7dpe_2dpi_12					15
dSarm^SAM_7dpe_2dpi_13					8
dSarm^SAM_7dpe_2dpi_14					10
dSarm^SAM_7dpe_2dpi_15					17
dSarm^SAM_7dpe_2dpi_16					
dSarm^SAM_7dpe_2dpi_17					
dSarm^SAM_7dpe_2dpi_18					
dSarm^SAM_7dpe_2dpi_19					
dSarm^SAM_7dpe_2dpi_20					
AVG	#DIV/0!	######			12.4
dSarm^TIR_7dpe_2dpi_1.czi	6.0	4.0	21	20	10

dSarm^TIR_7dpe_2dpi_2.czi	8.0	17.0	12	15	15
dSarm^TIR_7dpe_2dpi_3.czi	8.0	8.0	16	12	13
dSarm^TIR_7dpe_2dpi_4.czi	11.0	6.0	12	20	
dSarm^TIR_7dpe_2dpi_5	9.0	8.0	7	15	
dSarm^TIR_7dpe_2dpi_6	9.0	6.0	19	2	
dSarm^TIR_7dpe_2dpi_7	15.0	14.0	6	25	
dSarm^TIR_7dpe_2dpi_8	13.0	13.0	6	7	
dSarm^TIR_7dpe_2dpi_9	13.0	15.0	15	13	
dSarm^TIR_7dpe_2dpi_10					
dSarm^TIR_7dpe_2dpi_11					
dSarm^TIR_7dpe_2dpi_12					
dSarm^TIR_7dpe_2dpi_13					
dSarm^TIR_7dpe_2dpi_14					
dSarm^TIR_7dpe_2dpi_15					
dSarm^TIR_7dpe_2dpi_16					
dSarm^TIR_7dpe_2dpi_17					
dSarm^TIR_7dpe_2dpi_18					
dSarm^TIR_7dpe_2dpi_19					
dSarm^TIR_7dpe_2dpi_20					
AVG	10.2	10.1	12.7	14.3	12.7
dSarm^ARM^SAM_1dpe_2dpi_1.czi					13
dSarm^ARM^SAM_1dpe_2dpi_2.czi					10
dSarm^ARM^SAM_1dpe_2dpi_3.czi					16
dSarm^ARM^SAM_1dpe_2dpi_4.czi					11
dSarm^ARM^SAM_1dpe_2dpi_5.czi					
dSarm^ARM^SAM_1dpe_2dpi_6.czi					
dSarm^ARM^SAM_1dpe_2dpi_7.czi					
dSarm^ARM^SAM_1dpe_2dpi_8.czi					
dSarm^ARM^SAM_1dpe_2dpi_9.czi					
dSarm^ARM^SAM_1dpe_2dpi_10.czi					
dSarm^ARM^SAM_1dpe_2dpi_11.czi					
dSarm^ARM^SAM_1dpe_2dpi_12.czi					
dSarm^ARM^SAM_1dpe_2dpi_13.czi					
dSarm^ARM^SAM_1dpe_2dpi_14.czi					
dSarm^ARM^SAM_1dpe_2dpi_15.czi					
dSarm^ARM^SAM_1dpe_2dpi_16.czi					
dSarm^ARM^SAM_1dpe_2dpi_17					
dSarm^ARM^SAM_1dpe_2dpi_18					
dSarm^ARM^SAM_1dpe_2dpi_19					
dSarm^ARM^SAM_1dpe_2dpi_20					
AVG					

[illegible]/21	05/12/21		05/23/21		05/18/21	
Injured	Uninjured	Injured	Uninjured	Injured	Uninjured	Injured
0	12	0				
	8	0				
	4	0				
	15	0				
	11	0				
	11	0				
	17	0				
	11	0				
	18	0				
	12	0				
	13	0				
	17	0				
	12.4	0.0				
	21	17				
	18	13				
	4	18				
	13	11				
	9	12				
	10	12				
	6	11				
	16	6				
	16	17				
	12.6	13.0				

0.0	7	0
0.0	13	0
0.0	17	0
0.0	6	0
0.0	9	0
0.0		
0.0		
5.0		
0.0		
0.0		
0.0		
0.0		

0.4

#DIV/0!

9	11	17
15.0	11	10.0
9	12	18
6	16	8
7	9	9
9	8	13
8	4	10
14	9	9
13		
4.0		
0.0		
9		
16		
21		
23		
10.9	10.0	11.8
10		

12
7

9.7

0	5	0	13	0	7	0
0	15	0	7	0	8	0
0	9	0	10	0	7	0
0	11	0	8	0	9	0
	12	0	9	0	10	0
	14	0	14	0	8	0
					10	0
	7	0			8	0

	02/13/21		02/1
Filename	Uninjured	Injured	Uninjured
FRT2A82B_7dpe_7dpi_1.czi	9.0	0.0	10
FRT2A82B_7dpe_7dpi_2	7.0	0.0	9
FRT2A82B_7dpe_7dpi_3			
FRT2A82B_7dpe_7dpi_4			
FRT2A82B_7dpe_7dpi_5			
FRT2A82B_7dpe_7dpi_6			
FRT2A82B_7dpe_7dpi_7			
FRT2A82B_7dpe_7dpi_8			
FRT2A82B_7dpe_7dpi_9			
FRT2A82B_7dpe_7dpi_10			
FRT2A82B_7dpe_7dpi_11			
FRT2A82B_7dpe_7dpi_12			
FRT2A82B_7dpe_7dpi_13			
FRT2A82B_7dpe_7dpi_14			
FRT2A82B_7dpe_7dpi_15			
FRT2A82B_7dpe_7dpi_16			
FRT2A82B_7dpe_7dpi_17			
FRT2A82B_7dpe_7dpi_18			
FRT2A82B_7dpe_7dpi_19			
FRT2A82B_7dpe_7dpi_20			
AVG	8.0	0.0	9.5
dSarm896_7dpe_7dpi_1.czi			8
dSarm896_7dpe_7dpi_2.czi	21.0	0.0	16
dSarm896_7dpe_7dpi_3.czi	13.0	9.0	13
dSarm896_7dpe_7dpi_4.czi	10.0	6.0	
dSarm896_7dpe_7dpi_5.czi	15.0	0.0	
dSarm896_7dpe_7dpi_6.czi	6.0	16.0	
dSarm896_7dpe_7dpi_7	16.0	18.0	
dSarm896_7dpe_7dpi_8			
dSarm896_7dpe_7dpi_9			
dSarm896_7dpe_7dpi_10			
dSarm896_7dpe_7dpi_11			
dSarm896_7dpe_7dpi_12			
dSarm896_7dpe_7dpi_13			
dSarm896_7dpe_7dpi_14			
dSarm896_7dpe_7dpi_15			
dSarm896_7dpe_7dpi_16			
dSarm896_7dpe_7dpi_17			
dSarm896_7dpe_7dpi_18			
dSarm896_7dpe_7dpi_19			
dSarm896_7dpe_7dpi_20			
AVG	13.5	8.2	12.3

dSarmRescue_7dpe_7dpi_1.czi	10.0	0.0	12.0
dSarmRescue_7dpe_7dpi_2.czi	5.0	0.0	23.0
dSarmRescue_7dpe_7dpi_3.czi	11.0	0.0	11.0
dSarmRescue_7dpe_7dpi_4.czi	11.0	0.0	5.0
dSarmRescue_7dpe_7dpi_5.czi	11.0	0.0	12.0
dSarmRescue_7dpe_7dpi_6.czi	14.0	0.0	14.0
dSarmRescue_7dpe_7dpi_7.czi	17.0	0.0	12.0
dSarmRescue_7dpe_7dpi_8.czi	9.0	0.0	23.0
dSarmRescue_7dpe_7dpi_9.czi	14.0	0.0	13.0
dSarmRescue_7dpe_7dpi_10.czi	13.0	0.0	12.0
dSarmRescue_7dpe_7dpi_11.czi	12.0	0.0	8.0
dSarmRescue_7dpe_7dpi_12.czi	3.0	0.0	12.0
dSarmRescue_7dpe_7dpi_13.czi	8.0	0.0	24.0
dSarmRescue_7dpe_7dpi_14.czi	7.0	0.0	
dSarmRescue_7dpe_7dpi_15.czi	13.0	0.0	
dSarmRescue_7dpe_7dpi_16.czi	15.0	5.0	
dSarmRescue_7dpe_7dpi_17	16.0	0.0	
dSarmRescue_7dpe_7dpi_18			
dSarmRescue_7dpe_7dpi_19			
dSarmRescue_7dpe_7dpi_20			
AVG	11.1	0.3	13.9
dSarmattPKO_7dpe_7dpi_1.czi	19.0	16.0	12.0
dSarmattPKO_7dpe_7dpi_2.czi	15.0	2.0	22.0
dSarmattPKO_7dpe_7dpi_3.czi	16.0	10.0	12.0
dSarmattPKO_7dpe_7dpi_4.czi	6.0	9.0	9.0
dSarmattPKO_7dpe_7dpi_5.czi	13.0	8.0	13.0
dSarmattPKO_7dpe_7dpi_6.czi	13.0	7.0	9.0
dSarmattPKO_7dpe_7dpi_7.czi	12.0	6.0	16.0
dSarmattPKO_7dpe_7dpi_8.czi	13.0	0.0	10.0
dSarmattPKO_7dpe_7dpi_9.czi	8.0	9.0	14.0
dSarmattPKO_7dpe_7dpi_10.czi	17.0	11.0	6.0
dSarmattPKO_7dpe_7dpi_11.czi	12.0	1.0	12.0
dSarmattPKO_7dpe_7dpi_12.czi	18.0	1.0	17.0
dSarmattPKO_7dpe_7dpi_13.czi	9.0	7.0	10.0
dSarmattPKO_7dpe_7dpi_14.czi	11.0	15.0	10.0
dSarmattPKO_7dpe_7dpi_15.czi	12.0	1.0	
dSarmattPKO_7dpe_7dpi_16.czi	16.0	13.0	
dSarmattPKO_7dpe_7dpi_17.czi	3.0	12.0	
dSarmattPKO_7dpe_7dpi_18	17.0	4.0	
dSarmattPKO_7dpe_7dpi_19			
dSarmattPKO_7dpe_7dpi_20			
AVG	12.8	7.3	12.3

dSarmE642A_7dpe_7dpi_1.czi	12.0	7.0	11
dSarmE642A_7dpe_7dpi_2.czi	13.0	11.0	14.0
dSarmE642A_7dpe_7dpi_3.czi	13.0	12.0	14
dSarmE642A_7dpe_7dpi_4.czi	16.0	6.0	4
dSarmE642A_7dpe_7dpi_5.czi	11.0	5.0	16.0
dSarmE642A_7dpe_7dpi_6.czi	12.0	13.0	10
dSarmE642A_7dpe_7dpi_7.czi	3.0	13.0	19
dSarmE642A_7dpe_7dpi_8.czi	5.0	4.0	9
dSarmE642A_7dpe_7dpi_9.czi	11.0	6.0	21
dSarmE642A_7dpe_7dpi_10.czi	8.0	9.0	11
dSarmE642A_7dpe_7dpi_11.czi	8.0	22.0	16
dSarmE642A_7dpe_7dpi_12.czi	12.0	18.0	14
dSarmE642A_7dpe_7dpi_13.czi	10.0	14.0	16
dSarmE642A_7dpe_7dpi_14.czi	8.0	15.0	22
dSarmE642A_7dpe_7dpi_15.czi	8.0	12.0	9
dSarmE642A_7dpe_7dpi_16.czi	9.0	12.0	8
dSarmE642A_7dpe_7dpi_17.czi	12.0	11.0	
dSarmE642A_7dpe_7dpi_18	13.0	22.0	
dSarmE642A_7dpe_7dpi_19			
dSarmE642A_7dpe_7dpi_20			
AVG	10.2	11.8	13.4
dSarm^SAM_7dpe_7dpi_1.czi	7.0	6.0	
dSarm^SAM_7dpe_7dpi_2.czi	13.0	16.0	
dSarm^SAM_7dpe_7dpi_3.czi	8.0	10.0	
dSarm^SAM_7dpe_7dpi_4.czi	9.0	11.0	
dSarm^SAM_7dpe_7dpi_5.czi	13.0	13.0	
dSarm^SAM_7dpe_7dpi_6.czi	13.0	14.0	
dSarm^SAM_7dpe_7dpi_7.czi	11.0	12.0	
dSarm^SAM_7dpe_7dpi_8.czi	10.0	10.0	
dSarm^SAM_7dpe_7dpi_9.czi	11.0	9.0	
dSarm^SAM_7dpe_7dpi_10.czi	12.0	18.0	
dSarm^SAM_7dpe_7dpi_11.czi	16.0	16.0	
dSarm^SAM_7dpe_7dpi_12	17.0	7.0	
dSarm^SAM_7dpe_7dpi_13			
dSarm^SAM_7dpe_7dpi_14			
dSarm^SAM_7dpe_7dpi_15			
dSarm^SAM_7dpe_7dpi_16			
dSarm^SAM_7dpe_7dpi_17			
dSarm^SAM_7dpe_7dpi_18			
dSarm^SAM_7dpe_7dpi_19			
dSarm^SAM_7dpe_7dpi_20			
AVG	11.7	11.8	#DIV/0!
dSarm^TIR_7dpe_7dpi_1.czi	12.0	7.0	11

dSarm^TIR_7dpe_7dpi_2.czi			7
dSarm^TIR_7dpe_7dpi_3.czi	10.0	9.0	
dSarm^TIR_7dpe_7dpi_4.czi	15.0	10.0	
dSarm^TIR_7dpe_7dpi_5	13.0	0.0	
dSarm^TIR_7dpe_7dpi_6			
dSarm^TIR_7dpe_7dpi_7			
dSarm^TIR_7dpe_7dpi_8			
dSarm^TIR_7dpe_7dpi_9			
dSarm^TIR_7dpe_7dpi_10			
dSarm^TIR_7dpe_7dpi_11			
dSarm^TIR_7dpe_7dpi_12			
dSarm^TIR_7dpe_7dpi_13			
dSarm^TIR_7dpe_7dpi_14			
dSarm^TIR_7dpe_7dpi_15			
dSarm^TIR_7dpe_7dpi_16			
dSarm^TIR_7dpe_7dpi_17			
dSarm^TIR_7dpe_7dpi_18			
dSarm^TIR_7dpe_7dpi_19			
dSarm^TIR_7dpe_7dpi_20			
AVG	12.5	6.5	9.0
dSarm^ARM^SAM_1dpe_2dpi_1.czi			
dSarm^ARM^SAM_1dpe_2dpi_2.czi			
dSarm^ARM^SAM_1dpe_2dpi_3.czi			
dSarm^ARM^SAM_1dpe_2dpi_4.czi			
dSarm^ARM^SAM_1dpe_2dpi_5.czi			
dSarm^ARM^SAM_1dpe_2dpi_6.czi			
dSarm^ARM^SAM_1dpe_2dpi_7.czi			
dSarm^ARM^SAM_1dpe_2dpi_8.czi			
dSarm^ARM^SAM_1dpe_2dpi_9.czi			
dSarm^ARM^SAM_1dpe_2dpi_10.czi			
dSarm^ARM^SAM_1dpe_2dpi_11.czi			
dSarm^ARM^SAM_1dpe_2dpi_12.czi			
dSarm^ARM^SAM_1dpe_2dpi_13.czi			
dSarm^ARM^SAM_1dpe_2dpi_14.czi			
dSarm^ARM^SAM_1dpe_2dpi_15.czi			
dSarm^ARM^SAM_1dpe_2dpi_16.czi			
dSarm^ARM^SAM_1dpe_2dpi_17			
dSarm^ARM^SAM_1dpe_2dpi_18			
dSarm^ARM^SAM_1dpe_2dpi_19			
dSarm^ARM^SAM_1dpe_2dpi_20			
AVG			

.8/21	02/22/21		3/25/21		4/16/21	
Injured	Uninjured	Injured	Uninjured	Injured	Uninjured	Injured
0					16	0
0					13	0
					22	0
					8	0
					15	0
					8	0
					15	0
					13	0
					11	0
					14	0
					8	0
0.0	#DIV/0!	#DIV/0!			13.9	0.0
14	14	16			20	6
13	5	7			13	16
10	18	3			16	5
	19	0			9	0
	22	0			12	20
					23	17
					3	17
12.3	15.6	5.2			13.7	11.6

2.0		
2.0		
0.0		
0.0		
0.0		
0.0		
1.0		
0.0		
0.0		
0.0		
0.0		
0.0		
2.0		
0.5	12.3	0.4
8.0		
15.0		
0.0		
12.0		
10.0		
15.0		
2.0		
9.0		
4.0		
14.0		
7.0		
15.0		
20.0		
4.0		
9.6	12.6	8.3

12					
11.0					
17					
12					
2.0					
7					
13					
12					
11					
5					
14					
3					
11					
14					
11					
16					
10.7	11.7	11.3			
				7	11.0
				6	9
				17	16
			X		17
				19	13
				9	10
				9	9
				12	7
				8	8
#DIV/0!	#DIV/0!	#DIV/0!		10.9	11.1
11	19	15		10.0	5.0

0	12	17	19.0	9.0
	23	7	9.0	14.0
	7	8	11.0	14.0
	19	0	8.0	16.0
	17	8	3.0	8.0
	24	8	15.0	10.0
			5.0	12.0
			8.0	14.0
			13.0	4.0
			8.0	10.0
5.5	17.3	9.0	9.9	10.5
			12	0
			15	0
			13	1
			8	0
			10	0
			10	0
			9	0
			10	0
			9	0
			10.6666667	0.1111111

4/18/21		5/25/21	
Uninjured	Injured	Uninjured	Injured
16	0		
16	0		
12	0		
13	0		
11	0		
8	0		
13	0		
6	0		
22	0		
13	0		
13.0	0.0		
7	14		
11	22		
24	5		
14.0	13.7		

14	4
9	2
12	5
9	13
19	9
6	18
14	7
17	18
21	7
14	23
13	7
6	5
10	5
9	16
12.4	9.9

5	0
11	0
13	0
9	0
12	0
8	0
8	0
8	0
7	0
7	0
7	0
10	0
9	0
6	0
11	0

9.0	12.0	13.0	11
12.0	11.0	22.0	12

14.0
11.0

	04/15/21		04/16/21	
Filename	Uninjured	Injured	Uninjured	Injured
FRT2A82B_7dpe_7dpi_1.czi				
FRT2A82B_7dpe_7dpi_2				
FRT2A82B_7dpe_7dpi_3				
FRT2A82B_7dpe_7dpi_4				
FRT2A82B_7dpe_7dpi_5				
FRT2A82B_7dpe_7dpi_6				
FRT2A82B_7dpe_7dpi_7				
FRT2A82B_7dpe_7dpi_8				
FRT2A82B_7dpe_7dpi_9				
FRT2A82B_7dpe_7dpi_10				
FRT2A82B_7dpe_7dpi_11				
FRT2A82B_7dpe_7dpi_12				
FRT2A82B_7dpe_7dpi_13				
FRT2A82B_7dpe_7dpi_14				
FRT2A82B_7dpe_7dpi_15				
FRT2A82B_7dpe_7dpi_16				
FRT2A82B_7dpe_7dpi_17				
FRT2A82B_7dpe_7dpi_18				
FRT2A82B_7dpe_7dpi_19				
FRT2A82B_7dpe_7dpi_20				
AVG	#DIV/0!	#DIV/0!	#DIV/0!	#DIV/0!
dSarm896_7dpe_7dpi_1.czi				
dSarm896_7dpe_7dpi_2.czi				
dSarm896_7dpe_7dpi_3.czi				
dSarm896_7dpe_7dpi_4.czi				
dSarm896_7dpe_7dpi_5.czi				
dSarm896_7dpe_7dpi_6.czi				
dSarm896_7dpe_7dpi_7				
dSarm896_7dpe_7dpi_8				
dSarm896_7dpe_7dpi_9				
dSarm896_7dpe_7dpi_10				
dSarm896_7dpe_7dpi_11				
dSarm896_7dpe_7dpi_12				
dSarm896_7dpe_7dpi_13				
dSarm896_7dpe_7dpi_14				
dSarm896_7dpe_7dpi_15				
dSarm896_7dpe_7dpi_16				
dSarm896_7dpe_7dpi_17				
dSarm896_7dpe_7dpi_18				
dSarm896_7dpe_7dpi_19				
dSarm896_7dpe_7dpi_20				
AVG	#DIV/0!	#DIV/0!	#DIV/0!	#DIV/0!

dSarmRescue_7dpe_7dpi_1.czi	6.0	0.0	12.0	1.0
dSarmRescue_7dpe_7dpi_2.czi	8.0	2.0	7.0	0.0
dSarmRescue_7dpe_7dpi_3.czi	12.0	0.0	8.0	0.0
dSarmRescue_7dpe_7dpi_4.czi	12.0	0.0	16.0	1.0
dSarmRescue_7dpe_7dpi_5.czi	10.0	0.0	12.0	1.0
dSarmRescue_7dpe_7dpi_6.czi	10.0	3.0	9.0	2.0
dSarmRescue_7dpe_7dpi_7.czi	8.0	0.0	8.0	1.0
dSarmRescue_7dpe_7dpi_8.czi	13.0	4.0	4.0	0.0
dSarmRescue_7dpe_7dpi_9.czi	8.0	1.0	4.0	0.0
dSarmRescue_7dpe_7dpi_10.czi	9.0	0.0	9.0	3.0
dSarmRescue_7dpe_7dpi_11.czi	5.0	0.0	11.0	0.0
dSarmRescue_7dpe_7dpi_12.czi	9.0	0.0	10.0	1.0
dSarmRescue_7dpe_7dpi_13.czi	12.0	0.0	9.0	0.0
dSarmRescue_7dpe_7dpi_14.czi				
dSarmRescue_7dpe_7dpi_15.czi				
dSarmRescue_7dpe_7dpi_16.czi				
dSarmRescue_7dpe_7dpi_17				
dSarmRescue_7dpe_7dpi_18				
dSarmRescue_7dpe_7dpi_19				
dSarmRescue_7dpe_7dpi_20				
AVG	9.4	0.8	9.2	0.8
dSarmattPKO_7dpe_7dpi_1.czi	17.0	13.0	13	14
dSarmattPKO_7dpe_7dpi_2.czi	14.0	12.0	13.0	10.0
dSarmattPKO_7dpe_7dpi_3.czi	8.0	14.0	14	8
dSarmattPKO_7dpe_7dpi_4.czi				
dSarmattPKO_7dpe_7dpi_5.czi				
dSarmattPKO_7dpe_7dpi_6.czi				
dSarmattPKO_7dpe_7dpi_7.czi				
dSarmattPKO_7dpe_7dpi_8.czi				
dSarmattPKO_7dpe_7dpi_9.czi				
dSarmattPKO_7dpe_7dpi_10.czi				
dSarmattPKO_7dpe_7dpi_11.czi				
dSarmattPKO_7dpe_7dpi_12.czi				
dSarmattPKO_7dpe_7dpi_13.czi				
dSarmattPKO_7dpe_7dpi_14.czi				
dSarmattPKO_7dpe_7dpi_15.czi				
dSarmattPKO_7dpe_7dpi_16.czi				
dSarmattPKO_7dpe_7dpi_17.czi				
dSarmattPKO_7dpe_7dpi_18				
dSarmattPKO_7dpe_7dpi_19				
dSarmattPKO_7dpe_7dpi_20				
AVG	13.0	13.0	13.3	10.7

dSarmE642A_7dpe_7dpi_1.czi	7.0	13.0	9	7
dSarmE642A_7dpe_7dpi_2.czi	14.0	9.0	3	11
dSarmE642A_7dpe_7dpi_3.czi	9.0	14.0	9	8
dSarmE642A_7dpe_7dpi_4.czi	11.0	8.0	10	4
dSarmE642A_7dpe_7dpi_5.czi	6.0	4.0	6.0	5.0
dSarmE642A_7dpe_7dpi_6.czi	6.0	3.0	6	4
dSarmE642A_7dpe_7dpi_7.czi	9.0	9.0	13	2
dSarmE642A_7dpe_7dpi_8.czi	7.0	9.0	16	13
dSarmE642A_7dpe_7dpi_9.czi	4.0	7.0	8	1
dSarmE642A_7dpe_7dpi_10.czi	11.0	5.0	15	12
dSarmE642A_7dpe_7dpi_11.czi	12.0	10.0		
dSarmE642A_7dpe_7dpi_12.czi	14.0	3.0		
dSarmE642A_7dpe_7dpi_13.czi				
dSarmE642A_7dpe_7dpi_14.czi				
dSarmE642A_7dpe_7dpi_15.czi				
dSarmE642A_7dpe_7dpi_16.czi				
dSarmE642A_7dpe_7dpi_17.czi				
dSarmE642A_7dpe_7dpi_18				
dSarmE642A_7dpe_7dpi_19				
dSarmE642A_7dpe_7dpi_20				
AVG	9.2	7.8	9.5	6.7
dSarm^SAM_7dpe_7dpi_1.czi	18.0	9.0	11	12
dSarm^SAM_7dpe_7dpi_2.czi	14.0	3.0	16.0	16.0
dSarm^SAM_7dpe_7dpi_3.czi	4.0	8.0	2	7
dSarm^SAM_7dpe_7dpi_4.czi	9.0	12.0	22	9
dSarm^SAM_7dpe_7dpi_5.czi	9.0	8.0		
dSarm^SAM_7dpe_7dpi_6.czi	16.0	16.0		
dSarm^SAM_7dpe_7dpi_7.czi	13.0	13.0		
dSarm^SAM_7dpe_7dpi_8.czi				
dSarm^SAM_7dpe_7dpi_9.czi				
dSarm^SAM_7dpe_7dpi_10.czi				
dSarm^SAM_7dpe_7dpi_11.czi				
dSarm^SAM_7dpe_7dpi_12				
dSarm^SAM_7dpe_7dpi_13				
dSarm^SAM_7dpe_7dpi_14				
dSarm^SAM_7dpe_7dpi_15				
dSarm^SAM_7dpe_7dpi_16				
dSarm^SAM_7dpe_7dpi_17				
dSarm^SAM_7dpe_7dpi_18				
dSarm^SAM_7dpe_7dpi_19				
dSarm^SAM_7dpe_7dpi_20				
AVG	11.9	9.9	12.8	11.0
dSarm^TIR_7dpe_7dpi_1.czi	9.0	12.0	8	7

dSarm^TIR_7dpe_7dpi_2.czi	12.0	11.0	14	13
dSarm^TIR_7dpe_7dpi_3.czi	5.0	6.0	14	9
dSarm^TIR_7dpe_7dpi_4.czi	17.0	13.0		
dSarm^TIR_7dpe_7dpi_5	15.0	6.0		
dSarm^TIR_7dpe_7dpi_6	7.0	9.0		
dSarm^TIR_7dpe_7dpi_7	15.0	13.0		
dSarm^TIR_7dpe_7dpi_8	9.0	9.0		
dSarm^TIR_7dpe_7dpi_9	15.0	9.0		
dSarm^TIR_7dpe_7dpi_10	23.0	9.0		
dSarm^TIR_7dpe_7dpi_11	14.0	15.0		
dSarm^TIR_7dpe_7dpi_12	16.0	14.0		
dSarm^TIR_7dpe_7dpi_13				
dSarm^TIR_7dpe_7dpi_14				
dSarm^TIR_7dpe_7dpi_15				
dSarm^TIR_7dpe_7dpi_16				
dSarm^TIR_7dpe_7dpi_17				
dSarm^TIR_7dpe_7dpi_18				
dSarm^TIR_7dpe_7dpi_19				
dSarm^TIR_7dpe_7dpi_20				
AVG	13.1	10.5	12.0	9.7

07/25/21		07/17/21	
Uninjured	Injured	Uninjured	Injured
11	2	9	2
6	0	4	4
14	4	22	4
14	1	16	3
12	0	12	3
18	0	16	0
8	0	12	0
14	0	8	0
15	0	12	0
11	1	15	2
12	0	8	0
9	1	14	0
10	0	15	0
7	0	12	1
7	3	10	2
11.2	0.8	12.3	1.4
16	16	16	2
16	5	4	11
4	9	6	12
12	10	12	2
18	3	16	10
8	7	7	13
6	17	6	17
4	9	9	11
14	2	6	4
6	3	11	13
10.4	8.1	9.3	9.5

#DIV/0!	#DIV/0!		
17.0	15.0	17.0	13.0
13.0	7.0	22.0	11.0
10.0	6.0	12.0	5
14.0	15.0	15.0	9.0
5.0	7.0	12.0	9.0
3.0	14.0	17.0	14.0
13.0	8.0	8.0	11.0
16.0	12.0	20.0	6.0
14.0	8.0	7.0	8.0
14.0	11.0	6.0	9.0
		10.0	11.0
		9.0	8.0
11.9	10.3	12.9	9.5

#DIV/0!	#DIV/0!		
5	11	10	10
15.0	14.0	8.0	11.0
12	17	15	5
12	7	14.0	10
11	16		
10	12		
16	2		
11.6	11.3	11.8	9.0
15	9		

7	10
7	5
11	8
12	13
17	15
20	11
19	9
12	11
7	8
15	10
11	6
12.8	9.6

11	2		
6	0		
14	4		
14	1		
12	0		
18	0		
8	0		
14	0		
15	0		
11	1		
12	0		
9	1		
10	0		
7	0		
7	3		
9	2		
4	4		
22	4		
16	3		
16	3		
16	0		
12	0		
8	0	16	16
18	0	16	5
15	2	4	9
8	0	12	10
14	0	18	3
15	0	8	7
18	1	6	17
10	2	4	9
		14	2
		6	3
		16	2
		4	11
		6	12
		12	2
		16	10
		7	13
		6	17
		9	11
		6	4
		11	13

17.0	15.0
13.0	7.0
10.0	6.0
14.0	15.0
5.0	7.0
3.0	14.0
13.0	8.0
16.0	12.0
14.0	8.0
14.0	11.0
17.0	13.0
22.0	11.0
12.0	5
15.0	9.0
12.0	9.0
17.0	14.0
8.0	11.0
20.0	6.0
7.0	8.0
6.0	9.0
10.0	11.0
9.0	8.0

5	11
15.0	14.0
12	17
12	7
11	16
10	12
16	2
10	10
8.0	11.0
15	5
14.0	10

	03/09/21		03/11/21	
Filename	Uninjured	Injured	Uninjured	Injured
FRT2A82B_7dpe_7dpi_1.czi				
FRT2A82B_7dpe_7dpi_2				
FRT2A82B_7dpe_7dpi_3				
FRT2A82B_7dpe_7dpi_4				
FRT2A82B_7dpe_7dpi_5				
FRT2A82B_7dpe_7dpi_6				
FRT2A82B_7dpe_7dpi_7				
FRT2A82B_7dpe_7dpi_8				
FRT2A82B_7dpe_7dpi_9				
FRT2A82B_7dpe_7dpi_10				
FRT2A82B_7dpe_7dpi_11				
FRT2A82B_7dpe_7dpi_12				
FRT2A82B_7dpe_7dpi_13				
FRT2A82B_7dpe_7dpi_14				
FRT2A82B_7dpe_7dpi_15				
FRT2A82B_7dpe_7dpi_16				
FRT2A82B_7dpe_7dpi_17				
FRT2A82B_7dpe_7dpi_18				
FRT2A82B_7dpe_7dpi_19				
FRT2A82B_7dpe_7dpi_20				
AVG	#DIV/0!	#DIV/0!	#DIV/0!	#DIV/0!
dSarm896_7dpe_7dpi_1.czi				
dSarm896_7dpe_7dpi_2.czi				
dSarm896_7dpe_7dpi_3.czi				
dSarm896_7dpe_7dpi_4.czi				
dSarm896_7dpe_7dpi_5.czi				
dSarm896_7dpe_7dpi_6.czi				
dSarm896_7dpe_7dpi_7				
dSarm896_7dpe_7dpi_8				
dSarm896_7dpe_7dpi_9				
dSarm896_7dpe_7dpi_10				
dSarm896_7dpe_7dpi_11				
dSarm896_7dpe_7dpi_12				
dSarm896_7dpe_7dpi_13				
dSarm896_7dpe_7dpi_14				
dSarm896_7dpe_7dpi_15				
dSarm896_7dpe_7dpi_16				
dSarm896_7dpe_7dpi_17				
dSarm896_7dpe_7dpi_18				
dSarm896_7dpe_7dpi_19				
dSarm896_7dpe_7dpi_20				
AVG	#DIV/0!	#DIV/0!	#DIV/0!	#DIV/0!

dSarmRescue_7dpe_7dpi_1.czi	12.0	0.0	12.0	0.0
dSarmRescue_7dpe_7dpi_2.czi	8.0	0.0	8.0	0.0
dSarmRescue_7dpe_7dpi_3.czi	13.0	0.0	10.0	0.0
dSarmRescue_7dpe_7dpi_4.czi	11.0	0.0	4.0	1.0
dSarmRescue_7dpe_7dpi_5.czi	11.0	0.0		
dSarmRescue_7dpe_7dpi_6.czi	6.0	2.0		
dSarmRescue_7dpe_7dpi_7.czi	11.0	0.0		
dSarmRescue_7dpe_7dpi_8.czi	11.0	1.0		
dSarmRescue_7dpe_7dpi_9.czi				
dSarmRescue_7dpe_7dpi_10.czi				
dSarmRescue_7dpe_7dpi_11.czi				
dSarmRescue_7dpe_7dpi_12.czi				
dSarmRescue_7dpe_7dpi_13.czi				
dSarmRescue_7dpe_7dpi_14.czi				
dSarmRescue_7dpe_7dpi_15.czi				
dSarmRescue_7dpe_7dpi_16.czi				
dSarmRescue_7dpe_7dpi_17				
dSarmRescue_7dpe_7dpi_18				
dSarmRescue_7dpe_7dpi_19				
dSarmRescue_7dpe_7dpi_20				
AVG	10.4	0.4	8.5	0.3
dSarmattPKO_7dpe_7dpi_1.czi	2.0	10.0	14.0	11.0
dSarmattPKO_7dpe_7dpi_2.czi	15.0	9.0	16.0	12.0
dSarmattPKO_7dpe_7dpi_3.czi	11.0	15.0	10.0	7.0
dSarmattPKO_7dpe_7dpi_4.czi	12.0	14.0		
dSarmattPKO_7dpe_7dpi_5.czi	11.0	5.0		
dSarmattPKO_7dpe_7dpi_6.czi	13.0	11.0		
dSarmattPKO_7dpe_7dpi_7.czi				
dSarmattPKO_7dpe_7dpi_8.czi				
dSarmattPKO_7dpe_7dpi_9.czi				
dSarmattPKO_7dpe_7dpi_10.czi				
dSarmattPKO_7dpe_7dpi_11.czi				
dSarmattPKO_7dpe_7dpi_12.czi				
dSarmattPKO_7dpe_7dpi_13.czi				
dSarmattPKO_7dpe_7dpi_14.czi				
dSarmattPKO_7dpe_7dpi_15.czi				
dSarmattPKO_7dpe_7dpi_16.czi				
dSarmattPKO_7dpe_7dpi_17.czi				
dSarmattPKO_7dpe_7dpi_18				
dSarmattPKO_7dpe_7dpi_19				
dSarmattPKO_7dpe_7dpi_20				
AVG	10.7	10.7	13.3	10.0

dSarmE642A_7dpe_7dpi_1.czi	12.0	10.0	12	14
dSarmE642A_7dpe_7dpi_2.czi	6.0	11.0	13.0	11.0
dSarmE642A_7dpe_7dpi_3.czi				
dSarmE642A_7dpe_7dpi_4.czi	12.0	11.0		
dSarmE642A_7dpe_7dpi_5.czi				
dSarmE642A_7dpe_7dpi_6.czi	13.0	10.0		
dSarmE642A_7dpe_7dpi_7.czi	7.0	12.0		
dSarmE642A_7dpe_7dpi_8.czi	9.0	9.0		
dSarmE642A_7dpe_7dpi_9.czi	11.0	0.0		
dSarmE642A_7dpe_7dpi_10.czi				
dSarmE642A_7dpe_7dpi_11.czi				
dSarmE642A_7dpe_7dpi_12.czi				
dSarmE642A_7dpe_7dpi_13.czi				
dSarmE642A_7dpe_7dpi_14.czi				
dSarmE642A_7dpe_7dpi_15.czi				
dSarmE642A_7dpe_7dpi_16.czi				
dSarmE642A_7dpe_7dpi_17.czi				
dSarmE642A_7dpe_7dpi_18				
dSarmE642A_7dpe_7dpi_19				
dSarmE642A_7dpe_7dpi_20				
AVG	10.0	9.0	12.5	12.5
dSarm^SAM_7dpe_7dpi_1.czi				
dSarm^SAM_7dpe_7dpi_2.czi				
dSarm^SAM_7dpe_7dpi_3.czi				
dSarm^SAM_7dpe_7dpi_4.czi				
dSarm^SAM_7dpe_7dpi_5.czi				
dSarm^SAM_7dpe_7dpi_6.czi				
dSarm^SAM_7dpe_7dpi_7.czi				
dSarm^SAM_7dpe_7dpi_8.czi				
dSarm^SAM_7dpe_7dpi_9.czi				
dSarm^SAM_7dpe_7dpi_10.czi				
dSarm^SAM_7dpe_7dpi_11.czi				
dSarm^SAM_7dpe_7dpi_12				
dSarm^SAM_7dpe_7dpi_13				
dSarm^SAM_7dpe_7dpi_14				
dSarm^SAM_7dpe_7dpi_15				
dSarm^SAM_7dpe_7dpi_16				
dSarm^SAM_7dpe_7dpi_17				
dSarm^SAM_7dpe_7dpi_18				
dSarm^SAM_7dpe_7dpi_19				
dSarm^SAM_7dpe_7dpi_20				
AVG	#DIV/0!	#DIV/0!	#DIV/0!	#DIV/0!
dSarm^TIR_7dpe_7dpi_1.czi	9	15	11	9

dSarm^TIR_7dpe_7dpi_2.czi	9	14	8	10
dSarm^TIR_7dpe_7dpi_3.czi	11.0	6.0	15	11
dSarm^TIR_7dpe_7dpi_4.czi	11.0	5.0	11	7
dSarm^TIR_7dpe_7dpi_5	9.0	8.0	6	12
dSarm^TIR_7dpe_7dpi_6	10.0	11.0		
dSarm^TIR_7dpe_7dpi_7	10.0		9	11
dSarm^TIR_7dpe_7dpi_8	15.0	11.0	7	15
dSarm^TIR_7dpe_7dpi_9	14.0	7.0	9	10
dSarm^TIR_7dpe_7dpi_10	16.0	0.0	15	18
dSarm^TIR_7dpe_7dpi_11	13.0	12.0		
dSarm^TIR_7dpe_7dpi_12				
dSarm^TIR_7dpe_7dpi_13				
dSarm^TIR_7dpe_7dpi_14				
dSarm^TIR_7dpe_7dpi_15				
dSarm^TIR_7dpe_7dpi_16				
dSarm^TIR_7dpe_7dpi_17				
dSarm^TIR_7dpe_7dpi_18				
dSarm^TIR_7dpe_7dpi_19				
dSarm^TIR_7dpe_7dpi_20				
AVG	11.5	8.9	10.1	11.4
dSarm^ARM^SAM_1dpe_2dpi_1.czi	13	12	12	9
dSarm^ARM^SAM_1dpe_2dpi_2.czi	7	8		
dSarm^ARM^SAM_1dpe_2dpi_3.czi				
dSarm^ARM^SAM_1dpe_2dpi_4.czi				
dSarm^ARM^SAM_1dpe_2dpi_5.czi				
dSarm^ARM^SAM_1dpe_2dpi_6.czi				
dSarm^ARM^SAM_1dpe_2dpi_7.czi				
dSarm^ARM^SAM_1dpe_2dpi_8.czi				
dSarm^ARM^SAM_1dpe_2dpi_9.czi				
dSarm^ARM^SAM_1dpe_2dpi_10.czi				
dSarm^ARM^SAM_1dpe_2dpi_11.czi				
dSarm^ARM^SAM_1dpe_2dpi_12.czi				
dSarm^ARM^SAM_1dpe_2dpi_13.czi				
dSarm^ARM^SAM_1dpe_2dpi_14.czi				
dSarm^ARM^SAM_1dpe_2dpi_15.czi				
dSarm^ARM^SAM_1dpe_2dpi_16.czi				
dSarm^ARM^SAM_1dpe_2dpi_17				
dSarm^ARM^SAM_1dpe_2dpi_18				
dSarm^ARM^SAM_1dpe_2dpi_19				
dSarm^ARM^SAM_1dpe_2dpi_20				
AVG	10.0	10.0	12.0	#DIV/0!

03/14/21	
Uninjured	Injured
#DIV/0!	#DIV/0!
#DIV/0!	#DIV/0!

9.0	0.0
9.0	0.0
3.0	1.0
10.0	0.0
5.0	0.0
7.0	0.0

7.2	0.2

12.0	13.0
16.0	2.0
7.0	18.0
13.0	5.0
10.0	11.0
10.0	11.0
9.0	5.0
9.0	2.0
12.0	14.0

10.9	9.0

16	13
9.0	12.0
4	4
7.0	10
11	11.0
4.0	14
16	12
15.0	12
11	10
2.0	13
6	8
9.0	9
9.2	10.7

#DIV/0! c

12	15

2	9
8	10
5	8
9	8
4	9
10	15
19	17
13	15

9.1	11.8
6	10

6.0	#DIV/0!

	03/14/21		03/24/21	
Filename	Uninjured	Injured	Uninjured	Injured
FRT2A82B_7dpe_7dpi_1.czi				
FRT2A82B_7dpe_7dpi_2				
FRT2A82B_7dpe_7dpi_3				
FRT2A82B_7dpe_7dpi_4				
FRT2A82B_7dpe_7dpi_5				
FRT2A82B_7dpe_7dpi_6				
FRT2A82B_7dpe_7dpi_7				
FRT2A82B_7dpe_7dpi_8				
FRT2A82B_7dpe_7dpi_9				
FRT2A82B_7dpe_7dpi_10				
FRT2A82B_7dpe_7dpi_11				
FRT2A82B_7dpe_7dpi_12				
FRT2A82B_7dpe_7dpi_13				
FRT2A82B_7dpe_7dpi_14				
FRT2A82B_7dpe_7dpi_15				
FRT2A82B_7dpe_7dpi_16				
FRT2A82B_7dpe_7dpi_17				
FRT2A82B_7dpe_7dpi_18				
FRT2A82B_7dpe_7dpi_19				
FRT2A82B_7dpe_7dpi_20				
AVG	#DIV/0!	#DIV/0!	#DIV/0!	#DIV/0!
dSarm896_7dpe_7dpi_1.czi				
dSarm896_7dpe_7dpi_2.czi				
dSarm896_7dpe_7dpi_3.czi				
dSarm896_7dpe_7dpi_4.czi				
dSarm896_7dpe_7dpi_5.czi				
dSarm896_7dpe_7dpi_6.czi				
dSarm896_7dpe_7dpi_7				
dSarm896_7dpe_7dpi_8				
dSarm896_7dpe_7dpi_9				
dSarm896_7dpe_7dpi_10				
dSarm896_7dpe_7dpi_11				
dSarm896_7dpe_7dpi_12				
dSarm896_7dpe_7dpi_13				
dSarm896_7dpe_7dpi_14				
dSarm896_7dpe_7dpi_15				
dSarm896_7dpe_7dpi_16				
dSarm896_7dpe_7dpi_17				
dSarm896_7dpe_7dpi_18				
dSarm896_7dpe_7dpi_19				
dSarm896_7dpe_7dpi_20				
AVG	#DIV/0!	#DIV/0!	#DIV/0!	#DIV/0!

dSarmRescue_7dpe_7dpi_1.czi	7.0	0.0	17.0	0.0
dSarmRescue_7dpe_7dpi_2.czi	7.0	0.0	2.0	0.0
dSarmRescue_7dpe_7dpi_3.czi	13.0	0.0	8.0	0.0
dSarmRescue_7dpe_7dpi_4.czi	7.0	0.0	6.0	0.0
dSarmRescue_7dpe_7dpi_5.czi	9.0	0.0	5.0	0.0
dSarmRescue_7dpe_7dpi_6.czi	10.0	1.0	8.0	0.0
dSarmRescue_7dpe_7dpi_7.czi	8.0	2.0	6.0	0.0
dSarmRescue_7dpe_7dpi_8.czi	6.0	0.0	6.0	0.0
dSarmRescue_7dpe_7dpi_9.czi	7.0	3.0	6.0	1.0
dSarmRescue_7dpe_7dpi_10.czi	5.0	0.0	10.0	0.0
dSarmRescue_7dpe_7dpi_11.czi	4.0	0.0	12.0	0.0
dSarmRescue_7dpe_7dpi_12.czi				
dSarmRescue_7dpe_7dpi_13.czi				
dSarmRescue_7dpe_7dpi_14.czi				
dSarmRescue_7dpe_7dpi_15.czi				
dSarmRescue_7dpe_7dpi_16.czi				
dSarmRescue_7dpe_7dpi_17				
dSarmRescue_7dpe_7dpi_18				
dSarmRescue_7dpe_7dpi_19				
dSarmRescue_7dpe_7dpi_20				
AVG	7.5	0.5	7.8	0.1
dSarmattPKO_7dpe_7dpi_1.czi			4.0	12.0
dSarmattPKO_7dpe_7dpi_2.czi			8.0	6.0
dSarmattPKO_7dpe_7dpi_3.czi			11.0	12.0
dSarmattPKO_7dpe_7dpi_4.czi			8.0	10.0
dSarmattPKO_7dpe_7dpi_5.czi			13.0	9.0
dSarmattPKO_7dpe_7dpi_6.czi				
dSarmattPKO_7dpe_7dpi_7.czi				
dSarmattPKO_7dpe_7dpi_8.czi				
dSarmattPKO_7dpe_7dpi_9.czi				
dSarmattPKO_7dpe_7dpi_10.czi				
dSarmattPKO_7dpe_7dpi_11.czi				
dSarmattPKO_7dpe_7dpi_12.czi				
dSarmattPKO_7dpe_7dpi_13.czi				
dSarmattPKO_7dpe_7dpi_14.czi				
dSarmattPKO_7dpe_7dpi_15.czi				
dSarmattPKO_7dpe_7dpi_16.czi				
dSarmattPKO_7dpe_7dpi_17.czi				
dSarmattPKO_7dpe_7dpi_18				
dSarmattPKO_7dpe_7dpi_19				
dSarmattPKO_7dpe_7dpi_20				
AVG	#DIV/0!	#DIV/0!	8.8	9.8

dSarmE642A_7dpe_7dpi_1.czi	8.0	12.0	14	11
dSarmE642A_7dpe_7dpi_2.czi	11.0	6.0	14.0	5.0
dSarmE642A_7dpe_7dpi_3.czi			7	22
dSarmE642A_7dpe_7dpi_4.czi	4	9.0	8	10
dSarmE642A_7dpe_7dpi_5.czi	11.0	14.0	9.0	10.0
dSarmE642A_7dpe_7dpi_6.czi	6.0	9.0	11	8
dSarmE642A_7dpe_7dpi_7.czi	3.0	15.0	6	7
dSarmE642A_7dpe_7dpi_8.czi				
dSarmE642A_7dpe_7dpi_9.czi				
dSarmE642A_7dpe_7dpi_10.czi				
dSarmE642A_7dpe_7dpi_11.czi				
dSarmE642A_7dpe_7dpi_12.czi				
dSarmE642A_7dpe_7dpi_13.czi				
dSarmE642A_7dpe_7dpi_14.czi				
dSarmE642A_7dpe_7dpi_15.czi				
dSarmE642A_7dpe_7dpi_16.czi				
dSarmE642A_7dpe_7dpi_17.czi				
dSarmE642A_7dpe_7dpi_18				
dSarmE642A_7dpe_7dpi_19				
dSarmE642A_7dpe_7dpi_20				
AVG	7.2	10.8	9.9	10.4
dSarm^SAM_7dpe_7dpi_1.czi	11.0	17.0		
dSarm^SAM_7dpe_7dpi_2.czi				
dSarm^SAM_7dpe_7dpi_3.czi				
dSarm^SAM_7dpe_7dpi_4.czi				
dSarm^SAM_7dpe_7dpi_5.czi				
dSarm^SAM_7dpe_7dpi_6.czi				
dSarm^SAM_7dpe_7dpi_7.czi				
dSarm^SAM_7dpe_7dpi_8.czi				
dSarm^SAM_7dpe_7dpi_9.czi				
dSarm^SAM_7dpe_7dpi_10.czi				
dSarm^SAM_7dpe_7dpi_11.czi				
dSarm^SAM_7dpe_7dpi_12				
dSarm^SAM_7dpe_7dpi_13				
dSarm^SAM_7dpe_7dpi_14				
dSarm^SAM_7dpe_7dpi_15				
dSarm^SAM_7dpe_7dpi_16				
dSarm^SAM_7dpe_7dpi_17				
dSarm^SAM_7dpe_7dpi_18				
dSarm^SAM_7dpe_7dpi_19				
dSarm^SAM_7dpe_7dpi_20				
AVG	#DIV/0!	#DIV/0!	#DIV/0!	#DIV/0!
dSarm^TIR_7dpe_7dpi_1.czi	8.0	9.0	15	7

dSarm^TIR_7dpe_7dpi_2.czi	9.0	8.0	9	10
dSarm^TIR_7dpe_7dpi_3.czi	8.0	10.0	8	8
dSarm^TIR_7dpe_7dpi_4.czi	10.0	9.0	15	5
dSarm^TIR_7dpe_7dpi_5	11.0	9.0	11	12
dSarm^TIR_7dpe_7dpi_6	13.0	0.0	18	8
dSarm^TIR_7dpe_7dpi_7	2.0	12.0	6	12
dSarm^TIR_7dpe_7dpi_8	4.0	14.0	7	9
dSarm^TIR_7dpe_7dpi_9			13	0
dSarm^TIR_7dpe_7dpi_10	14.0	5.0	15	13
dSarm^TIR_7dpe_7dpi_11	14.0	11.0	13	5
dSarm^TIR_7dpe_7dpi_12	3.0	7.0	3	5
dSarm^TIR_7dpe_7dpi_13	9.0	13.0	6	12
dSarm^TIR_7dpe_7dpi_14	10.0	5.0	10	10
dSarm^TIR_7dpe_7dpi_15				
dSarm^TIR_7dpe_7dpi_16				
dSarm^TIR_7dpe_7dpi_17				
dSarm^TIR_7dpe_7dpi_18				
dSarm^TIR_7dpe_7dpi_19				
dSarm^TIR_7dpe_7dpi_20				
AVG	8.8	8.6	10.6	8.3
dSarm^ARM^SAM_1dpe_2dpi_1.czi	7	11		
dSarm^ARM^SAM_1dpe_2dpi_2.czi				
dSarm^ARM^SAM_1dpe_2dpi_3.czi	7	0		
dSarm^ARM^SAM_1dpe_2dpi_4.czi	8	18		
dSarm^ARM^SAM_1dpe_2dpi_5.czi				
dSarm^ARM^SAM_1dpe_2dpi_6.czi				
dSarm^ARM^SAM_1dpe_2dpi_7.czi				
dSarm^ARM^SAM_1dpe_2dpi_8.czi				
dSarm^ARM^SAM_1dpe_2dpi_9.czi				
dSarm^ARM^SAM_1dpe_2dpi_10.czi				
dSarm^ARM^SAM_1dpe_2dpi_11.czi				
dSarm^ARM^SAM_1dpe_2dpi_12.czi				
dSarm^ARM^SAM_1dpe_2dpi_13.czi				
dSarm^ARM^SAM_1dpe_2dpi_14.czi				
dSarm^ARM^SAM_1dpe_2dpi_15.czi				
dSarm^ARM^SAM_1dpe_2dpi_16.czi				
dSarm^ARM^SAM_1dpe_2dpi_17				
dSarm^ARM^SAM_1dpe_2dpi_18				
dSarm^ARM^SAM_1dpe_2dpi_19				
dSarm^ARM^SAM_1dpe_2dpi_20				
AVG	7.3	9.7	#DIV/0!	#DIV/0!

03/24b/21	
Uninjured	Injured
#DIV/0!	#DIV/0!
#DIV/0!	#DIV/0!

				7.0	0.0
9.0	0.0			7.0	0.0
8.0	0.0			13.0	0.0
11.0	0.0			7.0	0.0
12.0	0.0			9.0	0.0
13.0	2.0			10.0	1.0
13.0	3.0			8.0	2.0
8.0	0.0			6.0	0.0
6.0	0.0			7.0	3.0
9.0	0.0			5.0	0.0
3.0	0.0			4.0	0.0
14.0	0.0			17.0	0.0
13.0	0.0			2.0	0.0
				8.0	0.0
				6.0	0.0
				5.0	0.0
				8.0	0.0
				6.0	0.0
				6.0	0.0
				6.0	1.0
				10.0	0.0
9.9	0.4			12.0	0.0
				9.0	0.0
14.0	4.0			8.0	0.0
13.0	14.0			11.0	0.0
6.0	11.0			12.0	0.0
				13.0	2.0
				13.0	3.0
				8.0	0.0
		4.0	12.0	6.0	0.0
		8.0	6.0	9.0	0.0
		11.0	12.0	3.0	0.0
		8.0	10.0	14.0	0.0
		13.0	9.0	13.0	0.0
		14.0	4.0		
		13.0	14.0		
		6.0	11.0		
11.0	9.7				

2	11		
7.0	18.0	8.0	12.0
7	5	11.0	6.0
8	8	4	9.0
11.0	5.0	11.0	14.0
7	7	6.0	9.0
10	8	3.0	15.0
11	5	14	11
1	11	14.0	5.0
23	1	7	22
5	1	8	10
		9.0	10.0
		11	8
		6	7
		2	11
		7.0	18.0
		7	5
		8	8
		11.0	5.0
		7	7
8.4	7.3	10	8
		11	5
		1	11
		23	1
		5	1
		8.0	9.0
		9.0	8.0
		8.0	10.0
		10.0	9.0
		11.0	9.0
		13.0	0.0
		2.0	12.0
		4.0	14.0
		14.0	5.0
		14.0	11.0
		3.0	7.0
		9.0	13.0
		10.0	5.0
		15	7
#DIV/0!	#DIV/0!	9	10
		8	8
7	10	15	5

7	7	11	12	
11	5	18	8	
7	23	6	12	
16	6	7	9	
14	1	13	0	
11	12	15	13	
11	6	13	5	
8	11	3	5	
		6	12	
		10	10	
		7	10	
		7	7	
		11	5	
		7	23	
		16	6	
		14	1	
		11	12	
		11	6	
		8	11	
10.2	9.0			
12	13		7	11
			7	0
			8	18
			12	13
12.0	13.0			

	02/13/21			02/18/21	
Filename	Uninjured	Injured		Uninjured	Injured
FRT2A82B_7dpe_7dpi_1.czi					
FRT2A82B_7dpe_7dpi_2					
FRT2A82B_7dpe_7dpi_3					
FRT2A82B_7dpe_7dpi_4					
FRT2A82B_7dpe_7dpi_5					
FRT2A82B_7dpe_7dpi_6					
FRT2A82B_7dpe_7dpi_7					
FRT2A82B_7dpe_7dpi_8					
FRT2A82B_7dpe_7dpi_9					
FRT2A82B_7dpe_7dpi_10					
FRT2A82B_7dpe_7dpi_11					
FRT2A82B_7dpe_7dpi_12					
FRT2A82B_7dpe_7dpi_13					
FRT2A82B_7dpe_7dpi_14					
FRT2A82B_7dpe_7dpi_15					
FRT2A82B_7dpe_7dpi_16					
FRT2A82B_7dpe_7dpi_17					
FRT2A82B_7dpe_7dpi_18					
FRT2A82B_7dpe_7dpi_19					
FRT2A82B_7dpe_7dpi_20					
AVG	#DIV/0!	#DIV/0!		#DIV/0!	#DIV/0!
dSarm896_7dpe_7dpi_1.czi					
dSarm896_7dpe_7dpi_2.czi					
dSarm896_7dpe_7dpi_3.czi					
dSarm896_7dpe_7dpi_4.czi					
dSarm896_7dpe_7dpi_5.czi					
dSarm896_7dpe_7dpi_6.czi					
dSarm896_7dpe_7dpi_7					
dSarm896_7dpe_7dpi_8					
dSarm896_7dpe_7dpi_9					
dSarm896_7dpe_7dpi_10					
dSarm896_7dpe_7dpi_11					
dSarm896_7dpe_7dpi_12					
dSarm896_7dpe_7dpi_13					
dSarm896_7dpe_7dpi_14					
dSarm896_7dpe_7dpi_15					
dSarm896_7dpe_7dpi_16					
dSarm896_7dpe_7dpi_17					
dSarm896_7dpe_7dpi_18					
dSarm896_7dpe_7dpi_19					
dSarm896_7dpe_7dpi_20					
AVG	#DIV/0!	#DIV/0!		#DIV/0!	#DIV/0!

dSarmRescue_7dpe_7dpi_1.czi					
dSarmRescue_7dpe_7dpi_2.czi					
dSarmRescue_7dpe_7dpi_3.czi					
dSarmRescue_7dpe_7dpi_4.czi					
dSarmRescue_7dpe_7dpi_5.czi					
dSarmRescue_7dpe_7dpi_6.czi					
dSarmRescue_7dpe_7dpi_7.czi					
dSarmRescue_7dpe_7dpi_8.czi					
dSarmRescue_7dpe_7dpi_9.czi					
dSarmRescue_7dpe_7dpi_10.czi					
dSarmRescue_7dpe_7dpi_11.czi					
dSarmRescue_7dpe_7dpi_12.czi					
dSarmRescue_7dpe_7dpi_13.czi					
dSarmRescue_7dpe_7dpi_14.czi					
dSarmRescue_7dpe_7dpi_15.czi					
dSarmRescue_7dpe_7dpi_16.czi					
dSarmRescue_7dpe_7dpi_17					
dSarmRescue_7dpe_7dpi_18					
dSarmRescue_7dpe_7dpi_19					
dSarmRescue_7dpe_7dpi_20					
AVG	#DIV/0!	#DIV/0!		#DIV/0!	#DIV/0!

dSarmattPKO_7dpe_7dpi_1.czi					
dSarmattPKO_7dpe_7dpi_2.czi					
dSarmattPKO_7dpe_7dpi_3.czi					
dSarmattPKO_7dpe_7dpi_4.czi					
dSarmattPKO_7dpe_7dpi_5.czi					
dSarmattPKO_7dpe_7dpi_6.czi					
dSarmattPKO_7dpe_7dpi_7.czi					
dSarmattPKO_7dpe_7dpi_8.czi					
dSarmattPKO_7dpe_7dpi_9.czi					
dSarmattPKO_7dpe_7dpi_10.czi					
dSarmattPKO_7dpe_7dpi_11.czi					
dSarmattPKO_7dpe_7dpi_12.czi					
dSarmattPKO_7dpe_7dpi_13.czi					
dSarmattPKO_7dpe_7dpi_14.czi					
dSarmattPKO_7dpe_7dpi_15.czi					
dSarmattPKO_7dpe_7dpi_16.czi					
dSarmattPKO_7dpe_7dpi_17.czi					
dSarmattPKO_7dpe_7dpi_18					
dSarmattPKO_7dpe_7dpi_19					
dSarmattPKO_7dpe_7dpi_20					
AVG	#DIV/0!	#DIV/0!		#DIV/0!	#DIV/0!

dSarmE642A_7dpe_7dpi_1.czi				
dSarmE642A_7dpe_7dpi_2.czi				
dSarmE642A_7dpe_7dpi_3.czi				
dSarmE642A_7dpe_7dpi_4.czi				
dSarmE642A_7dpe_7dpi_5.czi				
dSarmE642A_7dpe_7dpi_6.czi				
dSarmE642A_7dpe_7dpi_7.czi				
dSarmE642A_7dpe_7dpi_8.czi				
dSarmE642A_7dpe_7dpi_9.czi				
dSarmE642A_7dpe_7dpi_10.czi				
dSarmE642A_7dpe_7dpi_11.czi				
dSarmE642A_7dpe_7dpi_12.czi				
dSarmE642A_7dpe_7dpi_13.czi				
dSarmE642A_7dpe_7dpi_14.czi				
dSarmE642A_7dpe_7dpi_15.czi				
dSarmE642A_7dpe_7dpi_16.czi				
dSarmE642A_7dpe_7dpi_17.czi				
dSarmE642A_7dpe_7dpi_18				
dSarmE642A_7dpe_7dpi_19				
dSarmE642A_7dpe_7dpi_20				
AVG	#DIV/0!	#DIV/0!	#DIV/0!	#DIV/0!
dSarm^SAM_7dpe_7dpi_1.czi				
dSarm^SAM_7dpe_7dpi_2.czi				
dSarm^SAM_7dpe_7dpi_3.czi				
dSarm^SAM_7dpe_7dpi_4.czi				
dSarm^SAM_7dpe_7dpi_5.czi				
dSarm^SAM_7dpe_7dpi_6.czi				
dSarm^SAM_7dpe_7dpi_7.czi				
dSarm^SAM_7dpe_7dpi_8.czi				
dSarm^SAM_7dpe_7dpi_9.czi				
dSarm^SAM_7dpe_7dpi_10.czi				
dSarm^SAM_7dpe_7dpi_11.czi				
dSarm^SAM_7dpe_7dpi_12				
dSarm^SAM_7dpe_7dpi_13				
dSarm^SAM_7dpe_7dpi_14				
dSarm^SAM_7dpe_7dpi_15				
dSarm^SAM_7dpe_7dpi_16				
dSarm^SAM_7dpe_7dpi_17				
dSarm^SAM_7dpe_7dpi_18				
dSarm^SAM_7dpe_7dpi_19				
dSarm^SAM_7dpe_7dpi_20				
AVG	#DIV/0!	#DIV/0!	#DIV/0!	#DIV/0!
dSarm^TIR_7dpe_7dpi_1.czi				

Slide	Count	Total Area	Average Size	% Area
MAX_dSarmRescue_7dpe_1dpi_1.tif	170	35.182	0.207	4.665
MAX_dSarmRescue_7dpe_1dpi_2.tif	118	19.951	0.169	1.868
MAX_dSarmRescue_7dpe_1dpi_3.tif	193	36.909	0.191	3.94
MAX_dSarmRescue_7dpe_1dpi_4.tif	84	27.663	0.329	5.643
MAX_dSarmRescue_7dpe_1dpi_5.tif	238	88.83	0.373	5.144
MAX_dSarmRescue_7dpe_1dpi_6.tif	230	96.81	0.421	13.324
MAX_dSarmRescue_7dpe_1dpi_7.tif	108	24.257	0.225	4.807
MAX_dSarmRescue_7dpe_1dpi_8.tif	189	39.05	0.207	4.84
MAX_dSarmRescue_7dpe_1dpi_9.tif	106	22.724	0.214	5.334
MAX_dSarmRescue_7dpe_1dpi_10.tif	248	69.365	0.28	7.738
AVG	168.4	46.0741	0.2616	5.7303
MAX_dSarm^TIR_7dpe_1dpi_1.tif	226	102.601	0.454	10.341
MAX_dSarm^TIR_7dpe_1dpi_2.tif	231	218.583	0.946	24.274
MAX_dSarm^TIR_7dpe_1dpi_3.tif	185	110.897	0.599	12.378
MAX_dSarm^TIR_7dpe_1dpi_4.tif	363	81.725	0.225	5.725
MAX_dSarm^TIR_7dpe_1dpi_5.tif	176	82.406	0.468	14.626
MAX_dSarm^TIR_7dpe_1dpi_6.tif	391	128.756	0.329	11.236
MAX_dSarm^TIR_7dpe_1dpi_7.tif	158	80.168	0.507	15.941
MAX_dSarm^TIR_7dpe_1dpi_8.tif	323	126.347	0.391	13.642
MAX_dSarm^TIR_7dpe_1dpi_9.tif	333	143.062	0.43	21.641
MAX_dSarm^TIR_7dpe_1dpi_10.tif	340	181.066	0.533	10.685
MAX_dSarm^TIR_7dpe_1dpi_11.tif	14		137.511	226.242
MAX_dSarm^TIR_7dpe_1dpi_12.tif	178	211.235	1.187	24.663
MAX_dSarm^TIR_7dpe_1dpi_13.tif	261	142.429	0.546	13.295
AVG	244.538462	134.10625	11.08661538	31.1299231
MAX_dSarmattPKO_7dpe_1dpi_1.tif	198	123.987	0.626	13.216
MAX_dSarmattPKO_7dpe_1dpi_2.tif	340	167.489	0.493	10.352
MAX_dSarmattPKO_7dpe_1dpi_3.tif	111	32.067	0.289	4.544
MAX_dSarmattPKO_7dpe_1dpi_4.tif	297	160.19	0.539	18.322
MAX_dSarmattPKO_7dpe_1dpi_5.tif	239	73.088	0.306	9.083
MAX_dSarmattPKO_7dpe_1dpi_7.tif	105	75.132	0.716	15.464
MAX_dSarmattPKO_7dpe_1dpi_8.tif	341	74.499	0.218	5.152
MAX_dSarmattPKO_7dpe_1dpi_9.tif	88	31.921	0.363	3.993
AVG	214.875	92.296625	0.44375	10.01575

Mean	Perimeter	IntDen
77.92	1.455	22.998
110.585	1.257	21.755
106.9	1.328	26.593
112.305	1.975	48.178
102.1	2.053	48.27
111.227	2.006	64.484
113.627	1.545	32.019
119.256	1.369	32.041
84.183	1.418	25.345
101.744	1.747	34.946
103.9847	1.6153	35.6629
84.985	2.179	51.268
114.398	3.406	144.765
104.046	2.588	84.677
126.458	1.468	35.395
89.233	2.416	53.326
116.724	1.872	47.87
112.088	2.449	75.115
105.324	1.823	56.328
99.073	2.092	55.95
95.074	2.278	72.009
113.973	21.071	3883.857
104.107	3.085	188.097
100.076	2.378	73.524
105.043	3.77730769	370.937
110.904	2.482	94.853
95.558	2.418	70.912
113.596	1.645	42.432
99.82	1.842	76.621
105.31	1.938	39.164
110.797	2.987	110.707
104.277	1.419	28.312
119.224	1.957	56.747
107.43575	2.086	64.9685

Slide	Count	Total Area	Average Size	% Area
MAX_dSarmRescue_7dpe_1dpi_1.tif	113	49.171	0.435	2.369
MAX_dSarmRescue_7dpe_1dpi_2.tif	104	27.007	0.26	0.793
MAX_dSarmRescue_7dpe_1dpi_3.tif	122	32.675	0.268	0.764
MAX_dSarmRescue_7dpe_1dpi_4.tif	126	34.525	0.274	0.557
MAX_dSarmRescue_7dpe_1dpi_5.tif	61	16.569	0.272	0.433
MAX_dSarmRescue_7dpe_1dpi_6.tif	63	12.335	0.196	0.593
MAX_dSarmRescue_7dpe_1dpi_7.tif	125	30.899	0.247	0.807
MAX_dSarmRescue_7dpe_1dpi_8.tif	163	38.393	0.236	2.758
MAX_dSarmRescue_7dpe_1dpi_9.tif	266	75.18	0.283	3.241
MAX_dSarmRescue_7dpe_1dpi_10.ti	72	15.669	0.218	0.681
MAX_dSarmRescue_7dpe_1dpi_11.ti	144	51.531	0.358	1.927
MAX_dSarmRescue_7dpe_1dpi_12.ti	95	21.484	0.226	0.842
MAX_dSarmRescue_7dpe_1dpi_13.ti	106	37.59	0.355	1.35
MAX_dSarmRescue_7dpe_1dpi_14.ti	122	29.367	0.241	0.555
MAX_dSarmRescue_7dpe_1dpi_15.ti	229	109.486	0.478	2.694
MAX_dSarmRescue_7dpe_1dpi_16.ti	185	39.877	0.216	1.038
AVG	131	38.859875	0.2851875	1.337625
MAX_dSarmattPKO_7dpe_1dpi_1.tif	102	30.072	0.295	1.326
MAX_dSarmattPKO_7dpe_1dpi_2.tif	224	39.78	0.178	1.298
MAX_dSarmattPKO_7dpe_1dpi_3.tif	311	78.66	0.253	2.851
MAX_dSarmattPKO_7dpe_1dpi_4.tif	82	21.386	0.261	0.927
MAX_dSarmattPKO_7dpe_1dpi_5.tif	262	85.715	0.327	4.392
MAX_dSarmattPKO_7dpe_1dpi_6.tif	162	30.121	0.186	1.026
MAX_dSarmattPKO_7dpe_1dpi_7.tif	288	40.096	0.139	2.279
MAX_dSarmattPKO_7dpe_1dpi_8.tif	26	6.91	0.266	0.366
MAX_dSarmattPKO_7dpe_1dpi_9.tif	166	29.634	0.179	1.339
MAX_dSarmattPKO_7dpe_1dpi_10.ti	198	25.887	0.131	0.929
MAX_dSarmattPKO_7dpe_1dpi_11.ti	129	30.559	0.237	1.173
MAX_dSarmattPKO_7dpe_1dpi_12.ti	183	40.364	0.221	1.017
MAX_dSarmattPKO_7dpe_1dpi_13.ti	51	9.294	0.182	0.436
MAX_dSarmattPKO_7dpe_1dpi_14.ti	89	11.216	0.126	0.499
MAX_dSarmattPKO_7dpe_1dpi_15.ti	151	49.804	0.33	2.456
AVG	161.6	35.2998667	0.220733333	1.4876
MAX_dSarm^TIR_7dpe_1dpi_1.tif	173	97.029	0.561	5.062
MAX_dSarm^TIR_7dpe_1dpi_2.tif	194	87.151	0.449	3.978
MAX_dSarm^TIR_7dpe_1dpi_3.tif	428	101.189	0.236	3.881
MAX_dSarm^TIR_7dpe_1dpi_4.tif	294	62.31	0.212	2.252
MAX_dSarm^TIR_7dpe_1dpi_5.tif	155	50.364	0.325	2.616
MAX_dSarm^TIR_7dpe_1dpi_6.tif	165	36.13	0.219	1.578
MAX_dSarm^TIR_7dpe_1dpi_7.tif	165	34.865	0.211	0.815
MAX_dSarm^TIR_7dpe_1dpi_8.tif	173	125.812	0.727	5.314
MAX_dSarm^TIR_7dpe_1dpi_9.tif	135	26.447	0.196	2.025

MAX_dSarm^TIR_7dpe_1dpi_10.tif	315	115.593	0.367	2.091
MAX_dSarm^TIR_7dpe_1dpi_11.tif	133	59.268	0.446	2.66
MAX_dSarm^TIR_7dpe_1dpi_12.tif	118	39.293	0.333	1.445
MAX_dSarm^TIR_7dpe_1dpi_13.tif	276	175.032	0.634	5.922
MAX_dSarm^TIR_7dpe_1dpi_13.tif	231	77.297	0.335	4.043
MAX_dSarm^TIR_7dpe_1dpi_14.tif	227	81.166	0.358	3.316
AVG	212.133333	77.9297333	0.373933333	3.1332

Mean	Perimeter	IntDen
96.373	2.187	58.916
124.866	1.701	40.658
128.351	1.574	45.678
117.403	1.545	41.698
104.841	1.516	42.53
88.301	1.348	23.371
	1.565	10201.426
85.493	1.574	26.074
91.904	1.677	33.776
115.293	1.474	31.243
103.813	1.709	57.985
96.261	1.462	31.991
100.549	1.662	56.408
121.107	1.52	36.74
110.478	2.053	78.857
110.883	1.536	28.796
106.3944	1.6314375	677.259188
113.681	1.669	49.558
81.817	1.261	20.865
91.771	1.53	32.644
87.168	1.473	33.619
92.859	1.958	38.23
92.277	1.289	23.485
57.955	1.125	10.204
102.208	1.636	34.458
81.308	1.278	20.875
78.298	1.012	14.645
127.582	1.461	39.106
69.498	1.594	19.559
125.388	1.356	28.597
71.937	1.088	11.891
86.131	1.769	43.924
90.6585333	1.43326667	28.1106667
107.464	2.937	76.245
118.461	2.234	69.305
83.332	1.435	26.115
69.596	1.427	19.652
78.719	2.013	33.436
125.723	1.549	32.427
84.32	1.295	27.193
98.015	2.845	96.917
108.681	1.396	26.404

121.496	1.917	58.327
112.132	2.437	62.455
118.331	1.79	53.478
121.401	2.444	110.687
120.273	2.022	49.702
88.734	2.037	41.147
103.778533	1.9852	52.2326667

Slide	Count	Total Area	Average Size	% Area
MAX_dSarmE642A_7dpe_1dpi_11.tif	209	34.817	0.167	0.136
MAX_dSarmE642A_7dpe_1dpi_10.tif	476	109.875	0.231	5.506
MAX_dSarmE642A_7dpe_1dpi_9.tif	289	42.627	0.147	0.167
MAX_dSarmE642A_7dpe_1dpi_8.tif	166	22.141	0.133	0.087
MAX_dSarmE642A_7dpe_1dpi_7.tif	266	48.806	0.183	0.191
MAX_dSarmE642A_7dpe_1dpi_5.tif	245	52.48	0.214	0.206
MAX_dSarmE642A_7dpe_1dpi_4.tif	382	54.938	0.144	0.215
MAX_dSarmE642A_7dpe_1dpi_3.tif	403	45.935	0.114	0.18
MAX_dSarmE642A_7dpe_1dpi_2.tif	1122	104.839	0.093	0.411
MAX_dSarmE642A_7dpe_1dpi_1.tif	748	48.295	0.065	0.189
MAX_dSarmattPKO_7dpe_1dpi_5.tif	243	41.824	0.172	0.164
MAX_dSarmattPKO_7dpe_1dpi_6.tif	242	41.386	0.171	0.162
MAX_dSarmattPKO_7dpe_1dpi_7.tif	160	57.711	0.361	0.226
MAX_dSarmattPKO_7dpe_1dpi_8.tif	259	78.781	0.304	0.309
MAX_dSarmattPKO_7dpe_1dpi_9.tif	443	58.198	0.131	0.228
MAX_dSarmattPKO_7dpe_1dpi_10.tif	216	31.143	0.144	0.122
MAX_dSarmattPKO_7dpe_1dpi_11.tif	397	45.303	0.114	0.178
MAX_dSarmattPKO_7dpe_1dpi_12.tif	771	71.069	0.092	0.279

Mean	Perimeter	IntDen
21470.112	1.343	4330.596
90.112	1.41	29.06
19448.073	1.114	4109.407
19475.233	1.139	3408.696
19714.009	1.343	4510.881
21122.372	1.483	5740.247
20108.303	1.119	3728.438
19051.664	0.998	2911.849
16559.048	0.881	2014.951
15924.713	0.747	1259.373
24006.72	1.258	5388.329
22940.899	1.178	5502.877
23371.118	1.895	11241.782
26873.415	1.375	12450.134
20903.763	1.038	3852.745
18334.224	1.117	3678.631
18960.683	1	2796.796
18490.812	0.857	2259.601

dSARM rescue / +	dSARM KO / +	dSARM E1170A / +	dSARM ARM-SAM / +
35.182	123.987	36.666	102.601
19.951	167.489	155.665	218.583
36.909	32.067	105.642	110.897
27.663	160.19	98.221	81.725
88.83	73.088	71.653	82.406
96.81	75.132	41.313	128.756
24.257	74.499	105.545	80.168
39.05	31.921	40.461	126.347
22.724	30.072	52.991	143.062
69.365	39.78	52.748	181.066
65.57	78.66	26.788	211.235
50.291	21.386	23.284	142.429
90.703	85.715	34.817	125.276
54.865	30.121	109.875	44.013
54.524	40.096	42.627	31.508
49.536	6.91	22.141	52.286
75.302	29.634	48.806	97.029
81.36	25.887	52.48	87.151
24.622	30.559	54.938	101.189
57.395	40.364	45.935	62.31
59.682	9.294	104.839	50.364
49.171	11.216	48.295	36.13
27.007	49.804	54.913	34.865
32.675	41.824	32.116	125.812
34.525	41.386	64.134	26.447
16.569	57.711	46.69	115.593
12.335	78.781	63.964	59.268
30.899	58.198	125.69	39.293
38.393	31.143	18.71	175.032
75.18	45.303	52.48	77.297
15.669	71.069	20.291	81.166
51.531			
21.484			
37.59			
29.367			
109.486			
39.877			

dSARM rescue / +	dSARM KO / +	dSARM E1170A / +	dSARM ARM-SAM / +	dSARM rescue / +	dSARM KO / +
35.182	123.987	36.666	102.601	0.745403124	2.626919935
19.951	167.489	155.665	218.583	0.422703022	3.548599394
36.909	32.067	105.642	110.897	0.781993176	0.679405434
27.663	160.19	98.221	81.725	0.586097625	3.393955047
88.83	73.088	71.653	82.406	1.882046488	1.548519798
96.81	75.132	41.313	128.756	2.051119222	1.591826148
24.257	74.499	105.545	80.168	0.5139345	1.578414739
39.05	31.921	40.461	126.347	0.82735467	0.676312123
22.724	30.072	52.991	143.062	0.481454738	0.637137251
69.365	39.78	52.748	181.066	1.469640376	0.842821223
65.57	78.66	26.788	211.235	1.389235486	1.666574093
50.291	21.386	23.284	142.429	1.065518405	0.453106453
90.703	85.715	34.817	125.276	1.92172985	1.816048797
54.865	30.121	109.875	44.013	1.162428015	0.638175417
54.524	40.096	42.627	31.508	1.155203227	0.849516334
49.536	6.91	22.141	52.286	1.049522175	0.146402581
75.302	29.634	48.806	97.029	1.595427948	0.627857319
81.36	25.887	52.48	87.151	1.723779154	0.548469407
24.622	30.559	54.938	101.189	0.521667777	0.647455349
57.395	40.364	45.935	62.31	1.216031275	0.855194466
59.682	9.294	104.839	50.364	1.26448608	0.19691253
49.171	11.216	48.295	36.13	1.041788898	0.237634058
27.007	49.804	54.913	34.865	0.572198914	1.055200307
32.675	41.824	32.116	125.812	0.692287166	0.886127573
34.525	41.386	64.134	26.447	0.731483226	0.876847641
16.569	57.711	46.69	115.593	0.351048387	1.222726386
12.335	78.781	63.964	59.268	0.261342378	1.669137728
30.899	58.198	125.69	39.293	0.654658949	1.233044484
38.393	31.143	18.71	175.032	0.813434772	0.659828591
75.18	45.303	52.48	77.297	1.592843127	0.959837353
15.669	71.069	20.291	81.166	0.331980034	1.505743125
51.531				1.091790359	
21.484				0.455182784	
37.59				0.796421563	
29.367				0.622200374	
109.486				2.319686387	
39.877				0.844876368	
47.1986216				1	1.157282293

dSARM E1170A / +	dSARM ARM-SAM / +
0.77684472	2.173813483
3.298083603	4.631131007
2.23824333	2.349581328
2.081014162	1.73151243
1.518116368	1.745940818
0.875300985	2.727961022
2.236188186	1.698524179
0.857249611	2.676921396
1.122723465	3.031063094
1.11757501	3.836256099
0.567558948	4.475448495
0.493319491	3.017651685
0.737669848	2.654230055
2.327928153	0.932506046
0.903140782	0.667561868
0.469102682	1.107786588
1.034055622	2.055759188
1.111896878	1.846473415
1.16397467	2.143897355
0.973227574	1.320165672
2.221230122	1.06706506
1.023229034	0.765488457
1.163444993	0.738686826
0.6804436	2.665586319
1.358810868	0.56033416
0.989223804	2.449075759
1.355209068	1.255714637
2.663001497	0.832503125
0.396409882	3.708413383
1.111896878	1.637696131
0.429906623	1.719668864
1.267613563	2.071755418

Name	Count	Total Area	Average Size	% Area	Mean
MAX_dSarm^TIR_7dpe_2dpi_1.tif	188	16.131	0.086	100	255
MAX_dSarm^TIR_7dpe_2dpi_2.tif	350	38.174	0.109	100	66.89
MAX_dSarm^TIR_7dpe_2dpi_3.tif	50	34.135	0.683	100	112.2
MAX_dSarm^TIR_7dpe_2dpi_4.tif	224	33.284	0.149	100	56.14
MAX_dSarm^TIR_7dpe_2dpi_5.tif	95	9.756	0.103	100	48.64
MAX_dSarm^TIR_7dpe_2dpi_6.tif	67	8.491	0.127	100	65.68
MAX_dSarm^TIR_7dpe_2dpi_7.tif	290	30.169	0.104	100	55.37
MAX_dSarm^TIR_7dpe_2dpi_8.tif	107	4.063	0.038	100	56.67
MAX_dSarm^TIR_7dpe_2dpi_9.tif	158	31.848	0.202	100	82.9
MAX_dSarm^TIR_7dpe_2dpi_10.tif	235	28.077	0.119	100	59.03
MAX_dSarm^TIR_7dpe_2dpi_11.tif	63	6.691	0.106	100	50.18
		21.89264			
MAX_dSarmattPKO_7dpe_2dpi_1.tif	30	2.725	0.091	100	70
MAX_dSarmattPKO_7dpe_2dpi_2.tif	53	4.696	0.089	100	70.69
MAX_dSarmattPKO_7dpe_2dpi_3.tif	142	23.771	0.167	100	64.31
MAX_dSarmattPKO_7dpe_2dpi_4.tif	231	13.211	0.057	100	64.69
MAX_dSarmattPKO_7dpe_2dpi_5.tif	234	33.673	0.144	100	123.3
MAX_dSarmattPKO_7dpe_2dpi_6.tif	248	11.557	0.047	100	18.72
MAX_dSarmattPKO_7dpe_2dpi_7.tif	522	79.73	0.153	100	61.71
MAX_dSarmattPKO_7dpe_2dpi_8.tif	175	13.163	0.075	100	43.99
MAX_dSarmattPKO_7dpe_2dpi_9.tif	1067	66.373	0.062	100	44.43
MAX_dSarmattPKO_7dpe_2dpi_10.tif	212	17.931	0.085	100	55.09
MAX_dSarmattPKO_7dpe_2dpi_11.tif	780	47.906	0.061	100	46.22
MAX_dSarmattPKO_7dpe_2dpi_12.tif	244	13.966	0.057	100	50.64
		27.39183			
MAX_dSarmE642A_7dpe_2dpi_1.tif	399	20.51	0.051	100	21.96
MAX_dSarmE642A_7dpe_2dpi_2.tif	305	23.357	0.077	100	56.69
MAX_dSarmE642A_7dpe_2dpi_3.tif	187	15.863	0.085	100	54.38
MAX_dSarmE642A_7dpe_2dpi_4.tif	69	9.464	0.137	100	73.99
MAX_dSarmE642A_7dpe_2dpi_5.tif	349	26.106	0.075	100	46.1
MAX_dSarmE642A_7dpe_2dpi_6.tif	64	10.73	0.168	100	84.49
MAX_dSarmE642A_7dpe_2dpi_7.tif	260	51.118	0.197	100	84.07
MAX_dSarmE642A_7dpe_2dpi_8.tif	72	9.002	0.125	100	61.97
MAX_dSarmE642A_7dpe_2dpi_9.tif	233	148.39	0.637	100	107.3
MAX_dSarmE642A_7dpe_2dpi_10.tif	90	29.342	0.326	100	101.3
MAX_dSarmE642A_7dpe_2dpi_11.tif	34	11.63	0.342	100	127
MAX_dSarmE642A_7dpe_2dpi_12.tif	39	9.562	0.245	100	126
MAX_dSarmE642A_7dpe_2dpi_13.tif	128	11.897	0.093	100	57.31
		28.99777			
MAX_dSarmRescue_7dpe_2dpi_1.tif	187	29.342	0.157	100	78.24
MAX_dSarmRescue_7dpe_2dpi_2.tif	435	41.459	0.095	100	56.73

MAX_dSarmRescue_7dpe_2dpi_3.tif	202	46.909	0.232	100	87.09
MAX_dSarmRescue_7dpe_2dpi_4.tif	563	30.486	0.054	100	43.54
MAX_dSarmRescue_7dpe_2dpi_5.tif	395	38.247	0.097	100	69.79
MAX_dSarmRescue_7dpe_2dpi_6.tif	341	17.201	0.05	100	78.55
MAX_dSarmRescue_7dpe_2dpi_7.tif	426	28.053	0.066	100	40.83
MAX_dSarmRescue_7dpe_2dpi_8.tif	398	45.449	0.114	100	47.9
MAX_dSarmRescue_7dpe_2dpi_9.tif	291	37.469	0.129	100	83.45
MAX_dSarmRescue_7dpe_2dpi_10.tif	592	49.074	0.083	100	49.06
MAX_dSarmRescue_7dpe_2dpi_11.tif	355	25.717	0.072	100	101.4
MAX_dSarmRescue_7dpe_2dpi_12.tif	93	8.272	0.089	100	75.75
MAX_dSarmRescue_7dpe_2dpi_13.tif	87	25.425	0.292	100	92.38
MAX_dSarmRescue_7dpe_2dpi_14.tif	64	4.915	0.077	100	65.09
		30.57271			

Perim.	IntDen	dSARM rescue / +	dSARM KO / +	dSARM E1170A / +	dSARM ARM-SAM / +
0.834	21.88	29.342	2.725	20.51	16.131
0.865	14.05	41.459	4.696	23.357	38.174
1.443	120.3	46.909	23.771	15.863	34.135
1.175	10.92	30.486	13.211	9.464	33.284
1.008	6.889	38.247	33.673	26.106	9.756
1.026	11.8	17.201	11.557	10.73	8.491
0.944	7.085	28.053	79.73	51.118	30.169
0.567	2.389	45.449	13.163	9.002	4.063
1.166	30.58	37.469	66.373	148.39	31.848
0.997	9.633	49.074	17.931	29.342	28.077
1.001	7.116	25.717	47.906	11.63	6.691
		8.272	13.966	9.562	
		25.425		11.897	
0.891	8.555	4.915			
0.906	8.01				
1.191	15.12				
0.703	4.479	30.57271429			
1.081	21.73				
0.616	1.305				
1.123	12.68				
0.811	4.261				
0.708	3.696				
0.815	7.614				
0.726	3.705				
0.673	3.683				
0.649	1.44				
0.796	6.339				
0.858	5.782				
1.146	13.18				
0.792	4.568				
1.225	20.62				
1.367	22.85				
1.094	10.31				
2.354	110.1				
1.891	45.57				
1.538	65.33				
1.325	44.44				
0.916	6.891				
1.113	17.55				
0.939	6.965				

1.303	30.92
0.655	2.891
0.885	9.803
0.65	4.609
0.746	3.542
0.986	7.975
1.039	16.01
0.815	5.442
0.792	8.512
0.894	8.921
1.535	41.17
0.834	6.374

dSARM rescue / +	dSARM KO / +	dSARM E1170A / +	SARM ARM-SAM / +
0.95974468	0.08913177	0.67085964	0.52762734
1.35607848	0.15360102	0.76398189	1.24862973
1.53434201	0.77752338	0.51886136	1.11651846
0.99716367	0.43211734	0.30955707	1.08868319
1.25101748	1.10140695	0.85389867	0.31910807
0.56262587	0.37801681	0.35096655	0.27773131
0.91758291	2.60788098	1.67201379	0.98679495
1.48658701	0.43054731	0.29444556	0.13289628
1.22556995	2.17098814	4.85367438	1.0417132
1.60515679	0.58650337	0.95974468	0.91836792
0.8411749	1.56695279	0.38040456	0.21885528
0.27056806	0.45681256	0.31276255	
0.8316239		0.38913784	
0.16076427			

	Label	Area	**Mean**	
1	MAX_dSarmRescue_no inj_10dpe_1-0.50.tif	195.639	220.749	1.08903889
2	MAX_dSarmRescue_no inj_10dpe_1-0.50.tif	337.168	211.612	1.04396259
3	MAX_dSarmRescue_no inj_10dpe_2-0.50.tif	251.599	194.337	0.95873844
4	MAX_dSarmRescue_no inj_10dpe_2-0.50.tif	387.07	205.359	1.01311416
5	MAX_dSarmRescue_no inj_10dpe_3-0.50.tif	164.229	205.302	1.01283296
6	MAX_dSarmRescue_no inj_10dpe_3-0.50.tif	489.159	215.515	1.06321758
7	MAX_dSarmRescue_no inj_10dpe_4-0.50.tif	180.165	176.104	0.8687881
8	MAX_dSarmRescue_no inj_10dpe_4-0.50.tif	277.681	199.668	0.98503829
9	MAX_dSarmRescue_no inj_10dpe_5-0.50.tif	368.506	201.643	0.99478172
10	MAX_dSarmRescue_no inj_10dpe_5-0.50.tif	456.216	189.826	0.93648395
11	MAX_dSarmRescue_no inj_10dpe_6-0.50.tif	415.22	198.91	0.98129879
12	MAX_dSarmRescue_no inj_10dpe_6-0.50.tif	55.351	145.183	0.71624303
13	MAX_dSarmRescue_no inj_10dpe_7-0.50.tif	274.104	210.988	1.04088416
14	MAX_dSarmRescue_no inj_10dpe_7-0.50.tif	75.205	213.661	1.05407109
15	MAX_dSarmRescue_no inj_10dpe_8-0.50.tif	241.55	212.17	1.04671542
16	MAX_dSarmRescue_no inj_10dpe_8-0.50.tif	214.422	204.283	1.00780584
17	MAX_dSarmRescue_no inj_10dpe_9-0.50.tif	266.757	205.515	1.01388377
18	MAX_dSarmRescue_no inj_10dpe_9-0.50.tif	186.929	198.091	0.97725835
19	MAX_dSarmRescue_no inj_10dpe_10-0.50.tif	617.744	213.279	1.05218654
20	MAX_dSarmRescue_no inj_10dpe_10-0.50.tif	310.332	196.008	0.96698212
21	MAX_dSarmRescue_no inj_10dpe_11-0.50.tif	118.659	194.634	0.96020365
22	MAX_dSarmRescue_no inj_10dpe_11-0.50.tif	323.714	214.834	1.05985794
23	MAX_dSarmRescue_no inj_10dpe_12-0.50.tif	521.129	217.936	1.07516129
24	MAX_dSarmRescue_no inj_10dpe_12-0.50.tif	704.774	219.211	1.08145135
			202.70075	1
1	MAX_dSarm^ARM^SAM 11-3_no inj_10dpe_1-	100.995	86.909	0.4287552
2	MAX_dSarm^ARM^SAM 11-3_no inj_10dpe_1-	61.093	58.041	0.28633836
3	MAX_dSarm^ARM^SAM 11-3_no inj_10dpe_2-	39.634	57.084	0.28161711
4	MAX_dSarm^ARM^SAM 11-3_no inj_10dpe_2-	38.807	47.883	0.23622508
5	MAX_dSarm^ARM^SAM 11-3_no inj_10dpe_3-	96.031	82.492	0.40696445
6	MAX_dSarm^ARM^SAM 11-3_no inj_10dpe_3-	70.606	110.14	0.54336257
7	MAX_dSarm^ARM^SAM 11-3_no inj_10dpe_4-	49.585	60.882	0.30035409
8	MAX_dSarm^ARM^SAM 11-3_no inj_10dpe_4-	32.481	69.473	0.34273677
9	MAX_dSarm^ARM^SAM 11-3_no inj_10dpe_5-	19.902	51.615	0.25463645
10	MAX_dSarm^ARM^SAM 11-3_no inj_10dpe_5-	24.768	36.993	0.18250056
11	MAX_dSarm^ARM^SAM 11-3_no inj_10dpe_6-	70.509	70.213	0.34638747
12	MAX_dSarm^ARM^SAM 11-3_no inj_10dpe_6-	31.167	63.783	0.31466583
13	MAX_dSarm^ARM^SAM 11-3_no inj_10dpe_7-	21.8	30.171	0.14884503
14	MAX_dSarm^ARM^SAM 11-3_no inj_10dpe_7-	13.99	34.36	0.16951097
15	MAX_dSarm^ARM^SAM 11-3_no inj_10dpe_8-	56.324	68.384	0.33736432
16	MAX_dSarm^ARM^SAM 11-3_no inj_10dpe_8-	33.892	37.564	0.18531752
17	MAX_dSarm^ARM^SAM 11-3_no inj_10dpe_9-	56.13	52.394	0.25847956
18	MAX_dSarm^ARM^SAM 11-3_no inj_10dpe_9-	40.972	75.412	0.37203612
19	MAX_dSarm^ARM^SAM 11-3_no inj_10dpe_10	86.153	87.9	0.43364418

20	MAX_dSarm^ARM^SAM 11-3_no inj_10dpe_10	89.608	78.223	0.38590385
21	MAX_dSarm^ARM^SAM 11-3_no inj_10dpe_11	21.07	26.418	0.13033006
22	MAX_dSarm^ARM^SAM 11-3_no inj_10dpe_11	0.706	52.034	0.25670354
				0
1	MAX_dSarm^SAM_no inj_10dpe_2-0.50.tif	88.27	61.256	0.30219918
2	MAX_dSarm^SAM_no inj_10dpe_2-0.50.tif	121.311	82.158	0.4053167
3	MAX_dSarm^SAM_no inj_10dpe_3-0.50.tif	650.542	215.601	1.06364185
4	MAX_dSarm^SAM_no inj_10dpe_3-0.50.tif	260.09	190.115	0.9379097
5	MAX_dSarm^SAM_no inj_10dpe_4-0.50.tif	346.9	216.072	1.06596547
6	MAX_dSarm^SAM_no inj_10dpe_4-0.50.tif	280.211	202.759	1.00028737
7	MAX_dSarm^SAM_no inj_10dpe_5-0.50.tif	804.163	213.224	1.0519152
8	MAX_dSarm^SAM_no inj_10dpe_5-0.50.tif	245.516	183.841	0.90695767
9	MAX_dSarm^SAM_no inj_10dpe_6-0.50.tif	56.495	145.267	0.71665744
10	MAX_dSarm^SAM_no inj_10dpe_7-0.50.tif	45.011	57.617	0.2842466
11	MAX_dSarm^SAM_no inj_10dpe_7-0.50.tif	51.093	79.079	0.39012682
12	MAX_dSarm^SAM_no inj_10dpe_8-0.50.tif	523.343	192.143	0.9479146
13	MAX_dSarm^SAM_no inj_10dpe_8-0.50.tif	201.795	197.254	0.97312911
14	MAX_dSarm^SAM_no inj_10dpe_9-0.50.tif	267.535	200.306	0.98818579
15	MAX_dSarm^SAM_no inj_10dpe_9-0.50.tif	546.749	201.989	0.99648867
				0
1	MAX_dSarmattPKO_no inj_10dpe_1-0.50.tif	219.775	198.573	0.97963624
2	MAX_dSarmattPKO_no inj_10dpe_1-0.50.tif	459.33	213.146	1.0515304
3	MAX_dSarmattPKO_no inj_10dpe_2-0.50.tif	768.349	210.461	1.03828427
4	MAX_dSarmattPKO_no inj_10dpe_2-0.50.tif	572.49	200.849	0.99086461
5	MAX_dSarmattPKO_no inj_10dpe_3-0.50.tif	254.616	208.61	1.02915258
6	MAX_dSarmattPKO_no inj_10dpe_3-0.50.tif	207.245	165.306	0.81551746
7	MAX_dSarmattPKO_no inj_10dpe_4-0.50.tif	514.244	204.392	1.00834358
8	MAX_dSarmattPKO_no inj_10dpe_4-0.50.tif	270.601	191.964	0.94703152
9	MAX_dSarmattPKO_no inj_10dpe_5-0.50.tif	277.048	198.387	0.97871863
10	MAX_dSarmattPKO_no inj_10dpe_5-0.50.tif	38.198	53.58	0.26433055
11	MAX_dSarmattPKO_no inj_10dpe_6-0.50.tif	77.175	43.174	0.21299379
12	MAX_dSarmattPKO_no inj_10dpe_6-0.50.tif	42.432	77.426	0.38197195
13	MAX_dSarmattPKO_no inj_10dpe_7-0.50.tif	245.322	191.397	0.9442343
14	MAX_dSarmattPKO_no inj_10dpe_7-0.50.tif	115.106	181.544	0.89562569

Min	Max	Perim	IntDen	%Area	RawIntDen	Slice
86	255	434.685	43187.246	100	1775045	1
89	255	738.623	71348.704	100	2932513	1
87	255	842.75	48895.068	100	2009643	1
89	255	974.559	79488.211	100	3267056	1
88	255	502.232	33716.511	100	1385787	1
90	255	952.007	105421.069	100	4332926	1
81	255	724.917	31727.904	100	1304053	1
88	255	750.427	55443.987	100	2278811	1
91	255	1020.207	74306.53	100	3054083	1
89	255	1510.35	86601.59	100	3559424	1
89	255	1190.675	82591.531	100	3394606	1
73	255	243.608	8036.055	100	330291	1
95	255	607.237	57832.753	100	2376992	1
95	255	163.043	16068.29	100	660425	1
91	255	613.786	51249.675	100	2106420	1
90	255	555.386	43802.898	100	1800349	1
90	255	696.801	54822.52	100	2253268	1
85	255	473.634	37028.95	100	1521932	1
87	255	1439.727	131751.848	100	5415151	1
86	255	962.594	60827.609	100	2500084	1
86	255	421.501	23095.005	100	949231	1
97	255	694.761	69544.812	100	2858371	1
97	255	1085.756	113572.96	100	4667978	1
96	255	1365.66	154494.332	100	6349893	1
49	255	528.943	8777.397	100	360761	1
33	185	375.395	3545.911	100	145741	1
31	255	315.15	2262.468	100	92990	1
26	236	293.519	1858.172	100	76373	1
45	255	562.442	7921.776	100	325594	1
54	255	376.567	7776.573	100	319626	1
32	255	342.096	3018.821	100	124077	1
37	236	187.772	2256.531	100	92746	1
28	153	150.404	1027.246	100	42221	1
20	179	201.994	916.252	100	37659	1
40	232	368.172	4950.666	100	203478	1
39	169	165.902	1987.925	100	81706	1
17	124	201.72	657.719	100	27033	1
19	111	118.113	480.692	100	19757	1
38	216	287.204	3851.694	100	158309	1
21	186	249.691	1273.128	100	52327	1
29	219	388.779	2940.892	100	120874	1
41	203	186.874	3089.768	100	126993	1
49	255	476.386	7572.856	100	311253	1

43	255	422.79	7009.441	100	288096	1
13	255	238.618	556.627	100	22878	1
37	95	8.877	36.714	100	1509	1
33	255	579.557	5407.028	100	222235	1
44	255	573.675	9966.585	100	409638	1
88	255	1327.105	140257.67	100	5764750	1
81	255	745.352	49446.975	100	2032327	1
89	255	652.913	74955.295	100	3080748	1
89	255	727.508	56815.335	100	2335175	1
86	255	1613.222	171466.805	100	7047481	1
77	255	704.722	45136.024	100	1855142	1
67	255	343.927	8206.853	100	337311	1
31	252	343.965	2593.407	100	106592	1
43	255	268.438	4040.399	100	166065	1
84	255	1418.969	100556.605	100	4132991	1
87	255	501.366	39804.858	100	1636025	1
86	255	581.517	53588.807	100	2202561	1
91	255	1196.587	110437.475	100	4539106	1
85	255	669.651	43641.297	100	1793707	1
87	255	1054.052	97904.562	100	4023989	1
107	255	1892.272	161707.149	100	6646348	1
105	255	1549.904	114984.04	100	4725975	1
85	255	521.67	53115.511	100	2183108	1
81	255	802.264	34258.759	100	1408074	1
90	255	1072.969	105107.452	100	4320036	1
86	255	742.765	51945.689	100	2135027	1
89	255	731.994	54962.735	100	2259031	1
29	255	243.014	2046.659	100	84120	1
23	255	597.484	3331.951	100	136947	1
40	255	269.525	3285.335	100	135031	1
84	255	935.594	46953.735	100	1929852	1
80	255	431.72	20896.817	100	858883	1

	Label	Area	**Mean**	
1	MAX_dSarmRescue_no inj_7dpe_1-0.50.tif	228.096	**215.825**	**1.03733198**
2	MAX_dSarmRescue_no inj_7dpe_2-0.50.tif	223.692	**214.64**	**1.03163645**
3	MAX_dSarmRescue_no inj_7dpe_3-0.50.tif	215.079	**220.905**	**1.06174828**
4	MAX_dSarmRescue_no inj_7dpe_1-0.50-1.tif	166.565	**211.202**	**1.0151122**
5	MAX_dSarmRescue_no inj_7dpe_2-0.50.tif	449.696	**222.721**	**1.07047662**
6	MAX_dSarmRescue_no inj_7dpe_3-0.50.tif	266.708	**219.913**	**1.05698037**
13	MAX_dSarmRescue_no inj_7dpe_4-0.50.tif	394.563	**196.035**	**0.94221418**
14	MAX_dSarmRescue_no inj_7dpe_4-0.50.tif	187.61	**185.171**	**0.88999792**
15	MAX_dSarmRescue_no inj_7dpe_5-0.50.tif	411.448	**229.191**	**1.10157375**
16	MAX_dSarmRescue_no inj_7dpe_5-0.50.tif	370.598	**177.354**	**0.85242663**
17	MAX_dSarmRescue_no inj_7dpe_6-0.50.tif	441.399	**203.135**	**0.97633931**
18	MAX_dSarmRescue_no inj_7dpe_6-0.50.tif	240.091	**187.129**	**0.89940877**
19	MAX_dSarmRescue_no inj_7dpe_7-0.50.tif	212.695	**205.111**	**0.98583668**
20	MAX_dSarmRescue_no inj_7dpe_7-0.50.tif	468.819	**200.608**	**0.96419365**
21	MAX_dSarmRescue_no inj_7dpe_8-0.50.tif	195.664	**170.012**	**0.81713835**
22	MAX_dSarmRescue_no inj_7dpe_8-0.50.tif	357.168	**210.058**	**1.00961372**
23	MAX_dSarmRescue_no inj_7dpe_9-0.50.tif	124.473	**203.943**	**0.98022285**
24	MAX_dSarmRescue_no inj_7dpe_9-0.50.tif	336.755	**238.616**	**1.14687366**
25	MAX_dSarmRescue_no inj_7dpe_10-0.50.tif	270.649	**216.284**	**1.0395381**
26	MAX_dSarmRescue_no inj_7dpe_10-0.50.tif	409.551	**210.794**	**1.0131512**
27	MAX_dSarmRescue_no inj_7dpe_11-0.50.tif	162.064	**180.794**	**0.86896049**
28	MAX_dSarmRescue_no inj_7dpe_11-0.50.tif	122.673	**201.673**	**0.96931242**
29	MAX_dSarmRescue_no inj_7dpe_12-0.50.tif	437.044	**236.806**	**1.13817416**
30	MAX_dSarmRescue_no inj_7dpe_12-0.50.tif	363.761	**235.467**	**1.13173845**
			**208.057792**	**1.00000001**
7	MAX_dSarm^ARM^SAM 2-4_no inj_7dpe_1-0.50.tif	46.398	**118.941**	**0.5716729**
8	MAX_dSarm^ARM^SAM 2-4_no inj_7dpe_1-0.50.tif	43.648	**98.187**	**0.47192177**
9	MAX_dSarm^ARM^SAM 2-4_no inj_7dpe_2-0.50.tif	40.85	**78.64**	**0.37797191**
10	MAX_dSarm^ARM^SAM 2-4_no inj_7dpe_2-0.50.tif	67.2	**82.671**	**0.39734633**
11	MAX_dSarm^ARM^SAM 2-4_no inj_7dpe_3-0.50.tif	60.874	**89.774**	**0.43148589**
12	MAX_dSarm^ARM^SAM 2-4_no inj_7dpe_3-0.50.tif	60.315	**97.019**	**0.46630794**
1	MAX_dSarm^ARM^SAM 2-4_no inj_7dpe_4-0.50.tif	167.538	**128.242**	**0.61637682**
2	MAX_dSarm^ARM^SAM 2-4_no inj_7dpe_4-0.50.tif	52.359	**89.739**	**0.43131766**
3	MAX_dSarm^ARM^SAM 2-4_no inj_7dpe_5-0.50.tif	115.155	**98.707**	**0.47442107**
4	MAX_dSarm^ARM^SAM 2-4_no inj_7dpe_5-0.50.tif	31.021	**103.985**	**0.49978902**
5	MAX_dSarm^ARM^SAM 2-4_no inj_7dpe_6-0.50.tif	186.029	**233.934**	**1.1243703**
6	MAX_dSarm^ARM^SAM 2-4_no inj_7dpe_6-0.50.tif	348.822	**200.537**	**0.9638524**
7	MAX_dSarm^ARM^SAM 2-4_no inj_7dpe_7-0.50.tif	70.558	**105.588**	**0.50749362**
8	MAX_dSarm^ARM^SAM 2-4_no inj_7dpe_7-0.50.tif	43.892	**80.444**	**0.38664258**
9	MAX_dSarm^ARM^SAM 2-4_no inj_7dpe_8-0.50.tif	14.403	**77.568**	**0.37281949**
10	MAX_dSarm^ARM^SAM 2-4_no inj_7dpe_8-0.50.tif	26.325	**83.322**	**0.40047527**
11	MAX_dSarm^ARM^SAM 2-4_no inj_7dpe_9-0.50.tif	112.065	**73.719**	**0.35431983**
12	MAX_dSarm^ARM^SAM 2-4_no inj_7dpe_9-0.50.tif	35.668	**72.949**	**0.35061893**
13	MAX_dSarm^ARM^SAM 2-4_no inj_7dpe_10-0.50.tif	31.41	**73.919**	**0.3552811**

14	MAX_dSarm^ARM^SAM 2-4_no inj_7dpe_10-0.50.tif	37.177	**64.062**	**0.30790484**
15	MAX_dSarm^ARM^SAM 2-4_no inj_7dpe_11-0.50.tif	53.405	**106.295**	**0.51089171**
16	MAX_dSarm^ARM^SAM 2-4_no inj_7dpe_11-0.50.tif	87.881	**76.141**	**0.36596082**
17	MAX_dSarm^ARM^SAM 2-4_no inj_7dpe_12-0.50.tif	103.622	**94.217**	**0.45284053**
18	MAX_dSarm^ARM^SAM 2-4_no inj_7dpe_12-0.50.tif	52.432	**72.033**	**0.34621631**
				**0**
19	MAX_dSarmattPKO_no inj_7dpe_1-0.50.tif	37.25	**87.329**	**0.41973434**
20	MAX_dSarmattPKO_no inj_7dpe_1-0.50.tif	19.318	**70.759**	**0.34009301**
21	MAX_dSarmattPKO_no inj_7dpe_2-0.50.tif	231.672	**195.55**	**0.9398831**
22	MAX_dSarmattPKO_no inj_7dpe_2-0.50.tif	118.829	**171.11**	**0.82241573**
23	MAX_dSarmattPKO_no inj_7dpe_4-0.50.tif	38.344	**72.256**	**0.34728813**
24	MAX_dSarmattPKO_no inj_7dpe_4-0.50.tif	287.656	**174.727**	**0.83980033**
25	MAX_dSarmattPKO_no inj_7dpe_5-0.50.tif	32.238	**84.231**	**0.40484425**
26	MAX_dSarmattPKO_no inj_7dpe_5-0.50.tif	21.557	**67.052**	**0.32227584**
27	MAX_dSarmattPKO_no inj_7dpe_6-0.50.tif	16.423	**68.904**	**0.33117722**
28	MAX_dSarmattPKO_no inj_7dpe_6-0.50.tif	3.893	**86.513**	**0.41581236**
29	MAX_dSarmattPKO_no inj_7dpe_7-0.50.tif	67.662	**97.844**	**0.47027319**
30	MAX_dSarmattPKO_no inj_7dpe_7-0.50.tif	135.106	**95.011**	**0.45665678**
31	MAX_dSarmattPKO_no inj_7dpe_8-0.50.tif	296.075	**192.445**	**0.92495936**
32	MAX_dSarmattPKO_no inj_7dpe_8-0.50.tif	146.59	**153.795**	**0.73919366**
33	MAX_dSarmattPKO_no inj_7dpe_9-0.50.tif	376.9	**176.209**	**0.84692335**
34	MAX_dSarmattPKO_no inj_7dpe_9-0.50.tif	406.947	**171.062**	**0.82218503**
35	MAX_dSarmattPKO_no inj_7dpe_10-0.50.tif	274.494	**165.019**	**0.79314021**
36	MAX_dSarmattPKO_no inj_7dpe_10-0.50.tif	232.208	**185.967**	**0.89382378**
37	MAX_dSarmattPKO_no inj_7dpe_11-0.50.tif	173.62	**210.604**	**1.01223799**
38	MAX_dSarmattPKO_no inj_7dpe_11-0.50.tif	334.614	**144.52**	**0.6946147**
39	MAX_dSarmattPKO_no inj_7dpe_12-0.50.tif	297.924	**170.042**	**0.81728254**
40	MAX_dSarmattPKO_no inj_7dpe_12-0.50.tif	119.851	**181.477**	**0.87224324**
			**137.383**	**0.66031173**
1	MAX_dSarm^SAM_no inj_7dpe_9-0.50.tif	399.454	**145.044**	**0.69713323**
2	MAX_dSarm^SAM_no inj_7dpe_9-0.50.tif	506.288	**180.355**	**0.8668505**
3	MAX_dSarm^SAM_no inj_7dpe_8-0.50.tif	286.123	**156.953**	**0.75437214**
4	MAX_dSarm^SAM_no inj_7dpe_8-0.50.tif	423.443	**197.627**	**0.9498659**
5	MAX_dSarm^SAM_no inj_7dpe_5-0.50.tif	231.818	**216.25**	**1.03937469**
6	MAX_dSarm^SAM_no inj_7dpe_5-0.50.tif	338.944	**189.905**	**0.91275121**
7	MAX_dSarm^SAM_no inj_7dpe_4-0.50.tif	111.943	**155.678**	**0.74824403**
8	MAX_dSarm^SAM_no inj_7dpe_4-0.50.tif	334.249	**201.037**	**0.96625558**
9	MAX_dSarm^SAM_no inj_7dpe_3-0.50.tif	361.912	**200.836**	**0.9652895**
10	MAX_dSarm^SAM_no inj_7dpe_3-0.50.tif	306.95	**202.139**	**0.97155218**
11	MAX_dSarm^SAM_no inj_7dpe_2-0.50.tif	395.585	**194.794**	**0.93624949**
12	MAX_dSarm^SAM_no inj_7dpe_2-0.50.tif	600.324	**168.455**	**0.80965486**
13	MAX_dSarm^SAM_no inj_7dpe_1-0.50.tif	394.588	**186.206**	**0.8949725**
14	MAX_dSarm^SAM_no inj_7dpe_1-0.50.tif	129.169	**146.202**	**0.702699**
1	MAX_dSarm^SAM_no inj_7dpe_7-0.50.tif	50.388	**72.761**	**0.34971534**
2	MAX_dSarm^SAM_no inj_7dpe_7-0.50.tif	39.22	**44.663**	**0.21466632**

3	MAX_dSarm^SAM_no inj_7dpe_6-0.50.tif	107.88	**85.185**	**0.40942951**
4	MAX_dSarm^SAM_no inj_7dpe_6-0.50.tif	50.072	**64.984**	**0.3123363**
5	MAX_dSarm^SAM_no inj_7dpe_13-0.50.tif	112.649	**67.886**	**0.32628435**
6	MAX_dSarm^SAM_no inj_7dpe_13-0.50.tif	77.248	**66.777**	**0.3209541**
7	MAX_dSarm^SAM_no inj_7dpe_12-0.50.tif	49.439	**53.697**	**0.25808695**
8	MAX_dSarm^SAM_no inj_7dpe_12-0.50.tif	45.181	**50.757**	**0.24395626**
9	MAX_dSarm^SAM_no inj_7dpe_11-0.50.tif	34.525	**70.339**	**0.33807434**
10	MAX_dSarm^SAM_no inj_7dpe_11-0.50.tif	65.497	**107.185**	**0.51516937**
11	MAX_dSarm^SAM_no inj_7dpe_10-0.50.tif	30.218	**79.201**	**0.38066827**
12	MAX_dSarm^SAM_no inj_7dpe_10-0.50.tif	47.59	**52.138**	**0.25059384**

Min	Max	Perim	IntDen	%Area	RawIntDen	Slice
129	255	736.306	49228.709	100	2023356	1
129	255	642.737	48013.171	100	1973396	1
129	255	594.546	47512.138	100	1952803	1
129	255	653.866	35178.806	100	1445889	1
129	255	1157.97	100156.811	100	4116559	1
129	255	786.258	58652.511	100	2410685	1
83	255	1052.647	77348.124	100	3179096	1
78	255	665.878	34739.938	61.017	1427851	1
130	255	1002.724	94300.258	100	3875848	1
70	255	1075.78	65727.084	100	2701458	1
90	255	1232.091	89663.428	100	3685269	1
88	255	710.222	44928.001	100	1846592	1
80	255	376.737	43626.09	100	1793082	1
83	255	1099.832	94048.756	100	3865511	1
68	255	474.741	33265.137	100	1367235	1
87	255	767.084	75025.999	100	3083654	1
86	255	377.797	25385.452	100	1043371	1
129	255	595.56	80354.975	100	3302681	1
105	255	720.815	58537.04	100	2405939	1
95	255	1022.24	86330.819	100	3548295	1
76	255	488.395	29300.088	100	1204267	1
102	255	460.215	24739.874	100	1016837	1
129	255	791.398	103494.431	100	4253739	1
129	255	709.667	85653.685	100	3520464	1
80	255	281.074	5518.582	100	226820	1
62	255	268.138	4285.721	100	176148	1
45	255	229.95	3212.466	100	132036	1
47	255	372.993	5555.491	100	228337	1
60	255	391.183	5464.934	100	224615	1
57	255	328.637	5851.663	100	240510	1
77	255	767.882	21485.341	100	883072	1
55	255	302.037	4698.629	100	193119	1
55	255	620.007	11366.619	100	467181	1
60	255	138.961	3225.726	100	132581	1
129	255	396.339	43518.502	100	1788660	1
94	255	1070.584	69951.687	100	2875094	1
62	255	407.185	7450.061	100	306206	1
35	255	317.55	3530.827	100	145121	1
53	153	118.973	1117.244	100	45920	1
56	255	211.359	2193.467	100	90154	1
39	255	774.693	8261.304	100	339549	1
49	255	292.375	2601.947	100	106943	1
49	218	289.81	2321.809	100	95429	1

34	187	200.297	2381.589	100	97886	1
66	255	276.228	5676.655	100	233317	1
41	255	440.519	6691.347	100	275022	1
51	255	493.614	9763.038	100	401272	1
43	203	285.76	3776.805	100	155231	1
55	255	247.539	3252.976	100	133701	1
41	255	165.231	1366.945	100	56183	1
89	255	633.807	45303.538	100	1862027	1
73	255	351.908	20332.794	100	835701	1
45	255	225.133	2770.629	100	113876	1
69	255	735.121	50261.186	100	2065792	1
48	255	192.082	2715.399	100	111606	1
43	255	169.217	1445.41	100	59408	1
47	201	135.794	1131.599	100	46510	1
46	255	33.463	336.779	100	13842	1
53	255	345.265	6620.327	100	272103	1
45	255	533.133	12836.506	100	527595	1
81	255	888.36	56978.153	100	2341867	1
70	255	621.026	22544.752	100	926615	1
61	255	836.389	66413.269	100	2729661	1
57	255	1046.949	69613.059	100	2861176	1
31	255	644.063	45296.75	100	1861748	1
52	255	478.034	43182.891	100	1774866	1
97	255	421.764	36565.191	100	1502871	1
50	255	1167.996	48358.222	100	1987578	1
62	255	916.038	50659.545	100	2082165	1
74	255	393.995	21750.127	100	893955	1
62	255	1256.87	57938.467	100	2381337	1
56	255	924.262	91311.581	100	3753010	1
62	255	959.866	44907.855	100	1845764	1
76	255	893.047	83683.764	100	3439498	1
89	255	373.227	50130.703	100	2060429	1
62	255	558.309	64367.219	100	2645566	1
64	255	401.297	17427.108	100	716274	1
76	255	675.551	67196.483	100	2761852	1
49	255	607.389	72685.115	100	2987441	1
69	255	677.035	62046.504	100	2550182	1
78	255	896.425	77057.694	100	3167159	1
52	255	1347.161	101127.441	100	4156453	1
62	255	940.08	73474.558	100	3019888	1
60	255	492.422	18884.805	100	776187	1
42	255	434.044	3666.273	100	150688	1
24	255	365.202	1751.679	100	71996	1

47	247	521.405	9189.77	100	377710	1
36	181	266.648	3253.876	100	133738	1
38	230	542.629	7647.306	100	314313	1
37	207	375.395	5158.397	100	212016	1
30	215	359.257	2654.72	100	109112	1
28	229	331.148	2293.27	100	94256	1
38	255	233.245	2428.424	100	99811	1
58	255	228.551	7020.316	100	288543	1
40	255	202.698	2393.316	100	98368	1
28	180	320.919	2481.245	100	101982	1

	Label	Area	**Mean**
1	MAX_dSarmRescue_no inj_3dpe_1-0.50.tif	256.611	**136.047**
2	MAX_dSarmRescue_no inj_3dpe_1-0.50.tif	370.355	**222.127**
3	MAX_dSarmRescue_no inj_3dpe_2-0.50.tif	518.769	**209.979**
4	MAX_dSarmRescue_no inj_3dpe_2-0.50.tif	482.785	**198.165**
5	MAX_dSarmRescue_no inj_3dpe_3-0.50.tif	617.331	**220.186**
6	MAX_dSarmRescue_no inj_3dpe_3-0.50.tif	349.893	**187.756**
7	MAX_dSarmRescue_no inj_3dpe_4-0.50.tif	351.377	**222.928**
8	MAX_dSarmRescue_no inj_3dpe_4-0.50.tif	356.389	**226.765**
9	MAX_dSarmRescue_no inj_3dpe_5-0.50.tif	384.466	**235.66**
10	MAX_dSarmRescue_no inj_3dpe_5-0.50.tif	391.181	**226.819**
11	MAX_dSarmRescue_no inj_3dpe_6-0.50.tif	460.668	**221.132**
12	MAX_dSarmRescue_no inj_3dpe_6-0.50.tif	263.545	**200.682**
13	MAX_dSarmRescue_no inj_3dpe_7-0.50.tif	259.044	**196.559**
14	MAX_dSarmRescue_no inj_3dpe_7-0.50.tif	353.226	**186.614**
15	MAX_dSarmRescue_no inj_3dpe_8-0.50.tif	252.621	**206.846**
16	MAX_dSarmRescue_no inj_3dpe_8-0.50.tif	474.853	**173.851**
17	MAX_dSarmRescue_no inj_3dpe_9-0.50.tif	383.761	**241.585**
18	MAX_dSarmRescue_no inj_3dpe_9-0.50.tif	391.084	**242.193**
19	MAX_dSarmRescue_no inj_3dpe_10-0.50.tif	623.778	**217.292**
20	MAX_dSarmRescue_no inj_3dpe_10-0.50.tif	254.008	**218.493**
21	MAX_dSarmRescue_no inj_3dpe_12-0.50.tif	271.671	**237.318**
22	MAX_dSarmRescue_no inj_3dpe_12-0.50.tif	277.827	**230.457**
23	MAX_dSarmRescue_no inj_3dpe_13-0.50.tif	276.586	**220.843**
24	MAX_dSarmRescue_no inj_3dpe_13-0.50.tif	356.997	**186.762**
			**211.127458**
1	MAX_dSarm^ARM^SAM 2-4_no inj_3dpe_1-0.50.tif	91.92	**107.711**
2	MAX_dSarm^ARM^SAM 2-4_no inj_3dpe_1-0.50.tif	114.766	**113.611**
3	MAX_dSarm^ARM^SAM 2-4_no inj_3dpe_2-0.50.tif	269.044	**112.892**
4	MAX_dSarm^ARM^SAM 2-4_no inj_3dpe_2-0.50.tif	245.419	**96.016**
5	MAX_dSarm^ARM^SAM 2-4_no inj_3dpe_3-0.50.tif	137.125	**116.645**
6	MAX_dSarm^ARM^SAM 2-4_no inj_3dpe_3-0.50.tif	70.655	**77.092**
7	MAX_dSarm^ARM^SAM 2-4_no inj_3dpe_4-0.50.tif	398.967	**141.401**
8	MAX_dSarm^ARM^SAM 2-4_no inj_3dpe_4-0.50.tif	290.114	**130.014**
9	MAX_dSarm^ARM^SAM 2-4_no inj_3dpe_5-0.50.tif	333.811	**158.214**
10	MAX_dSarm^ARM^SAM 2-4_no inj_3dpe_5-0.50.tif	263.204	**119.747**
11	MAX_dSarm^ARM^SAM 2-4_no inj_3dpe_6-0.50.tif	284.931	**140.156**
12	MAX_dSarm^ARM^SAM 2-4_no inj_3dpe_6-0.50.tif	337.168	**129.967**
13	MAX_dSarm^ARM^SAM 2-4_no inj_3dpe_7-0.50.tif	165.348	**127.437**
14	MAX_dSarm^ARM^SAM 2-4_no inj_3dpe_7-0.50.tif	73.112	**62.076**
15	MAX_dSarm^ARM^SAM 2-4_no inj_3dpe_8-0.50.tif	335.344	**152.562**
16	MAX_dSarm^ARM^SAM 2-4_no inj_3dpe_8-0.50.tif	318.02	**125.556**
17	MAX_dSarm^ARM^SAM 2-4_no inj_3dpe_9-0.50.tif	168.389	**123.845**
18	MAX_dSarm^ARM^SAM 2-4_no inj_3dpe_9-0.50.tif	101.554	**106.671**
19	MAX_dSarm^ARM^SAM 2-4_no inj_3dpe_10-0.50.tif	262.401	**125.524**

20	MAX_dSarm^ARM^SAM 2-4_no inj_3dpe_10-0.50.tif	99.803	**109.144**
21	MAX_dSarm^ARM^SAM 2-4_no inj_3dpe_11-0.50.tif	324.565	**159.475**
22	MAX_dSarm^ARM^SAM 2-4_no inj_3dpe_11-0.50.tif	368.36	**133.882**
23	MAX_dSarm^ARM^SAM 2-4_no inj_3dpe_12-0.50.tif	278.192	**162.606**
24	MAX_dSarm^ARM^SAM 2-4_no inj_3dpe_12-0.50.tif	285.321	**130.579**
1	MAX_dSarmattPKO_no inj_3dpe_1-0.50.tif	224.203	**185.812**
2	MAX_dSarmattPKO_no inj_3dpe_1-0.50.tif	276.927	**125.356**
3	MAX_dSarmattPKO_no inj_3dpe_2-0.50.tif	623.632	**210.705**
4	MAX_dSarmattPKO_no inj_3dpe_2-0.50.tif	204.885	**219.533**
5	MAX_dSarmattPKO_no inj_3dpe_3-0.50.tif	762.923	**204.373**
6	MAX_dSarmattPKO_no inj_3dpe_3-0.50.tif	396.558	**161.438**
7	MAX_dSarmattPKO_no inj_3dpe_4-0.50.tif	366.121	**149.686**
8	MAX_dSarmattPKO_no inj_3dpe_4-0.50.tif	222.622	**147.851**
9	MAX_dSarmattPKO_no inj_3dpe_5-0.50.tif	630.615	**192.377**
10	MAX_dSarmattPKO_no inj_3dpe_5-0.50.tif	443.418	**176.992**
11	MAX_dSarmattPKO_no inj_3dpe_6-0.50.tif	228.047	**148.735**
12	MAX_dSarmattPKO_no inj_3dpe_6-0.50.tif	259.847	**128.748**
13	MAX_dSarmattPKO_no inj_3dpe_7-0.50.tif	301.841	**193.814**
14	MAX_dSarmattPKO_no inj_3dpe_7-0.50.tif	399.624	**201.844**
15	MAX_dSarmattPKO_no inj_3dpe_8-0.50.tif	500.789	**202.578**
16	MAX_dSarmattPKO_no inj_3dpe_8-0.50.tif	448.503	**205.691**
17	MAX_dSarmattPKO_no inj_3dpe_9-0.50.tif	281.89	**149.098**
18	MAX_dSarmattPKO_no inj_3dpe_9-0.50.tif	265.978	**145.137**
19	MAX_dSarmattPKO_no inj_3dpe_10-0.50.tif	281.525	**142.577**
20	MAX_dSarmattPKO_no inj_3dpe_10-0.50.tif	330.769	**188.567**
21	MAX_dSarmattPKO_no inj_3dpe_11-0.50.tif	216.198	**169.383**
22	MAX_dSarmattPKO_no inj_3dpe_11-0.50.tif	274.226	**209.913**
23	MAX_dSarmattPKO_no inj_3dpe_12-0.50.tif	295.977	**201.445**
24	MAX_dSarmattPKO_no inj_3dpe_12-0.50.tif	371.085	**190.397**
1	MAX_dSarm^SAM_no inj_3dpe_1-0.50.tif	193.085	**101.382**
2	MAX_dSarm^SAM_no inj_3dpe_1-0.50.tif	42.87	**76.636**
3	MAX_dSarm^SAM_no inj_3dpe_2-0.50.tif	155.786	**129.421**
4	MAX_dSarm^SAM_no inj_3dpe_2-0.50.tif	101.992	**120.497**
5	MAX_dSarm^SAM_no inj_3dpe_3-0.50.tif	248.898	**173.825**
6	MAX_dSarm^SAM_no inj_3dpe_3-0.50.tif	543.805	**199.505**
7	MAX_dSarm^SAM_no inj_3dpe_4-0.50.tif	375.513	**198.811**
8	MAX_dSarm^SAM_no inj_3dpe_4-0.50.tif	215.128	**178.683**
9	MAX_dSarm^SAM_no inj_3dpe_5-0.50.tif	182.866	**155.198**
10	MAX_dSarm^SAM_no inj_3dpe_5-0.50.tif	198.267	**88.579**
11	MAX_dSarm^SAM_no inj_3dpe_6-0.50.tif	192.647	**132.318**
12	MAX_dSarm^SAM_no inj_3dpe_6-0.50.tif	182.063	**138.465**
13	MAX_dSarm^SAM_no inj_3dpe_7-0.50.tif	256.757	**159.992**
14	MAX_dSarmattPKO_no inj_3dpe_7-0.50.tif	413.614	**197.675**

15	MAX_dSarmattPKO_no inj_3dpe_8-0.50.tif	462.055	**212.648**
16	MAX_dSarmattPKO_no inj_3dpe_8-0.50.tif	440.937	**206.178**
17	MAX_dSarmattPKO_no inj_3dpe_9-0.50.tif	220.432	**170.099**
18	MAX_dSarmattPKO_no inj_3dpe_9-0.50.tif	228.388	**155.025**

Norm Mean	Min	Max	Perim	IntDen	%Area	RawIntDen
**0.644383262**	64	255	965.501	34911.125	100	1434887
**1.05209906**	85	255	537.298	82265.895	100	3381222
**0.994560357**	85	255	1244.27	108930.558	100	4477170
**0.938603637**	77	255	1237.758	95670.901	100	3932183
**1.042905561**	98	255	1272.114	135927.62	100	5586780
**0.889301665**	73	255	933.663	65694.408	100	2700115
**1.055892976**	129	255	912.714	78331.771	100	3219525
**1.074066832**	129	255	860.944	80816.593	100	3321654
**1.11619778**	129	255	697.395	90603.207	100	3723895
**1.074322602**	93	255	555.132	88727.201	100	3646789
**1.047386267**	87	255	870.927	101868.418	100	4186908
**0.950525346**	85	255	646.608	52888.632	100	2173783
**0.930996858**	94	255	703.48	50917.47	100	2092766
**0.883892611**	82	255	1203.516	65917.103	100	2709268
**0.97972098**	89	255	690.143	52253.564	100	2147681
**0.823440976**	56	255	1254.568	82553.625	100	3393048
**1.144261397**	129	255	533.496	92710.886	100	3810523
**1.147141174**	148	255	802.945	94717.983	100	3893017
**1.029198201**	129	255	1904.976	135541.743	100	5570920
**1.034886708**	107	255	653.08	55498.949	100	2281070
**1.124050857**	129	255	596.724	64472.423	100	2649890
**1.091553899**	129	255	679.457	64027.155	100	2631589
**1.046017425**	91	255	525.68	61082.079	100	2510543
**0.884593609**	47	255	757.719	66673.529	100	2740358
**1.000000002**						
**0.510170496**	66	255	408.029	9900.723	100	406931
**0.538115701**	59	255	412.335	13038.665	100	535904
**0.534710175**	52	255	1006.547	30372.784	100	1248356
**0.454777417**	45	255	1016.678	23564.237	100	968517
**0.552486167**	68	255	645.901	15994.91	100	657409
**0.365144357**	51	255	488.717	5446.954	100	223876
**0.669742351**	65	255	1212.124	56414.3	100	2318692
**0.615808106**	60	255	990.61	37718.955	100	1550292
**0.749376711**	83	255	1282.665	52813.549	100	2170697
**0.567178713**	56	255	916.453	31518.056	100	1295428
**0.663845439**	73	255	1004.167	39934.903	100	1641370
**0.615585491**	60	255	1124.88	43820.902	100	1801089
**0.603602209**	59	255	497.799	21071.436	100	866060
**0.294021444**	32	255	438.988	4538.487	100	186537
**0.722606152**	63	255	984.372	51160.577	100	2102758
**0.594692899**	52	255	968.165	39929.526	100	1641149
**0.586588789**	65	255	916.713	20854.24	100	857133
**0.505244562**	51	255	557.021	10832.887	100	445244
**0.594541332**	59	255	1009.31	32937.676	100	1353776

**0.516957865**	53	255	544.209	10892.885	100	447710
**0.755349406**	89	255	1364.32	51759.879	100	2127390
**0.634128792**	56	255	980.433	49316.614	100	2026969
**0.77017931**	83	255	832.885	45235.778	100	1859242
**0.618484214**	65	255	1060.842	37256.802	100	1531297
**0**						
**0.880093957**	91	255	631.507	41659.721	100	1712262
**0.593745604**	60	255	1025.873	34714.512	100	1426806
**0.997999038**	82	255	1191.195	131402.466	100	5400791
**1.039812642**	144	255	744.746	44979.045	100	1848690
**0.968007676**	76	255	1606.306	155920.691	100	6408518
**0.764647107**	82	255	1555.53	64019.588	100	2631278
**0.708984049**	56	255	1011.195	54803.128	100	2252471
**0.700292617**	76	255	748.346	32914.83	100	1352837
**0.911188918**	79	255	1639.22	121316.151	100	4986232
**0.838318245**	77	255	1264.916	78481.548	100	3225681
**0.704479661**	72	255	736.514	33918.671	100	1394096
**0.609811728**	56	255	861.618	33454.815	100	1375031
**0.917995233**	83	255	692.912	58501.007	100	2404458
**0.95602913**	93	255	1038.334	80661.804	100	3315292
**0.959505703**	72	255	942.587	101448.794	100	4169661
**0.974250351**	87	255	1079.88	92253.234	100	3791713
**0.706199001**	56	255	767.45	42029.249	100	1727450
**0.687437823**	66	255	620.488	38603.212	100	1586636
**0.675312446**	65	255	1078.324	40138.863	42.347	1649753
**0.893142947**	80	255	906.091	62372.189	100	2563568
**0.802278404**	66	255	595.309	36620.25	100	1505134
**0.99424775**	66	255	546.077	57563.66	100	2365932
**0.954139276**	85	255	818.259	59623.263	100	2450584
**0.901810697**	72	255	963.49	70653.492	100	2903939
**0**						
**0.480193344**	48	255	926.573	19575.321	100	804568
**0.362984525**	49	255	314.191	3285.383	20.772	135033
**0.612999376**	65	255	634.159	20161.996	100	828681
**0.57073107**	64	255	423.573	12289.805	100	505125
**0.823317827**	89	255	934.724	43264.616	100	1778225
**0.944950514**	70	255	970.904	108491.811	100	4459137
**0.9416634**	87	255	912.043	74656.204	100	3068455
**0.846327625**	83	255	669.074	38439.762	100	1579918
**0.735091501**	80	255	611.091	28380.503	100	1166471
**0.41955225**	47	255	915.274	17562.287	100	721830
**0.626720945**	68	255	759.775	25490.705	100	1047697
**0.655836059**	72	255	721.065	25209.35	100	1036133
**0.757798164**	84	255	865.086	41079.008	100	1688394
**0.936282764**	86	255	1058.13	81761.092	100	3360474

**1.00720201**	90	255	869.818	98255.063	100	4038395
**0.976557014**	88	255	1057.918	90911.471	100	3736565
**0.80566972**	83	255	647.507	37495.263	100	1541098
**0.734272091**	81	255	594.594	35405.734	100	1455216

Slice
1
1
1
1
1
1
1
1
1
1
1
1
1
1
1
1
1
1
1
1
1
1
1
1
1
1
1
1
1
1
1
1
1
1
1
1
1
1
1
1
1
1
1

1
1
1
1
1

1
1
1
1
1
1
1
1
1
1
1
1
1
1
1
1
1
1
1
1
1
1
1
1

1
1
1
1
1
1
1
1
1
1
1
1
1
1

1
1
1
1

	Label	Area	Mean	Norm Mean
1	MAX_dSarm^ARM^SAM 2-4_no inj_1dpe_1-(	191.09	172.044	1.00348915
2	MAX_dSarm^ARM^SAM 2-4_no inj_1dpe_1-(	206.929	153.487	0.89525086
3	MAX_dSarm^ARM^SAM 2-4_no inj_1dpe_2-(	241.04	150.069	0.87531453
4	MAX_dSarm^ARM^SAM 2-4_no inj_1dpe_2-(	369.333	134.933	0.78703007
5	MAX_dSarm^ARM^SAM 2-4_no inj_1dpe_3-(	190.53	141.921	0.82778931
6	MAX_dSarm^ARM^SAM 2-4_no inj_1dpe_3-(	197.61	123.177	0.71846029
7	MAX_dSarm^ARM^SAM 2-4_no inj_1dpe_4-(	323.154	162.484	0.94772809
8	MAX_dSarm^ARM^SAM 2-4_no inj_1dpe_4-(	262.718	105.776	0.61696466
9	MAX_dSarm^ARM^SAM 2-4_no inj_1dpe_5-(	223.935	174.487	1.01773855
10	MAX_dSarm^ARM^SAM 2-4_no inj_1dpe_5-(	173.864	85.041	0.49602265
11	MAX_dSarm^ARM^SAM 2-4_no inj_1dpe_6-(	227.463	154.424	0.90071614
12	MAX_dSarm^ARM^SAM 2-4_no inj_1dpe_6-(	270.966	157.188	0.91683786
13	MAX_dSarm^ARM^SAM 11-3_no inj_1dpe_1	193.717	127.871	0.74583921
14	MAX_dSarm^ARM^SAM 11-3_no inj_1dpe_1	257.073	108.789	0.63453873
15	MAX_dSarm^ARM^SAM 11-3_no inj_1dpe_2	385.585	145.972	0.85141777
16	MAX_dSarm^ARM^SAM 11-3_no inj_1dpe_2	333.227	147.197	0.85856288
17	MAX_dSarm^ARM^SAM 11-3_no inj_1dpe_3	284.299	118.844	0.693187
18	MAX_dSarm^ARM^SAM 11-3_no inj_1dpe_4	280.625	190.702	1.11231655
19	MAX_dSarm^ARM^SAM 11-3_no inj_1dpe_5	136.785	181.704	1.05983349
20	MAX_dSarm^ARM^SAM 11-3_no inj_1dpe_5	229.848	134.329	0.78350709
				0
1	MAX_dSarm^SAM_no inj_1dpe_1-0.50.tif	125.593	115.645	0.67452804
2	MAX_dSarm^SAM_no inj_1dpe_1-0.50.tif	228.412	81.607	0.475993
3	MAX_dSarm^SAM_no inj_1dpe_2-0.50.tif	343.275	173.522	1.01210995
4	MAX_dSarm^SAM_no inj_1dpe_2-0.50.tif	219.605	123.761	0.72186662
5	MAX_dSarm^SAM_no inj_1dpe_3-0.50.tif	297.972	188.967	1.10219673
6	MAX_dSarm^SAM_no inj_1dpe_3-0.50.tif	149.582	115.224	0.67207246
7	MAX_dSarm^SAM_no inj_1dpe_4-0.50.tif	229.58	132.791	0.77453633
8	MAX_dSarm^SAM_no inj_1dpe_4-0.50.tif	210.894	101.959	0.59470107
9	MAX_dSarm^SAM_no inj_1dpe_5-0.50.tif	535.265	205.109	1.19634893
10	MAX_dSarm^SAM_no inj_1dpe_5-0.50.tif	312.643	159.39	0.92968157
				0
1	MAX_dSarmRescue_no inj_1dpe_1-0.50.tif	329.456	172.501	1.00615471
2	MAX_dSarmRescue_no inj_1dpe_1-0.50.tif	255.346	202.771	1.18271197
3	MAX_dSarmRescue_no inj_1dpe_2-0.50.tif	398.651	192.11	1.12052905
4	MAX_dSarmRescue_no inj_1dpe_2-0.50.tif	270.698	182.451	1.06419055
5	MAX_dSarmRescue_no inj_1dpe_3-0.50.tif	249.774	164.476	0.95934692
6	MAX_dSarmRescue_no inj_1dpe_3-0.50.tif	548.939	158.188	0.9226706
7	MAX_dSarmRescue_no inj_1dpe_4-0.50.tif	246.538	154.188	0.89933962
8	MAX_dSarmRescue_no inj_1dpe_4-0.50.tif	208.632	147.086	0.85791545
9	MAX_dSarmRescue_no inj_1dpe_5-0.50.tif	285.296	212.247	1.23798308
10	MAX_dSarmRescue_no inj_1dpe_5-0.50.tif	269.92	166.682	0.97221396
11	MAX_dSarmRescue_no inj_1dpe_6-0.50.tif	196.88	147.979	0.86312409
12	MAX_dSarmRescue_no inj_1dpe_6-0.50.tif	171.552	170.182	0.99262857

13	MAX_dSarmRescue_no inj_1dpe_7-0.50.tif	356.657	**182.194**	**1.06269153**
14	MAX_dSarmRescue_no inj_1dpe_7-0.50.tif	220.918	**162.525**	**0.94796723**
15	MAX_dSarmRescue_no inj_1dpe_8-0.50-02.t	282.863	**165.621**	**0.96602541**
16	MAX_dSarmRescue_no inj_1dpe_8-0.50-02.t	167.319	**161.931**	**0.94450258**
			**171.44575**	**0.99999971**
1	MAX_dSarmattPKO_no inj_1dpe_1-0.50.tif	362.107	**206.282**	**1.20319075**
2	MAX_dSarmattPKO_no inj_1dpe_1-0.50.tif	480.473	**207.778**	**1.21191654**
3	MAX_dSarmattPKO_no inj_1dpe_2-0.50.tif	252.742	**199.435**	**1.16325393**
4	MAX_dSarmattPKO_no inj_1dpe_2-0.50.tif	245.103	**113.046**	**0.65936873**
5	MAX_dSarmattPKO_no inj_1dpe_3-0.50.tif	208.096	**171.315**	**0.99923708**
6	MAX_dSarmattPKO_no inj_1dpe_3-0.50.tif	281.671	**153.704**	**0.89651657**
7	MAX_dSarmattPKO_no inj_1dpe_4-0.50.tif	279.846	**125.892**	**0.7342962**
8	MAX_dSarmattPKO_no inj_1dpe_4-0.50.tif	252.572	**204.194**	**1.19101197**
9	MAX_dSarmattPKO_no inj_1dpe_6-0.50.tif	350.55	**181.952**	**1.06128001**
10	MAX_dSarmattPKO_no inj_1dpe_6-0.50.tif	373.323	**201.761**	**1.1768209**
11	MAX_dSarmattPKO_no inj_1dpe_7-0.50.tif	190.944	**188.636**	**1.10026609**
12	MAX_dSarmattPKO_no inj_1dpe_7-0.50.tif	161.674	**145.387**	**0.84800561**
13	MAX_dSarmattPKO_no inj_1dpe_8-0.50.tif	271.258	**190.94**	**1.11370474**
14	MAX_dSarmattPKO_no inj_1dpe_8-0.50.tif	225.857	**121.536**	**0.70888876**
15	MAX_dSarmattPKO_no inj_1dpe_9-0.50.tif	241.94	**148.89**	**0.86843772**
16	MAX_dSarmattPKO_no inj_1dpe_9-0.50.tif	244.397	**161.237**	**0.94045465**
17	MAX_dSarmattPKO_no inj_1dpe_10-0.50.tif	305.32	**172.969**	**1.00888444**
18	MAX_dSarmattPKO_no inj_1dpe_10-0.50.tif	252.791	**167.479**	**0.97686266**

Min	Max	Perim	IntDen	%Area	RawIntDen	Slice
95	255	560.551	32875.853	100	1351235	1
80	255	720.48	31760.92	100	1305410	1
78	255	843.426	36172.501	100	1486731	1
77	255	1233.795	49835.261	100	2048286	1
76	255	678.7	27040.151	100	1111381	1
69	255	780.189	24341.053	100	1000445	1
81	255	1022.187	52507.304	100	2158110	1
58	255	1134.695	27789.327	100	1142173	1
81	255	618.936	39073.71	100	1605974	1
46	255	952.862	14785.624	100	607706	1
83	255	887.216	35125.766	100	1443709	1
78	255	884.535	42592.494	100	1750600	1
72	255	716.012	24770.725	100	1018105	1
56	255	1094.552	27966.816	100	1149468	1
78	255	1523.373	56284.572	100	2313360	1
80	255	1213.534	49050.076	100	2016014	1
68	255	1459.271	33787.166	100	1388691	1
125	255	878.773	53515.743	100	2199558	1
120	255	547.771	24854.348	100	1021542	1
81	255	895.793	30875.251	100	1269008	1
62	255	587.356	14524.172	100	596960	1
45	255	1209.329	18639.994	100	766125	1
86	255	882.478	59565.916	100	2448227	1
66	255	811.379	27178.395	100	1117063	1
89	255	747.999	56306.858	100	2314276	1
63	255	528.288	17235.508	100	708399	1
70	255	865.579	30486.211	100	1253018	1
55	255	893.527	21502.543	100	883779	1
85	255	1208.417	109787.712	100	4512400	1
79	255	966.445	49832.147	100	2048158	1
88	255	1042.541	56831.369	100	2335834	1
99	255	509.901	51776.813	100	2128086	1
85	255	973.535	76584.739	100	3147720	1
81	255	695.076	49389.069	100	2029947	1
82	255	704.695	41081.757	100	1688507	1
81	255	1876.635	86835.574	100	3569041	1
78	255	955.645	38013.204	100	1562386	1
76	255	784.907	30686.79	100	1261262	1
94	255	525.627	60553.188	100	2488805	1
83	255	780.07	44990.724	100	1849170	1
75	255	739.282	29134.132	100	1197446	1
79	255	597.158	29195.103	100	1199952	1

86	255	917.41	64980.851	100	2670787	1
81	255	653.259	35904.869	100	1475731	1
78	255	909.057	46847.996	100	1925506	1
73	255	589.156	27094.164	100	1113601	1
86	255	830.975	74696.178	100	3070098	1
100	255	1045.912	99831.589	100	4103192	1
100	255	553.355	50405.708	100	2071732	1
65	255	1129.09	27707.991	100	1138830	1
86	255	545.505	35649.985	100	1465255	1
74	255	1010.142	43293.885	100	1779428	1
75	255	1516.869	35230.362	100	1448008	1
92	255	571.65	51573.656	100	2119736	1
85	255	947.003	63783.123	100	2621559	1
86	255	739.93	75322	100	3095820	1
84	255	506.419	36018.856	100	1480416	1
73	255	574.7	23505.31	100	966095	1
96	255	643.107	51793.893	100	2128788	1
69	255	951.737	27449.896	100	1128222	1
79	255	931.558	36022.408	100	1480562	1
79	255	741.462	39405.818	100	1619624	1
88	255	1094.197	52810.848	100	2170586	1
87	255	812.628	42337.318	100	1740112	1

Date	Genotype	Lobe	ctions analyz	rpr expressio	Avg/day	Normalized
7/2/18	Split>OR2_4	L	3--6	23915.239	46129.7271	0.51843443
	Split>OR2_3	R	6--9	42231.67		0.91549794
	Split>OR2_3	L	5--8	53187.844		1.15300582
	Split>OR2_2	R	5--8	42588.393		0.92323098
	Split>OR2_2	R	4--7	48661.637		1.05488673
	Split>OR2_1	R	4--7	54420.85		1.17973492
	Split>OR2_1	L	3--7	57902.457		1.25520918

Genotype	Lobe	ctions analyz	rpr expressio	Normalized
Split>Ask1 R	L	4--7	29117.257	0.63120376
Split>Ask1 R	R	4--7	32445.448	0.70335226
Split>Ask1 R	R	2--5	47668.583	1.03335931
Split>Ask1 R	L	5--8	29539.762	0.64036282
Split>Ask1 R	R	3--6	35659.953	0.77303629
Split>Ask1 R	L	5--8	47625.901	1.03243405
Split>Ask1 R	R	5--8	47764.904	1.03544736
Split>Ask1 R	L	6--9	41499.252	0.89962058

Date	Genotype	Lobe	Sections analyzed	Drpr expression	Avg/day
2/16/19	OR_drpr-568, 1-300_3	L	4--8	29822.051	32180.9423
		R	6--10	35351.312	32180.9423
	OR_drpr-568, 1-300_1	L	6--10	33603.624	32180.9423
		R	9--13	29946.782	32180.9423
	OR_drpr-568, 1-300_2				
	OR_drpr-568, 1-300_4				
	OR_drpr-568, 1-300_5				
	OR_drpr-568, 1-300_6				
	OR_drpr-568, 1-300_7				

Normalized
0.92669912
1.098517
1.04420883
0.93057505
1

Genotype	Lobe	Sections analyzed	Drpr expression	Normalized
Ask1 KAH146	L	8--12	31551.11	0.98042841
	R	8--12	29476.3	0.91595516
Ask1 KAH146	L	4--8	26031.11	0.80889832
	R	10--14	21731.065	0.67527746
Ask1 KAH146_drpr-568, 1-300_1				
				0.84513984

Date	Genotype	Lobe	ctions analyz	rpr expressio	Avg/day	Normalized
3/15/19	SplitCherry-C	L	X		36633.6912	0.91615576
		R	8--12	33562.167		0.93477807
	SplitCherry-C	L	4--8	34244.371		0.84743101
		R	5--9	31044.526		1.10561016
	SplitCherry-C	L	4--8	40502.581		1.10561016
		R	5--9	37359.077		1.01980106
	SplitCherry-C	L	3--7	37018.208		1.01049626
		R	3--7	37087.244		1.01238076
	SplitCherry-C	L	X			
		R	X			
	SplitCherry-C	L	X			
		R	X			
	SplitCherry-C	L	2--6	39486.283		1.07786799
		R	3--7	39371.985		1.07474796
	SplitCherry-C	L	7--11	33865.119		0.92442552
		R	5--9	40389.068		1.10251156
	SplitCherry-C	L	4--8	38186.119		1.04237705
		R	3--7	34121.237		0.93141684

Genotype	Lobe	ctions analyz	rpr expressio	Normalized
SplitCherry-A	L	9--13	36162.39	0.98713476
	R	8--12	33321.706	0.90959183
SplitCherry-A	L	3--7	34557.389	0.94332261
	R	3--7	35742.641	0.97567676
SplitCherry-A	L	4--8	33700.264	0.91992543
	R	4--8	31592.425	0.86245721
SplitCherry-A	L	10--14	29953.403	0.81764633
	R	6--10	34454.828	0.94052297
SplitCherry-A	L	2--6	27126.177	0.7405309
	R	2--6	29121.366	0.79493398
SplitCherry-A	L	X		
	R	3--7	34423.999	0.93968142
SplitCherry-A	L	4--8	28945.983	0.7901465
	R	5--9	21864.082	0.59687837
				0.86295762

Date	Genotype	Lobe	Sections analyzed	Drpr expression	Avg/day	Normalized
3/20/19	MAX_OR_drpr-488_3L,R-7--11.tif:1			32551.044	27458.3367	1.18547035
	MAX_OR_drpr-488_3L,R-7--11.tif:1			31709.155	27458.3367	1.15480975
	MAX_OR_drpr-488_4L,R-6--10.tif:1			30673.84	27458.3367	1.11710481
	MAX_OR_drpr-488_4L,R-6--10.tif:1			30496.364	27458.3367	1.11064134
	MAX_OR_drpr-488_5L,R-5--9.tif:1			26338.503	27458.3367	0.95921699
	MAX_OR_drpr-488_5L,R-5--9.tif:1			25377.768	27458.3367	0.92422816
	MAX_OR_drpr-488_6L,R-7--11.tif:1			26348.099	27458.3367	0.95956646
	MAX_OR_drpr-488_6L,R-7--11.tif:1			26466.607	27458.3367	0.96388238
	MAX_OR_drpr-488_7L,R-6--10.tif:1			30887.653	27458.3367	1.12489162
	MAX_OR_drpr-488_8L,R-8--12.tif:1			28053.829	27458.3367	1.02168712
	MAX_OR_drpr-488_9R-8--12.tif:1			24641.981	27458.3367	0.89743167
	MAX_OR_drpr-488_9L-10--12.tif:1			22549.977	27458.3367	0.82124337
	MAX_OR_drpr-488_10L-6--10.tif:1			24834.605	27458.3367	0.90444681
	MAX_OR_drpr-488_10R-8--12.tif:1			23487.289	27458.3367	0.85537916

Genotype	Lobe	Sections analyzed	Drpr expression	Normalized
MAX_Ask1dsRed+3_drpr-488_1L-10--14			15805.105	0.57560315
MAX_Ask1dsRed+3_drpr-488_1R-8--12.			16230.249	0.59108639
MAX_Ask1dsRed+3_drpr-488_2L-8--12.			16554.431	0.60289271
MAX_Ask1dsRed+3_drpr-488_2R-6--10.			17155.383	0.62477867
MAX_Ask1dsRed+3_drpr-488_4L-8--12.			18863.5	0.68698626
MAX_Ask1dsRed+3_drpr-488_4R-10--14			17092.687	0.62249535
MAX_Ask1dsRed+3_drpr-488_5L-6--10.			17665.3	0.64334924
MAX_Ask1dsRed+3_drpr-488_5R-7--11.			18441.405	0.67161406
MAX_Ask1dsRed+3_drpr-488_3.czi - As			17070.999	0.6217055
MAX_Ask1dsRed+3_drpr-488_3L-7--11.			19818.919	0.72178148
				0.63622928

Date	Genotype	Lobe	Sections analyzed	Drpr expression	Avg/day	Normalized
4/15/19	SplitCherry-L	L	1--4	35878.951	31972.8431	1.12216955
		R	1--4	35248.607		1.10245457
	SplitCherry-L	L	2--5	28328.218		0.88600873
		R	2--5	31288.502		0.97859618
	SplitCherry-L	L	8--12	32440.413		1.01462397
		R	8--12	29316.383		0.91691511
	SplitCherry-L	L	1--4	31636.879		0.9894922
		R	1--4	30891.381		0.9661756
	SplitCherry-L	L	2--5	27636.571		0.8643764
		R	2--5	35103.029		1.09790139
	SplitCherry-L	L	1--4	28164.998		0.88090377
		R	1--4	25023.746		0.78265627
	SplitCherry-L	L	1--4	30072.874		0.94057553
		R	1--4	40570.333		1.26889976
	SplitCherry-L	L	1--4	30717.167		0.96072679
		R	1--4	35849.247		1.12124051
	SplitCherry-L	L	1--4	34644.418		1.08355763
		R	1--4	32699.459		1.02272603

Date	Genotype	Lobe	Sections analyzed	Drpr expression	Normalized
	SplitCherry-d	L		22647.291	0.70832897
		R		19519.147	0.61049144
	SplitCherry-d	L	3--7	19793.164	0.61906174
		R	3--7	24182.873	0.75635667
	SplitCherry-d	L		28752.577	0.89928121
		R		21497.562	0.67236942
	SplitCherry-d	L	1--4	19499.52	0.60987757
		R	1--4	23453.648	0.73354903
	SplitCherry-d	L	1--4	23123.499	0.72322311
		R	1--4	28405.293	0.88841937
	SplitCherry-d	L	3--7	28534.532	0.89246152
		R	3--7	32825.283	1.02666137
	SplitCherry-d	L	5--9	24926.955	0.77962898
		R	5--9	26482.535	0.82828214
	SplitCherry-d	L	2--5	26214.235	0.81989065
		R	2--5	25668.731	0.80282917
	SplitCherry-d	L	1--4	23074.273	0.72168349
		R	1--4	27921.747	0.87329572
	SplitCherry-d	L	3--7	27471.505	0.85921371
		R	3--7	31222.014	0.97651666
	SplitCherry-d	L	2--5	23455.944	0.73362084
		R	2--5	22024.424	0.68884784

Date		Dcp-1 Count		
9/28/20		**Left Brain Lo**	**Right Brain L**	**Total**
	Split_OR_1	615	547	1162
	Split_OR_2	219	193	412
	Split_OR_3	341	437	778
	Split_OR_4	563	347	910
	Split_OR_5	355	295	650
	Split_OR_6	558	507	1065
	Split_OR_7	276	245	521
	Split_OR_8	372	342	714
	Split_OR_9	237	231	468
	Split_dSARM RNAi #2_1	635	668	1303
	Split_dSARM RNAi #2_2	693	640	1333
	Split_dSARM RNAi #2_3	383	463	846
	Split_dSARM RNAi #2_4	511	530	1041
	Split_dSARM RNAi #2_5	748	762	1510
	Split_dSARM RNAi #2_6	606	612	1218
	Split_dSARM RNAi #2_7	626	716	1342
	Split_dSARM RNAi #2_8	818	783	1601
	Split_dSARM RNAi #2_9	686	704	1390

Dcp-1 Volume

Left Brain Lo	Right Brain L	Total
9286	9200	18486
3617	3402	7019
8148	9059	17207
8629	8602	17231
6433	5632	12065
1.10E+04	1.07E+04	21700
9426	5763	15189
7780	6280	14060
8381	7540	15921
9955	1.04E+04	20355
1.17E+04	1.10E+04	22700
6431	7518	13949
8180	7925	16105
1.39E+04	1.44E+04	28300
1.11E+04	1.07E+04	2.18E+04
1.22E+04	1.03E+04	2.25E+04
1.32E+04	1.32E+04	26400
1.20E+04	1.21E+04	24100

Brain Volume

Left Brain Lo	Right Brain L	Total
1.23E+06	1.24E+06	2.47E+06
5.95E+05	6.64E+05	1.26E+06
1.32E+06	1.26E+06	2.58E+06
1.41E+06	1.42E+06	2.83E+06
9.29E+05	9.09E+05	1.84E+06
1.47E+06	1.51E+06	2.98E+06
1.07E+06	6.52E+05	1.72E+06
1.40E+06	1.28E+06	2.68E+06
1.09E+06	1.06E+06	2.15E+06
1.23E+06	1.21E+06	2.44E+06
9.77E+05	1.04E+06	2.02E+06
7.26E+05	7.90E+05	1.52E+06
9.78E+05	9.04E+05	1.88E+06
1.41E+06	1.42E+06	2.83E+06
1.04E+06	1.03E+06	2.07E+06
1.18E+06	1.17E+06	2.35E+06
1.06E+06	1.01E+06	2.07E+06
1.18E+06	1.11E+06	2.29E+06

Normalized Dcp-1 Count			Normalized Dcp-1 Volume		
**Left Brain Lo**	**Right Brain L**	**Total**		**Left Brain Lo**	**Right Brain L**
0.0005	0.00044113	0.00047045		0.00754959	7.42E-03
0.00036807	0.00029066	0.00032724		0.00607899	5.12E-03
2.58E-04	3.47E-04	0.00030155		0.00617273	0.00718968
3.99E-04	2.44E-04	0.00032155		0.00611986	6.06E-03
3.82E-04	3.25E-04	0.00035365		0.00692465	6.20E-03
3.80E-04	3.36E-04	0.00035738		0.00748299	7.09E-03
2.58E-04	3.76E-04	0.00030256		0.00880935	8.84E-03
2.66E-04	2.67E-04	0.00026642		0.00555714	4.91E-03
2.17E-04	2.18E-04	0.00021767		0.00768899	7.11E-03
5.16E-04	5.52E-04	0.00053402		0.0080935	0.00859504
7.09E-04	6.15E-04	0.00066088		0.01197544	0.01057692
5.28E-04	5.86E-04	0.00055805		0.00885813	0.00951646
5.22E-04	5.86E-04	0.00055313		0.00836401	0.00876659
5.30E-04	5.37E-04	0.00053357		0.00985816	0.01014085
5.83E-04	5.94E-04	0.00058841		0.01067308	0.01038835
5.31E-04	6.12E-04	0.00057106		0.01033898	0.00880342
7.72E-04	7.75E-04	0.00077343		0.01245283	0.01306931
5.81E-04	6.34E-04	0.00060699		0.01016949	0.0109009

Total
0.00748421
0.00557506
0.00666938
0.00608869
0.0065642
0.00728188
0.00882056
0.00524627
0.00740512
8.34E-03
0.01125434
0.00920119
0.00855739
0.01
0.0105314
0.00957447
0.01275362
0.01052402

Date		Dcp-1 Count			Dcp-1 Volum
10/8/20		**Left Brain Lo**	**Right Brain L**	**Total**	**Left Brain Lo**
	Split_Ask1 RNAi #2_1	654	909	1563	1.30E+04
	Split_Ask1 RNAi #2_2	675	636	1311	1.21E+04
	Split_Ask1 RNAi #2_3	367	451	818	6.37E+03
	Split_Ask1 RNAi #2_4	620	443	1063	9.99E+03
	Split_Ask1 RNAi #2_5	620	787	1407	1.35E+04
	Split_Ask1 RNAi #2_6	749	782	1531	1.40E+04
	Split_OR_1	369	532	901	9643
	Split_OR_2	361	362	723	7669
	Split_OR_3	381	380	761	6498
	Split_OR_4	187	195	382	3362
	Split_OR_5	301	317	618	5147
	Split_OR_6	307	338	645	5894
	Split_OR_7	439	387	826	7767
	Split_OR_8	356	367	723	6078
	Split_OR_9	459	487	946	8439

e			Brain Volume		
Right Brain L	Total		Left Brain Lo	Right Brain L	Total
1.43E+04	2.73E+04		1.28E+06	1.38E+06	2.66E+06
1.24E+04	2.45E+04		1.22E+06	1.16E+06	2.38E+06
7.07E+03	1.34E+04		6.95E+05	7.13E+05	1.41E+06
9.49E+03	1.95E+04		9.44E+05	8.97E+05	1.84E+06
1.57E+04	2.92E+04		1.21E+06	1.23E+06	2.44E+06
1.41E+04	2.81E+04		1.30E+06	1.28E+06	2.58E+06
8990	18633		1.35E+06	1.30E+06	2.65E+06
7903	15572		8.98E+05	8.94E+05	1.79E+06
7628	14126		1.14E+06	1.10E+06	2.24E+06
3924	7286		5.44E+05	5.64E+05	1.11E+06
6055	11202		7.78E+05	7.88E+05	1.57E+06
6939	12833		9.89E+05	9.27E+05	1.92E+06
8209	15976		1.03E+06	1.05E+06	2.08E+06
6749	12827		9.65E+05	8.93E+05	1.86E+06
7729	16168		1.19E+06	1.20E+06	2.39E+06

Normalized Dcp-1 Count

Left Brain Lo	Right Brain L	Total
0.00051094	0.0006587	0.00058759
0.00055328	0.00054828	0.00055084
0.00052806	0.00063254	0.00058097
0.00065678	0.00049387	0.0005774
0.0005124	0.00063984	0.00057664
0.00057615	0.00061094	0.00059341
0.00027333	0.00040923	0.00034
0.000402	0.00040492	0.00040346
0.00033421	0.00034545	0.00033973
0.00034375	0.00034574	0.00034477
0.00038689	0.00040228	0.00039464
0.00031041	0.00036462	0.00033664
0.00042621	0.00036857	0.00039712
0.00036891	0.00041097	0.00038913
0.00038571	0.00040583	0.00039582

Normalized Dcp-1 Volume

Left Brain Lo	Right Brain L	Total
0.01015625	0.01036232	0.01026316
0.00991803	0.01068966	0.01029412
0.00916403	0.00992006	0.00954688
0.01058686	0.01057971	0.01058338
0.01115702	0.01276423	0.01196721
0.01076923	0.01101563	0.01089147
0.00714296	0.00691538	0.00703132
0.00854009	0.00884004	0.00868973
0.0057	0.00693455	0.00630625
0.00618015	0.00695745	0.00657581
0.00661568	0.00768401	0.00715326
0.00595956	0.00748544	0.00669781
0.00754078	0.0078181	0.00768077
0.00629845	0.00755767	0.00690366
0.0070916	0.00644083	0.00676485

Date		Dcp-1 Count		
4/5/21		Left Brain Lo	Right Brain L	Total
	Split_Ask1 ^N_1	199	287	486
	Split_Ask1 ^N_2	276	167	443
	Split_Ask1 ^N_3	659	343	1002
	Split_Ask1 ^N_4	264	485	749
	Split_Ask1 ^N_5	541	183	724
	Split_Ask1 ^N_6	84	192	276
	Split_Ask1 ^N_7	220	223	443
	Split_Ask1 ^N_8	233	214	447
	Split_Ask1 ^N_9	298	360	658
	Split_Ask1 ^N_10	259	160	419
	Split_Ask1 ^N_11	404	363	767
	Split_Ask1 ^N_12	163	326	489
	Split_Ask1 ^N_13	337	303	640
	Split_Ask1 FLAG_1	137	203	340
	Split_Ask1 FLAG_2	222	143	365
	Split_Ask1 FLAG_3	288	177	465
	Split_Ask1 FLAG_4	172	233	405
	Split_Ask1 FLAG_5	275	233	508
	Split_Ask1 FLAG_6	260	254	514
	Split_Ask1 FLAG_7	643	465	1108
	Split_Ask1 FLAG_8	269	328	597
	Split_Ask1 FLAG_9	232	275	507
	Split_Ask1 FLAG_10	550	309	859
	Split_Ask1 FLAG_11	384	312	696
	Split_dSARM RNAi #2_1	395	654	1049
	Split_dSARM RNAi #2_2	413	733	1146
	Split_dSARM RNAi #2_3	566	449	1015
	Split_dSARM RNAi #2_4	370	556	926
	Split_dSARM RNAi #2_5	576	532	1108
	Split_dSARM RNAi #2_6	432	576	1008
	Split_dSARM RNAi #2_7	562	334	896
	Split_OR_1	673	792	1465
	Split_OR_2	326	444	770
	Split_OR_3	482	239	721
	Split_OR_4	365	351	716

Split_OR_5	235	269	504
Split_OR_6	169	265	434
Split_OR_7	348	358	706

Brain Volume (um^3)				Normalized Dcp-1 Count		
Left Brain Lo	Right Brain L	Total		Left Brain Lo	Right Brain L	Total
1.08E+06	1.07E+06	2.15E+06		1.84E-04	2.68E-04	2.26E-04
9.59E+05	1.15E+06	2.11E+06		2.88E-04	1.45E-04	2.10E-04
1.22E+06	1.38E+06	2.60E+06		5.40E-04	2.49E-04	3.85E-04
9.80E+05	1.00E+06	1.98E+06		2.69E-04	4.85E-04	3.78E-04
1.76E+06	1.36E+06	3.12E+06		3.07E-04	1.35E-04	2.32E-04
1.16E+06	1.19E+06	2.35E+06		7.24E-05	1.61E-04	1.17E-04
1.36E+06	1.19E+06	2.55E+06		1.62E-04	1.87E-04	1.74E-04
1.61E+06	1.47E+06	3.08E+06		1.45E-04	1.46E-04	1.45E-04
1.36E+06	1.45E+06	2.81E+06		2.19E-04	2.48E-04	2.34E-04
1.46E+06	1.17E+06	2.63E+06		1.77E-04	1.37E-04	1.59E-04
1.59E+06	1.53E+06	3.12E+06		2.54E-04	2.37E-04	2.46E-04
1.84E+06	1.90E+06	3.74E+06		8.86E-05	1.72E-04	1.31E-04
1.47E+06	1.52E+06	2.99E+06		2.29E-04	1.99E-04	2.14E-04
1.43E+06	1.58E+06	3.01E+06		9.58E-05	1.28E-04	1.13E-04
1.26E+06	1.07E+06	2.33E+06		1.76E-04	1.34E-04	1.57E-04
8.63E+05	9.77E+05	1.84E+06		3.34E-04	1.81E-04	2.53E-04
8.74E+05	7.57E+05	1.63E+06		1.97E-04	3.08E-04	2.48E-04
7.53E+05	6.53E+05	1.41E+06		3.65E-04	3.57E-04	3.61E-04
8.38E+05	9.07E+05	1.75E+06		3.10E-04	2.80E-04	2.95E-04
1.51E+06	1.57E+06	3.08E+06		4.26E-04	2.96E-04	3.60E-04
9.41E+05	1.49E+06	2.43E+06		2.86E-04	2.20E-04	2.46E-04
7.72E+05	7.41E+05	1.51E+06		3.01E-04	3.71E-04	3.35E-04
1.26E+06	5.49E+05	1.81E+06		4.37E-04	5.63E-04	4.75E-04
1.65E+06	1.36E+06	3.01E+06		2.33E-04	2.29E-04	2.31E-04
1.28E+06	1.74E+06	3.02E+06		3.09E-04	3.76E-04	3.47E-04
1.99E+06	1.97E+06	3.96E+06		2.08E-04	3.72E-04	2.89E-04
1.07E+06	7.85E+05	1.86E+06		5.29E-04	5.72E-04	5.47E-04
1.67E+06	1.46E+06	3.13E+06		2.22E-04	3.81E-04	2.96E-04
1.38E+06	1.57E+06	2.95E+06		4.17E-04	3.39E-04	3.76E-04
8.75E+05	8.93E+05	1.77E+06		4.94E-04	6.45E-04	5.70E-04
1.18E+06	1.18E+06	2.36E+06		4.76E-04	2.83E-04	3.80E-04
1.26E+06	1.13E+06	2.39E+06		5.34E-04	7.01E-04	6.13E-04
9.18E+05	9.50E+05	1.87E+06		3.55E-04	4.67E-04	4.12E-04
1.29E+06	1.22E+06	2.51E+06		3.74E-04	1.96E-04	2.87E-04
1.21E+06	7.04E+05	1.91E+06		3.02E-04	4.99E-04	3.74E-04

1.13E+06	1.13E+06	2.26E+06		2.08E-04	2.38E-04	2.23E-04
7.67E+05	7.96E+05	1.56E+06		2.20E-04	3.33E-04	2.78E-04
1.47E+06	1.47E+06	2.94E+06		2.37E-04	2.44E-04	2.40E-04

Date		Dcp-1 Count		
4/12/21		Left Brain Lo	Right Brain L	Total
	Split_Ask1 ^N_1	221	228	449
	Split_Ask1 ^N_2	341	152	493
	Split_Ask1 ^N_3	260	231	491
	Split_Ask1 ^N_4	197	167	364
	Split_Ask1 ^N_5	414	357	771
	Split_Ask1 ^N_6	201	140	341
	Split_Ask1 ^N_7	166	263	429
	Split_Ask1 ^N_8	384	558	942
	Split_Ask1 FLAG_1	366	277	643
	Split_Ask1 FLAG_2	417	282	699
	Split_Ask1 FLAG_3	255	289	544
	Split_Ask1 FLAG_4	371	210	581
	Split_Ask1 FLAG_5	263	321	584
	Split_Ask1 FLAG_6	364	121	485
	Split_Ask1 FLAG_7	281	279	560
	Split_dSARM RNAi #2_1	474	496	970
	Split_dSARM RNAi #2_2	601	604	1205
	Split_dSARM RNAi #2_3	256	372	628
	Split_dSARM RNAi #2_4	553	606	1159
	Split_dSARM RNAi #2_5	823	751	1574
	Split_dSARM RNAi #2_6	624	626	1250
	Split_dSARM RNAi #2_7	713	592	1305
	Split_OR_1	288	378	666
	Split_OR_2	685	441	1126
	Split_OR_3	207	452	659
	Split_OR_4	236	154	390
	Split_OR_5	388	564	952
	Split_OR_6	296	379	675
	Split_OR_7	341	448	789
	Split_OR_8	359	450	809

Dcp-1 Volume			Brain Volume		
Left Brain Lo	Right Brain L	Total	Left Brain Lo	Right Brain L	Total
2693	3423	6116	1.48E+06	1.42E+06	2.90E+06
2757	1913	4670	1.23E+06	1.36E+06	2.59E+06
2996	2558	5554	1.19E+06	1.02E+06	2.21E+06
3201	1934	5135	8.24E+05	7.72E+05	1.60E+06
3709	3722	7431	1.15E+06	1.33E+06	2.48E+06
3862	2256	6118	1.12E+06	1.13E+06	2.25E+06
3252	3433	6685	1.02E+06	1.04E+06	2.06E+06
5647	4949	10596	1.38E+06	1.40E+06	2.78E+06
4010	3585	7595	1.19E+06	1.39E+06	2.58E+06
5494	5555	11049	1.19E+06	1.24E+06	2.43E+06
5098	4039	9137	1.13E+06	1.16E+06	2.29E+06
6114	2972	9086	1.28E+06	1.08E+06	2.36E+06
3050	2654	5704	1.19E+06	1.28E+06	2.47E+06
3646	1340	4986	1.24E+06	1.20E+06	2.44E+06
2877	3463	6340	1.45E+06	1.23E+06	2.68E+06
4999	8781	13780	6.42E+05	1.46E+06	2.10E+06
7467	9008	16475	1.70E+06	2.04E+06	3.74E+06
6221	7682	13903	1.22E+06	1.31E+06	2.53E+06
7601	8403	16004	1.39E+06	1.42E+06	2.81E+06
7696	9328	17024	1.36E+06	9.99E+05	2.36E+06
3418	4299	7717	1.64E+06	1.44E+06	3.08E+06
8787	6142	14929	1.34E+06	1.49E+06	2.83E+06
4655	2200	6855	1.48E+06	9.97E+05	2.48E+06
6159	6863	13022	1.88E+06	2.00E+06	3.88E+06
4608	5224	9832	1.60E+06	1.62E+06	3.22E+06
6258	3623	9881	1.43E+06	1.97E+06	3.40E+06
6789	3582	10371	1.22E+06	1.27E+06	2.49E+06
5414	6617	12031	1.30E+06	1.40E+06	2.70E+06
4010	4729	8739	1.92E+06	2.17E+06	4.09E+06
7378	3987	11365	1.63E+06	1.15E+06	2.78E+06

Normalized Dcp-1 Count		
Left Brain Lo	Right Brain L	Total
1.49E-04	1.61E-04	1.55E-04
2.77E-04	1.12E-04	1.90E-04
2.18E-04	2.26E-04	2.22E-04
2.39E-04	2.16E-04	2.28E-04
3.60E-04	2.68E-04	3.11E-04
1.79E-04	1.24E-04	1.52E-04
1.63E-04	2.53E-04	2.08E-04
2.78E-04	3.99E-04	3.39E-04
3.08E-04	1.99E-04	2.49E-04
3.50E-04	2.27E-04	2.88E-04
2.26E-04	2.49E-04	2.38E-04
2.90E-04	1.94E-04	2.46E-04
2.21E-04	2.51E-04	2.36E-04
2.94E-04	1.01E-04	1.99E-04
1.94E-04	2.27E-04	2.09E-04
7.38E-04	3.40E-04	4.61E-04
3.54E-04	2.96E-04	3.22E-04
2.10E-04	2.84E-04	2.48E-04
3.98E-04	4.27E-04	4.12E-04
6.05E-04	7.52E-04	6.67E-04
3.80E-04	4.35E-04	4.06E-04
5.32E-04	3.97E-04	4.61E-04
1.95E-04	3.79E-04	2.69E-04
3.64E-04	2.21E-04	2.90E-04
1.29E-04	2.79E-04	2.05E-04
1.65E-04	7.82E-05	1.15E-04
3.18E-04	4.44E-04	3.82E-04
2.28E-04	2.71E-04	2.50E-04
1.78E-04	2.06E-04	1.93E-04
2.20E-04	3.91E-04	2.91E-04

Date		Dcp-1 Count		
5/2/21		**Left Brain Lo**	**Right Brain L**	**Total**
	Split_Ask1 RNAi #2_1	557	642	1199
	Split_Ask1 RNAi #2_2	710	560	1270
	Split_Ask1 RNAi #2_3	829	711	1540
	Split_Ask1 RNAi #2_4	383	427	810
	Split_Ask1 RNAi #2_5	492	466	958
	Split_Ask1 RNAi #2_6	543	474	1017
	Split_Ask1 RNAi #2_7	504	626	1130
	Split_Ask1 RNAi #2_8	1057	876	1933
	Split_Ask1 RNAi #2_9	678	274	952
	Split_Ask1 RNAi #2_10	396	564	960
	Split_Ask1 RNAi #2_11	632	498	1130
	Split_Ask1 RNAi #2_12	911	763	1674
	Split_Ask1 RNAi #2_13	329	163	492
	Split_Ask1 RNAi #2_14	152	110	262
	Split_Ask1 RNAi #2_15	168	158	326
	Split_OR_1	119	155	274
	Split_OR_2	87	52	139
	Split_OR_3	67	49	116
	Split_OR_4	43	45	88

Dcp-1 Volume			Brain Volume		
Left Brain Lo	Right Brain L	Total	Left Brain Lo	Right Brain L	Total
8843	7693	16536	1.58E+06	1.46E+06	3.04E+06
1.01E+04	1.50E+04	25100	1.31E+06	1.70E+06	3.01E+06
7960	7937	15897	1.14E+06	1.18E+06	2.32E+06
6274	6633	12907	1.10E+06	1.11E+06	2.21E+06
7893	1.09E+04	18793	1.34E+06	1.50E+06	2.84E+06
7384	8847	16231	1.12E+06	1.40E+06	2.52E+06
6977	6104	13081	9.95E+05	9.73E+05	1.97E+06
1.42E+04	1.16E+04	25800	1.42E+06	1.60E+06	3.02E+06
9487	4514	14001	1.42E+06	1.38E+06	2.80E+06
6895	6866	13761	9.60E+05	9.90E+05	1.95E+06
1.06E+04	8526	19126	1.45E+06	1.40E+06	2.85E+06
1.36E+04	7799	21399	1.31E+06	1.67E+06	2.98E+06
1378.11	682.77	2060.88	1.25E+06	1.54E+06	2.79E+06
636.7	460.77	1097.47	5.97E+05	6.04E+05	1.20E+06
703.72	661.83	1365.55	7.11E+05	8.51E+05	1.56E+06
498.47	649.26	1147.73	1.29E+06	1.52E+06	2.81E+06
364.42	217.82	582.24	4.81E+05	5.27E+05	1.01E+06
280.65	205.25	485.9	3.63E+05	4.20E+05	7.83E+05
180.12	188.5	368.62	9.29E+05	9.42E+05	1.87E+06

Date		Punta Count			Brain lobe vo
11/8/21		L	R	T	L
	Ask1^6_1	204	256		7.58E+05
	Ask1^6_2	213	203		1.11E+06
	Ask1^6_3	43	57		3.00E+05
	Ask1^6_4	122	160		5.50E+05
	Ask1^6_5	157	83		4.57E+05
	Ask1^6_6	149	185		4.70E+05
	Ask1^6_7	181	170		5.20E+05
	Ask1^6_8	146	189		5.02E+05
	Ask1^6_9	169	146		8.79E+05
	Ask1^6_10	332	260		1.64E+06
	Ask1^6_11	180	171		9.91E+05
	Ask1^6_12	120	129		4.17E+05
	Ask1^6_13	216	394		1.04E+06
	Ask1^6_14	251	181		1.06E+06
	Ask1^6_15	62	76		3.16E+05
	Ask1^6_16	90	113		8.12E+05
	Ask1^6_17	269	315		1.42E+06
	OR_1	127	98		1.44E+06
	OR_2	48	20		4.16E+05
	OR_3	44	41		3.27E+05
	OR_4	41	24		3.70E+05
	OR_5	157	95		1.26E+06
	OR_6	28	34		4.12E+05
	OR_7	50	51		3.47E+05

lume		Normalized Count		
R	T	L	R	T
7.23E+05		0.00026913	0.00035408	
1.12E+06		0.00019189	0.00018125	
3.05E+05		0.00014333	0.00018689	
5.22E+05		0.00022182	0.00030651	
4.11E+05		0.00034354	0.00020195	
5.62E+05		0.00031702	0.00032918	
5.20E+05		0.00034808	0.00032692	
5.27E+05		0.00029084	0.00035863	
7.37E+05		0.00019226	0.0001981	
1.60E+06		0.00020244	0.0001625	
1.01E+06		0.00018163	0.00016931	
4.05E+05		0.00028812	0.00031852	
1.02E+06		0.00020769	0.00038627	
1.01E+06		0.00023679	0.0001801	
3.36E+05		0.0001962	0.00022619	
9.23E+05		0.00011084	0.00012243	
1.37E+06		0.00018944	0.00022993	
1.55E+06		8.8103E-05	6.31E-05	
4.26E+05		0.00011526	4.6992E-05	
3.14E+05		0.00013462	0.00013067	
3.84E+05		0.00011074	6.244E-05	
1.25E+06		0.00012438	7.5714E-05	
4.52E+05		6.7984E-05	7.5205E-05	
3.76E+05		0.00014422	0.00013578	

Date		Punta Count		Brain lobe volume	
12/20/21		L	R	L	R
	WT_1	139	196	1.16E+06	1.24E+06
	WT_2	66	96	4.51E+05	4.10E+05
	WT_3	82	20	2.90E+05	3.06E+05
	WT_4	68	35	4.55E+05	4.32E+05
	WT_5	117	208	1.27E+06	1.37E+06
	WT_6	57	79	4.06E+05	4.34E+05
	WT_7	98	72	5.24E+05	5.35E+05
	Ask1^6,dSARM KO_1	721	715	1.91E+06	1.80E+06
	Ask1^6,dSARM KO_2	276	212	7.97E+05	7.20E+05
	Ask1^6,dSARM KO_3	438	228	1.39E+06	1.35E+06
	Ask1^6,dSARM KO_4	180	302	1.11E+06	1.14E+06
	Ask1^6,dSARM KO_5	96	158	6.96E+05	6.65E+05

Normalized Count

L	R
0.00011995	0.00015819
0.00014649	0.00023393
0.00028287	6.5373E-05
0.0001494	8.0972E-05
9.1772E-05	0.00015234
0.00014042	0.00018193
0.00018694	0.00013461
0.00037788	0.00039656
0.00034626	0.00029444
0.00031579	0.00016939
0.00016165	0.00026538
0.00013797	0.00023777

Date		Punta Count		Brain Lobe V
12/21/21		L	R	L
	WT_1	166	125	1.09E+06
	WT_2	110	119	9.93E+05
	WT_3	154	175	1.23E+06
	WT_4	143	108	9.62E+05
	WT_5	212	160	8.14E+05
	WT_6	187	172	1.04E+06
	WT_7	110	92	8.38E+05
	WT_8	149	163	9.20E+05
	WT_9	92	70	6.77E+05
	Drpr-1/Ask1^6_1	182	172	9.97E+05
	Drpr-1/Ask1^6_2	128	89	7.12E+05
	Drpr-1/Ask1^6_3	76	123	8.52E+05
	Drpr-1/Ask1^6_4	151	135	1.22E+06
	Drpr-1/Ask1^6_5	228	106	1.20E+06
	Drpr-1/Ask1^6_6	96	123	9.56E+05
	Drpr-1/Ask1^6_7	133	161	1.25E+06
	Drpr-1/Ask1^6_8	152	153	9.09E+05
	Drpr-1/Ask1^6_9	136	150	7.57E+05
	Drpr-1/Ask1^6_10	132	86	6.01E+05
	Drpr-1/Ask1^6_11	104	179	9.73E+05
	Ask1^6,dSARM KO_1	576	621	9.32E+05
	Ask1^6,dSARM KO_2	203	191	3.85E+05
	Ask1^6,dSARM KO_3	94	114	3.73E+05
	Ask1^6,dSARM KO_4	73	74	2.42E+05
	Ask1^6,dSARM KO_5	251	241	8.52E+05
	Ask1^6,dSARM KO_6	188	197	6.92E+05
	Ask1^6,dSARM KO_7	87	80	2.35E+05

olume	Normalized Count	
R	L	R
9.55E+05	0.00015261	0.00013088
9.47E+05	0.00011073	0.00012566
1.18E+06	0.00012561	0.00014894
8.94E+05	0.00014859	0.00012081
6.74E+05	0.00026033	0.00023752
1.13E+06	0.00018029	0.00015269
8.04E+05	0.00013124	0.00011442
9.12E+05	0.00016194	0.00017867
6.74E+05	0.00013585	0.00010382
1.10E+06	0.00018256	0.00015606
7.50E+05	0.00017971	0.00011873
8.46E+05	8.9214E-05	0.00014544
1.23E+06	0.00012355	0.00010941
1.31E+06	0.00018961	8.0735E-05
9.21E+05	0.00010037	0.0001335
1.26E+06	0.00010603	0.00012768
8.53E+05	0.0001672	0.00017933
8.72E+05	0.00017955	0.000172
5.49E+05	0.00021964	0.00015676
1.08E+06	0.00010687	0.00016498
9.42E+05	0.0006177	0.0006591
4.09E+05	0.00052761	0.00046668
3.60E+05	0.00025183	0.00031644
2.50E+05	0.0003017	0.00029634
7.17E+05	0.00029473	0.00033607
6.71E+05	0.00027157	0.00029349
2.54E+05	0.00037058	0.00031477

Date		Punta Count		Brain Lobe Volume	
2/27/22		L	R	L	R
	Ask1^6_1	44	66	2.56E+05	2.75E+05
	Ask1^6_2	111	56	3.52E+05	3.87E+05
	Ask1^6_3	362	400	1.13E+06	1.12E+06
	Ask1^6_4	120	122	4.64E+05	4.84E+05
	Ask1^6_5	33	71	2.06E+05	2.74E+05
	attp KO_1	148	185	4.83E+05	4.38E+05
	attp KO_2	286	427	1.10E+06	1.09E+06
	attp KO_3	99	107	2.96E+05	3.29E+05
	attp KO_4	89	121	3.65E+05	3.82E+05
	attp KO_5	266	150	8.64E+05	8.79E+05
	Ask1^6,dSARM KO_1	157	292	1.03E+06	9.05E+05
	Ask1^6,dSARM KO_2	261	268	9.58E+05	9.31E+05
	Ask1^6,dSARM KO_3	215	147	6.82E+05	7.33E+05
	Ask1^6,dSARM KO_4	80	87	4.68E+05	3.83E+05
	Ask1^6,dSARM KO_5	70	31	2.68E+05	2.56E+05
	Ask1^6,dSARM KO_6	59	85	2.04E+05	1.83E+05

Normalized Count

L	R
0.00017181	0.00024
0.00031516	0.00014482
0.00032149	0.00035778
0.00025884	0.00025207
0.00016019	0.00025912
0.00030667	0.00042237
0.00026119	0.00039103
0.00033503	0.00032552
0.00024417	0.00031675
0.00030805	0.00017057
0.00015228	0.00032262
0.00027253	0.00028786
0.0003153	0.00020063
0.00017109	0.00022721
0.00026119	0.00012114
0.00028964	0.00046499

DATE		Punta Count		Brain lobe vo
3/8/22		L	R	L
	dSARM Rescue/+_1	103	139	8.37E+05
	dSARM Rescue/+_2	234	122	1.01E+06
	dSARM Rescue/+_3	158	138	8.71E+05
	dSARM Rescue/+_4	279	138	1.18E+06
	dSARM Rescue/+_5	101	113	7.90E+05
	dSARM Rescue/+_6	98	200	9.27E+05
	dSARM Rescue/+_7	177	363	1.29E+06
	dSARM SAM/+_1	168	184	9.75E+05
	dSARM SAM/+_2	92	131	8.90E+05
	dSARM SAM/+_3	117	279	1.02E+06
	dSARM SAM/+_4	144	218	1.12E+06
	dSARM SAM/+_5	121	186	6.85E+05
	dSARM SAM/+_6	104	171	8.85E+05
	dSARM SAM/+_7	148	188	9.98E+05
	dSARM SAM/+_8	117	98	7.83E+05
	dSARM SAM/+_9	78	99	7.00E+05
	dSARM SAM/+_10	96	83	7.48E+05
	dSARM TIR/+_1	81	78	5.33E+05
	dSARM TIR/+_2	67	90	5.65E+05
	dSARM TIR/+_3	78	80	4.40E+05
	dSARM TIR/+_4	313	185	7.36E+05
	dSARM TIR/+_5	243	233	5.86E+05
	dSARM TIR/+_6	184	213	6.92E+05
	dSARM TIR/+_7	106	99	6.63E+05
	dSARM TIR/+_8	129	108	7.82E+05
	dSARM TIR/+_9	150	383	8.98E+05
	dSARM KO/+_1	100	93	5.72E+05
	dSARM KO/+_2	103	85	8.19E+05
	dSARM KO/+_3	159	136	8.47E+05
	dSARM KO/+_4	115	89	8.78E+05
	dSARM KO/+_5	85	135	6.90E+05
	dSARM KO/+_6	139	94	8.60E+05
	dSARM KO/+_7	114	127	9.46E+05
	dSARM KO/+_8	92	103	6.71E+05
	dSARM KO/+_9	77	184	6.14E+05
	dSARM KO/+_10	108	132	7.57E+05
	dSARM KO/+_11	105	115	6.46E+05

dSARM E1170A/+_1	277	188	1.24E+06
dSARM E1170A/+_2	108	138	1.03E+06
dSARM E1170A/+_3	96	145	8.90E+05
dSARM E1170A/+_4	124	217	9.02E+05
dSARM E1170A/+_5	190	188	7.93E+05
dSARM E1170A/+_6	90	112	1.03E+06
dSARM E1170A/+_7	416	117	7.24E+05
dSARM E1170A/+_8	101	79	6.15E+05
dSARM E1170A/+_9	199	88	6.50E+05
dSARM E1170A/+_10	69	87	4.63E+05
dSARM ARM-TIR/+_1	36	67	2.93E+05
dSARM ARM-TIR/+_2	85	44	3.41E+05
dSARM ARM-TIR/+_3	31	37	3.06E+05
dSARM ARM-TIR/+_4	47	93	4.36E+05
dSARM ARM-TIR/+_5	54	45	3.80E+05
dSARM ARM-TIR/+_6	87	40	5.32E+05
dSARM ARM-TIR/+_7	123	51	6.13E+05
dSARM ARM-TIR/+_8	67	49	4.45E+05
dSARM ARM-TIR/+_9	43	60	4.66E+05
dSARM ARM-SAM/+_1	151	130	1.09E+06
dSARM ARM-SAM/+_2	143	133	6.80E+05
dSARM ARM-SAM/+_3	104	100	6.34E+05
dSARM ARM-SAM/+_4	94	87	5.77E+05

olume	Normalized Count	
R	L	R
7.38E+05	0.00012303	0.0001884
9.90E+05	0.00023214	0.00012319
8.55E+05	0.0001814	0.00016142
1.11E+06	0.00023624	0.00012466
7.74E+05	0.00012791	0.00014601
9.23E+05	0.00010573	0.00021678
1.19E+06	0.00013732	0.0003053
9.63E+05	0.00017233	0.00019105
8.29E+05	0.00010341	0.00015806
9.64E+05	0.00011448	0.00028942
1.07E+06	0.00012857	0.0002045
9.35E+05	0.00017672	0.00019885
8.62E+05	0.00011758	0.00019828
9.52E+05	0.00014824	0.00019758
8.28E+05	0.0001495	0.00011833
6.91E+05	0.00011138	0.00014325
7.31E+05	0.00012836	0.00011353
5.91E+05	0.000152	0.00013198
6.73E+05	0.00011861	0.00013381
4.45E+05	0.00017719	0.00017986
7.50E+05	0.0004255	0.00024667
5.97E+05	0.00041482	0.00039055
7.16E+05	0.00026601	0.00029769
6.17E+05	0.00015981	0.00016056
7.86E+05	0.00016498	0.00013742
1.33E+06	0.00016696	0.00028819
5.61E+05	0.00017486	0.00016575
7.43E+05	0.00012581	0.00011439
9.43E+05	0.00018779	0.00014416
8.90E+05	0.00013105	0.0001
7.63E+05	0.00012312	0.00017686
8.86E+05	0.0001617	0.00010605
1.02E+06	0.00012052	0.00012439
6.33E+05	0.00013713	0.00016277
7.12E+05	0.00012533	0.00025857
7.86E+05	0.00014261	0.00016802
8.57E+05	0.00016261	0.0001342

1.25E+06	0.00022267	0.00015016
1.05E+06	0.00010445	0.00013155
9.34E+05	0.00010793	0.0001553
9.78E+05	0.00013744	0.0002219
7.67E+05	0.00023954	0.00024521
1.02E+06	8.7634E-05	0.00011034
7.25E+05	0.0005749	0.00016149
5.96E+05	0.00016428	0.00013259
6.31E+05	0.00030601	0.00013937
4.63E+05	0.00014909	0.00018786
3.37E+05	0.00012278	0.00019905
3.51E+05	0.00024949	0.00012521
3.60E+05	0.00010124	0.00010289
4.60E+05	0.00010785	0.00020235
3.53E+05	0.00014211	0.00012737
5.12E+05	0.00016363	7.8201E-05
6.05E+05	0.00020078	8.4339E-05
4.60E+05	0.00015063	0.00010661
4.98E+05	9.2275E-05	0.00012048
1.02E+06	0.00013879	0.00012783
6.45E+05	0.00021039	0.00020617
6.49E+05	0.00016393	0.00015418
5.70E+05	0.00016302	0.00015263

Date		Punta Count		Brain lobe vo
3/12/22		L	R	L
	dSARM ARM-SAM/+_1	173	172	1.41E+06
	dSARM ARM-SAM/+_2	92	132	1.05E+06
	dSARM ARM-SAM/+_3	128	60	8.01E+05
	dSARM ARM-SAM/+_4	109	127	7.76E+05
	dSARM ARM-SAM/+_5	94	96	8.59E+05
	dSARM ARM-SAM/+_6	149	75	6.61E+05
	dSARM ARM-SAM/+_7	176	76	5.62E+05
	dSARM ARM-SAM/+_8	148	84	6.01E+05
	dSARM ARM-TIR/+_1	165	51	8.13E+05
	dSARM ARM-TIR/+_2	57	144	7.70E+05
	dSARM ARM-TIR/+_3	103	78	6.71E+05
	dSARM ARM-TIR/+_4	60	110	5.72E+05
	dSARM KO/+_1	100	119	1.36E+06
	dSARM KO/+_2	179	149	8.39E+05
	dSARM KO/+_3	122	112	6.13E+05
	dSARM KO/+_4	85	90	7.66E+05
	dSARM KO/+_5	121	101	8.49E+05
	dSARM KO/+_6	116	98	8.71E+05
	dSARM KO/+_7	91	121	7.77E+05
	dSARM KO/+_8	165	150	9.92E+05
3/16/22	dSARM E1170A/+_1	121	93	6.48E+05
	dSARM E1170A/+_2	brain damaged		
	dSARM E1170A/+_3	179	52	7.38E+05
	dSARM E1170A/+_4	overexposed		
	dSARM E1170A/+_5	120	69	6.96E+05
	dSARM E1170A/+_6	brain damaged		
	dSARM E1170A/+_7	brain damaged		
	dSARM E1170A/+_8	64	53	6.76E+05
	dSARM TIR/+_1	damaged		
	dSARM TIR/+_2	136	29	5.06E+05
	dSARM TIR/+_3	62	53	5.41E+05
	dSARM TIR/+_4	51	45	4.75E+05
	dSARM TIR/+_5	68	74	4.81E+05
	dSARM TIR/+_6	45	66	3.60E+05
	dSARM TIR/+_7	61	125	5.43E+05
	dSARM TIR/+_8	damaged		

dSARM TIR/+_9	damaged		
dSARM SAM/+_1	135	50	6.30E+05
dSARM SAM/+_2	109	94	7.29E+05
dSARM SAM/+_3	102	122	6.33E+05
dSARM SAM/+_4	damaged		
dSARM SAM/+_5	117	112	6.58E+05
dSARM SAM/+_6	77	105	7.98E+05
dSARM SAM/+_7	99	102	4.97E+05
dSARM Rescue/+_1	163	110	5.31E+05
dSARM Rescue/+_2	133	52	5.17E+05
dSARM Rescue/+_3	64	72	8.89E+05
dSARM Rescue/+_4	109	115	7.07E+05
dSARM Rescue/+_5	49	65	6.50E+05
dSARM Rescue/+_6	81	104	8.15E+05
dSARM Rescue/+_7	63	98	8.94E+05
dSARM Rescue/+_8	68	120	6.32E+05
dSARM Rescue/+_9	133	126	1.05E+06

lume	Normalized Count	
R	L	R
1.25E+06	0.00012243	0.00013738
1.03E+06	8.7703E-05	0.00012803
7.26E+05	0.00015984	8.2667E-05
8.11E+05	0.00014041	0.00015662
8.76E+05	0.0001094	0.00010956
7.23E+05	0.00022555	0.00010381
7.77E+05	0.00031306	9.7808E-05
5.51E+05	0.00024634	0.00015242
7.98E+05	0.00020288	6.3878E-05
7.55E+05	7.4026E-05	0.00019065
6.79E+05	0.00015357	0.00011494
7.37E+05	0.00010495	0.00014936
1.17E+06	7.3529E-05	0.00010154
8.69E+05	0.0002134	0.00017156
5.52E+05	0.00019902	0.00020294
8.14E+05	0.00011097	0.00011062
8.89E+05	0.00014259	0.00011367
8.65E+05	0.0001332	0.00011324
7.32E+05	0.00011712	0.00016535
9.28E+05	0.00016633	0.00016159
6.58E+05	0.00018684	0.00014134
6.94E+05	0.00024271	7.4928E-05
6.80E+05	0.00017241	0.00010143
7.06E+05	9.4689E-05	7.5092E-05
4.53E+05	0.00026877	6.4088E-05
5.78E+05	0.00011454	9.1759E-05
4.79E+05	0.00010737	9.3946E-05
4.38E+05	0.00014131	0.00016903
3.65E+05	0.00012507	0.00018092
6.00E+05	0.00011238	0.00020851

6.30E+05	0.00021442	7.9352E-05
6.60E+05	0.00014948	0.00014253
6.10E+05	0.00016104	0.00019993
5.79E+05	0.00017778	0.0001935
7.08E+05	9.6491E-05	0.00014833
5.05E+05	0.00019928	0.00020218
5.73E+05	0.00030726	0.00019214
4.70E+05	0.0002575	0.00011066
8.10E+05	7.1967E-05	8.8878E-05
5.72E+05	0.00015409	0.00020108
5.56E+05	7.5443E-05	0.00011686
7.49E+05	9.9338E-05	0.00013889
8.16E+05	7.0509E-05	0.00012011
5.78E+05	0.00010766	0.00020772
9.07E+05	0.00012619	0.000139

Date		Punta Count		
8/5/21		L	R	T
	dSARM KO_1	289	196	485
	dSARM KO_2	84	89	173
	dSARM Rescue_1	88	181	269
	dSARM Rescue_2	136	151	287
	dSARM Rescue_3	117	198	315
	dSARM Rescue_4	219	227	446
	dSARM Rescue_5	105	90	195
	dSARM Rescue_6	218	177	395

Volume				Brain lobe volume		
L	R	T		L	R	T
5798.68	4334.88	10133.56		1070700.00	1149600.00	2220300.00
1668.04	1666.89	3334.93		3.56E+05	2.44E+05	599595.00
1966.61	2982.56	4949.17		1.21E+06	1.27E+06	2.49E+06
2518.81	2664.8	5183.61		7.76E+05	9.30E+05	1.71E+06
2634.81	3104.87	5739.68		8.60E+05	8.95E+05	1.75E+06
3623.48	4139.1	7762.58		1.08E+06	9.78E+05	2.06E+06
2135.57	1909.11	4044.68		1.05E+06	1.17E+06	2.22E+06
3720.33	2924.14	6644.47		1.64E+06	1.35E+06	3.00E+06

Normalized Count			Normalized Volume	
L	R	T	L	R
0.00026992	0.00017049	0.00021844	0.00541578	0.00377077
0.00023605	0.00036515	0.00028853	0.00468734	0.00683897
7.2506E-05	0.00014223	0.00010819	0.00162036	0.00234367
0.00017529	0.00016232	0.00016822	0.00324655	0.00286449
0.00013612	0.00022125	0.00017954	0.00306538	0.0034695
0.00020241	0.00023212	0.00021652	0.00334903	0.00423249
9.9719E-05	7.6987E-05	8.7759E-05	0.00202816	0.00163307
0.00013274	0.00013074	0.00013184	0.00226529	0.0021599

T
0.00456405
0.00556197
0.00199058
0.00303823
0.00327151
0.00376845
0.0018203
0.00221767

Date		Punta Count			Volume
8/7/21		L	R	T	L
	attP KO_1	101	115	216	1756.43
	attP KO_2	250	78	328	4332.16
	attP KO_3	89	96	185	1570.46
	attP KO_4	137	94	231	2131.5
	attP KO_5	244	216	460	3696.61
	attP KO_6	152	128	280	2453.56
	Rescue_1	56	58	114	1075.08
	Rescue_2	65	94	159	1242.18
	Rescue_3	46	35	81	870.714
	Rescue_4	63	78	141	1204.82
	Rescue_5	329	82	411	5714.99
	Rescue_6	94	65	159	1821.32

		Brain lobe volume		
R	T	L	R	T
1999.92	3756.35	3.45E+05	3.02E+05	6.47E+05
1416.57	5748.73	1.01E+06	3.44E+05	1.35E+06
1777.54	3348	3.31E+05	3.32E+05	6.63E+05
1533.85	3665.35	3.92E+05	4.33E+05	8.26E+05
3740.36	7436.97	9.60E+05	9.31E+05	1.89E+06
2352.2	4805.76	4.47E+05	4.57E+05	9.04E+05
1086.47	2161.55	3.54E+06	3.53E+05	3.89E+06
1787.566	3029.746	3.20E+05	2.97E+05	6.17E+05
828.672	1699.386	1.86E+05	4.27E+05	6.13E+05
1531.23	2736.05	5.52E+05	5.00E+05	1.05E+06
1531.41	7246.4	1.20E+06	7.53E+05	1.95E+06
1217.26	3038.58	1.04E+06	1.04E+06	2.09E+06

Normalized Count

L	R	T
0.00029266	0.00038128	0.00033399
0.00024746	0.00022706	0.00024229
0.00026872	0.00028894	0.00027884
0.00034906	0.00021694	0.00027973
0.00025417	0.00023201	0.00024326
0.00034005	0.00028009	0.00030973
1.5837E-05	0.00016445	2.93E-05
0.00020285	0.00031645	0.0002575
0.00024716	8.2018E-05	0.00013217
0.00011403	0.00015586	0.00013391
0.0002737	0.00010894	0.00021026
9.0047E-05	6.2383E-05	7.6228E-05

Normalized Volume

L	R	T
0.00508954	0.00663062	0.00580826
0.00428821	0.0041237	0.00424646
0.00474168	0.00534999	0.00504632
0.00543078	0.00353993	0.00443863
0.00385064	0.00401757	0.00393282
0.00548895	0.00514705	0.00531611
0.00030403	0.00308052	0.00055584
0.00387648	0.00601787	0.00490661
0.00467837	0.00194188	0.00277291
0.0021807	0.00305964	0.00259845
0.00475445	0.00203454	0.0037071
0.00174473	0.00116825	0.00145676

Date		Punta Count				Volume
8/12/21		L	R	T		L
	attP KO_1	187	164	351		3120.15
	attP KO_2	214	249	463		3765.6
	attP KO_3	278	203	481		4633.04
	attP KO_4	178	302	480		2625.23
	attP KO_5	93	126	219		1481.21
	attP KO_6	333	371	704		6390.45
	attP KO_7	179	200	379		3274.8
	Rescue_1	118	70	188		2239.11
	Rescue_2	131	85	216		2477.06
	Rescue_3	112	68	180		2285.43
	Rescue_4	62	49	111		1096.79
	Rescue_5	66	63	129		1160.82
	Rescue_6	63	51	114		1193.61
	Rescue_7	48	36	84		869.251

Brain lobe volume

R	T	L	R	T
3045.8	6165.95	7.93E+05	8.98E+05	1.69E+06
3546.85	7312.45	8.50E+05	8.75E+05	1.72E+06
3476.26	8109.3	8.60E+05	8.66E+05	1.73E+06
5445.54	8070.77	4.57E+05	1.17E+06	1.63E+06
2032.81	3514.02	2.93E+05	4.40E+05	7.33E+05
7010.97	13401.42	1.23E+06	1.26E+06	2.49E+06
3680.52	6955.32	8.52E+05	9.18E+05	1.77E+06
1288.31	3527.42	4.87E+05	5.03E+05	9.90E+05
1972.06	4449.12	4.22E+05	4.71E+05	8.93E+05
1436.42	3721.85	4.04E+05	5.73E+05	9.77E+05
935.428	2032.218	2.82E+05	3.22E+05	6.04E+05
1193.13	2353.95	5.39E+05	5.96E+05	1.14E+06
1125	2318.61	3.88E+05	4.49E+05	8.37E+05
691.352	1560.603	3.98E+05	4.44E+05	8.42E+05

Normalized Count

L	R	T
0.00023592	0.00018256	0.00020757
0.00025168	0.0002847	0.00026842
0.00032326	0.00023433	0.00027863
0.00038992	0.00025812	0.00029511
0.00031741	0.00028656	0.00029889
0.00027073	0.00029444	0.00028273
0.00021009	0.00021786	0.00021412
0.00024222	0.00013907	0.0001898
0.00031044	0.00018061	0.00024198
0.00027725	0.00011873	0.00018429
0.00021947	0.00015218	0.00018363
0.00012242	0.00010562	0.0001136
0.00016218	0.00011366	0.00013617
0.00012061	8.1008E-05	9.9719E-05

Normalized Volume

L	R	T
0.00393634	0.00339048	0.00364635
0.00442855	0.0040554	0.00423935
0.00538726	0.00401277	0.0046975
0.00575078	0.00465431	0.00496205
0.00505532	0.00462317	0.00479599
0.00519549	0.00556426	0.0053821
0.00384366	0.00400928	0.00392956
0.00459618	0.00255959	0.00356127
0.00586999	0.00419019	0.00498432
0.00565751	0.00250797	0.00381061
0.00388254	0.00290526	0.00336198
0.00215315	0.00200032	0.00207288
0.0030727	0.0025072	0.0027696
0.00218423	0.00155569	0.00185264

Date		Left Brain Lo	Right Brain L	Total
8/18/21	dSarm Rescue_1	123	119	242
	dSarm Rescue_2	81	115	196
	dSarm Rescue_3	83	145	228
	dSarm Rescue_4	88	150	238
	dSarm Rescue_5	106	96	202
	dSarm Rescue_6	99	109	208
	dSarm Rescue_7	114	126	240
	dSarm Rescue_8	76	100	176
	dSarm Rescue_9	85	102	187
	ΔSAM_ΔTIR ΔARM_1	125	137	262
	ΔSAM_ΔTIR ΔARM_2	118	195	313
	ΔSAM_ΔTIR ΔARM_3	206	128	334
	ΔSAM_ΔTIR ΔARM_4	114	138	252
	ΔSAM_ΔTIR ΔARM_5	93	61	154
	ΔSAM_ΔTIR ΔARM_6	53	111	164
	ΔSAM_ΔTIR ΔARM_7	66	167	233
	ΔSAM_ΔTIR ΔARM_8	219	314	533
	ΔTIR_ΔARM ΔSAM_1	147	187	334
	ΔTIR_ΔARM ΔSAM_2	65	97	162
	ΔTIR_ΔARM ΔSAM_3	170	147	317
	ΔTIR_ΔARM ΔSAM_4	45	51	96
	ΔTIR_ΔARM ΔSAM_5	328	331	659
	ΔTIR_ΔARM ΔSAM_6	116	89	205
	ΔTIR_ΔARM ΔSAM_7	173	149	322
	ΔTIR_ΔARM ΔSAM_8	89	117	206

Left Brain Lo	Right Brain L	Total
515.22	498.47	1013.69
339.29	481.71	821
347.67	607.38	955.05
368.61	628.32	996.93
444.01	402.12	846.13
414.69	456.58	871.27
477.52	527.79	1005.31
318.35	418.88	737.23
356.05	427.26	783.31
523.6	573.86	1097.46
494.28	816.81	1311.09
862.89	536.16	1399.05
477.52	578.05	1055.57
389.56	255.52	645.08
222.01	464.96	686.97
276.46	699.53	975.99
917.34	1315.28	2232.62
615.75	783.3	1399.05
272.27	406.31	678.58
712.09	615.75	1327.84
188.5	213.63	402.13
1373.92	1386.49	2760.41
485.9	372.8	858.7
724.66	624.13	1348.79
372.8	490.09	862.89

Left Brain Lo	Right Brain L	Total
5.98E+05	6.82E+05	1.28E+06
1.01E+06	1.02E+06	2.03E+06
1.44E+06	1.63E+06	3.07E+06
8.23E+05	7.49E+05	1.57E+06
6.84E+05	7.71E+05	1.46E+06
4.62E+05	4.82E+05	9.44E+05
6.63E+05	8.36E+05	1.50E+06
5.41E+05	6.00E+05	1.14E+06
4.07E+05	4.22E+05	8.29E+05
1.67E+06	1.58E+06	3.25E+06
1.12E+06	1.04E+06	2.16E+06
1.18E+06	9.41E+05	2.12E+06
1.02E+06	1.09E+06	2.11E+06
5.34E+05	4.92E+05	1.03E+06
7.55E+05	7.36E+05	1.49E+06
1.23E+06	1.16E+06	2.39E+06
1.25E+06	1.45E+06	2.70E+06
1.05E+06	1.30E+06	2.35E+06
9.67E+05	8.95E+05	1.86E+06
4.30E+05	3.81E+05	8.11E+05
4.10E+05	3.11E+05	7.21E+05
1.06E+06	1.16E+06	2.22E+06
6.54E+05	6.38E+05	1.29E+06
8.41E+05	7.58E+05	1.60E+06
7.39E+05	9.17E+05	1.66E+06

Date		Dcp-1 Count			Brain Volum
9/23/21		**L**	**R**	**Total**	**L**
	dSARM Rescue_1	61	43	104	6.17E+05
	dSARM Rescue_2	123	44	167	4.31E+05
	dSARM Rescue_3	34	27	61	4.38E+05
	dSARM Rescue_4	51	59	110	3.48E+05
	dSARM Rescue_5	47	56	103	4.23E+05
	dSARM Rescue_6	111	136	247	6.00E+05
	dSARM Rescue_7	23	36	59	1.99E+05
	dSARM Rescue_8	70	55	125	3.69E+05
	dSARM Rescue_9	50	55	105	3.82E+05
	dSARM E1170A_4_1	155	258	413	1.18E+06
	dSARM E1170A_4_2	119	63	182	3.09E+05
	dSARM E1170A_4_3	145	131	276	4.81E+05
	dSARM E1170A_4_4	445	372	817	1.23E+06
	dSARM E1170A_4_5	332	468	800	1.31E+06
	dSARM E1170A_4_6	35	36	71	2.50E+05
	dSARM E1170A_4_7	83	87	170	3.14E+05
	dSARM E1170A_4_8	55	37	92	1.92E+05
	dSARM E1170A_4_9	63	44	107	2.16E+05
	dSARM E1170A_4_10	48	43	91	1.15E+05
	dSARM E1170A_4_11	37	55	92	7.78E+04
	dSARM E1170A_4_12	54	41	95	1.74E+05
	dSARM E1170A_4_13	37	45	82	2.80E+05
	dSARM E1170A_4_14	34	70	104	1.21E+05

e		Norm Brain Count		
R	Total	L	R	Total
5.43E+05	1.16E+06	9.88365E-05	7.92583E-05	8.96775E-05
4.41E+05	8.72E+05	0.000285184	9.98185E-05	0.000191492
4.10E+05	8.48E+05	7.77143E-05	6.58537E-05	7.19764E-05
3.27E+05	6.75E+05	0.000146599	0.000180257	0.000162915
4.20E+05	8.42E+05	0.000111181	0.000133416	0.000122259
6.56E+05	1.26E+06	0.000185	0.000207317	0.000196656
1.80E+05	3.79E+05	0.00011552	0.000199601	0.000155484
3.74E+05	7.43E+05	0.000189809	0.000147095	0.000168305
4.07E+05	7.89E+05	0.00013089	0.000135135	0.00013308
1.06E+06	2.24E+06	0.000131356	0.000243626	0.000184457
1.57E+05	4.66E+05	0.000385113	0.000401274	0.000390558
4.78E+05	9.59E+05	0.000301455	0.000274059	0.0002878
1.48E+06	2.71E+06	0.0003618	0.000251351	0.00030148
1.29E+06	2.60E+06	0.000253823	0.000362791	0.000307929
3.04E+05	5.54E+05	0.00014	0.000118421	0.000128159
2.91E+05	6.05E+05	0.000264331	0.000298969	0.000280992
1.93E+05	3.85E+05	0.000286458	0.00019171	0.000238961
2.41E+05	4.57E+05	0.000291667	0.000182573	0.000234136
1.33E+05	2.48E+05	0.000417391	0.000323308	0.000366935
9.98E+04	1.78E+05	0.000475578	0.000551102	0.000518018
1.49E+05	3.23E+05	0.000310345	0.000275168	0.000294118
2.40E+05	5.20E+05	0.000132143	0.0001875	0.000157692
1.51E+05	2.72E+05	0.000280992	0.000463576	0.000382353

Date		Punta Count		
10/20/21		L	R	T
	dSARM Rescue_1	63	81	
	dSARM Rescue_2	55	58	
	dSARM Rescue_3	60	41	
	dSARM Rescue_4	24	15	
	dSARM Rescue_5	146	114	
	dSARM Rescue_6	65	82	
	dSARM SAM_1	42	38	
	dSARM SAM_2	132	127	
	dSARM SAM_3	152	142	
	dSARM SAM_4	52	54	
	dSARM ARM-TIR_1	77	63	
	dSARM ARM-TIR_2	61	53	
	dSARM ARM-TIR_3	95	111	
	dSARM ARM-TIR_4	42	46	
	dSARM ARM-TIR_5	31	20	
	dSARM ARM-TIR_6	42	25	
	dSARM ARM-TIR_7	34	52	
	dSARM ARM-SAM_1	35	25	
	dSARM ARM-SAM_2	203	113	
	dSARM ARM-SAM_3	20	38	
	dSARM ARM-SAM_4	42	39	
	dSARM ARM-SAM_5	41	48	
	dSARM ARM-SAM_6	16	47	

Volume

L	R	T
944.974	1351.08	
1001.6	1196.05	
1132.4	881.7	
429.666	318.037	
2634.81	2336.72	
1116.83	1310.11	
740.012	705.367	
2198.25	2233.57	
2007.53	1841.21	
497.435	707.626	
1356.83	1036.84	
1058.35	811.25	
1593.41	1832.04	
787.778	787.021	
594.718	363.193	
944.3	649.698	
606.88	812.231	
370.016	300.911	
2404.44	1785.03	
384.214	693.206	
625.567	481.924	
626.641	768.041	
284.949	579.539	

Brain lobe volume

L	R	T
3.46E+05	3.46E+05	
5.36E+05	5.07E+05	
2.74E+05	2.74E+05	
1.12E+05	1.35E+05	
1.51E+06	1.19E+06	
7.77E+05	7.67E+05	
2.77E+05	2.46E+05	
5.88E+05	5.96E+05	
6.09E+05	6.80E+05	
1.22E+05	1.34E+05	
7.63E+05	8.21E+05	
4.01E+05	4.23E+05	
5.21E+05	4.21E+05	
2.06E+05	2.00E+05	
1.41E+05	1.45E+05	
4.67E+05	4.85E+05	
1.08E+05	1.21E+05	
1.12E+05	1.34E+05	
4.35E+05	4.46E+05	
1.04E+05	1.18E+05	
1.83E+05	1.96E+05	
1.66E+05	1.85E+05	
8.71E+04	1.05E+05	

Normalized Count			Normalized Volume	
L	R	T	L	R
0.00018208	0.0002341		0.00273114	0.00390486
0.00010261	0.0001144		0.00186866	0.00235907
0.00021938	0.00014955		0.0041404	0.00321602
0.00021467	0.00011111		0.00384317	0.00235583
9.6689E-05	9.5879E-05		0.00174491	0.00196528
8.3655E-05	0.00010695		0.00143736	0.00170876
0.00015162	0.00015447		2.67E-03	0.00286735
0.00022449	0.00021309		0.00373852	0.0037476
0.00024959	0.00020879		0.00329644	0.00270726
0.00042623	0.00040179		0.00407734	0.00526507
0.00010092	7.6736E-05		0.00177828	0.0012629
0.00015212	0.0001253		0.00263928	0.00191785
0.00018234	0.00026366		0.00305837	0.00435164
0.00020388	0.00023		0.00382417	0.00393511
0.00021986	0.00013793		0.00421786	0.00250478
8.9936E-05	5.1546E-05		0.00202206	0.00133958
0.00031481	0.00042975		0.00561926	0.00671265
0.0003134	0.00018601		0.00331318	0.00223892
0.00046688	0.00025336		0.00552999	0.00400231
0.00019231	0.00032203		0.00369437	0.00587463
0.00023001	0.00019939		0.00342589	0.00246382
0.00024773	0.00025904		0.00378635	0.00414485
0.0001837	0.00044762		0.00327152	0.00551942

T

Date		Punta Count		
10/25/21		L	R	T
	dSARM ARMSAM_1	31	14	
	dSARM ARMSAM_2	41	36	
	dSARM ARMSAM_3	31	20	
	dSARM ARMSAM_4	19	26	
	dSARM Rescue_1	67	48	
	dSARM Rescue_2	68	186	
	dSARM Rescue_3	70	79	
	dSARM Rescue_4	201	209	
	dSARM Rescue_5	59	68	
	dSARM Rescue_6	27	86	
	dSARM SAM_1	72	39	
	dSARM SAM_2	12	29	
	dSARM SAM_3	45	38	
	dSARM SAM_4	20	17	
	dSARM SAM_5	167		
	dSARM ARM-TIR_1	25	32	
	dSARM ARM-TIR_2	25	15	
	dSARM ARM-TIR_3	13	16	
	dSARM ARM-TIR_4	28	48	
	dSARM ARM-TIR_5	62	79	

Volume

L	R	T
504.112	214.102	
494.288	544.41	
357.761	220.829	
403.195	353.758	
1138.44	738.658	
1167.64	3482.45	
1139.13	1313.41	
3481.21	3587.29	
958.005	944.4	
573.791	1333.97	
1314.88	722.106	
163.403	476.879	
621.969	618.465	
508.002	455.84	
2647.38		
412.632	432	
299.946	291.956	
262.371	300.91	
522.407	7.12E+02	
883.968	966.387	

Brain lobe volume

L	R	T
1.20E+05	1.29E+05	
1.07E+05	1.51E+05	
8.18E+04	7.18E+04	
1.00E+05	1.05E+05	
3.45E+05	3.76E+05	
6.85E+05	6.06E+05	
3.68E+05	3.80E+05	
6.43E+05	6.58E+05	
2.63E+05	2.97E+05	
2.24E+05	2.57E+05	
3.39E+05	3.30E+05	
9.52E+04	1.04E+05	
1.58E+05	1.60E+05	
1.60E+05	1.36E+05	
3.60E+05		
1.90E+05	1.96E+05	
1.49E+05	1.47E+05	
1.33E+05	1.18E+05	
1.82E+05	1.91E+05	
3.62E+05	3.29E+05	

Normalized Count			Normalized Volume	
L	R	T	L	R
0.0002579	0.00010878		0.00419394	0.00166357
0.00038318	0.00023841		0.00461951	0.00360536
0.00037893	0.00027845		0.00437307	0.0030745
0.00019	0.00024762		0.00403195	0.00336912
0.00019441	0.00012773		0.0033033	0.00196554
9.9253E-05	0.00030673		0.00170428	0.00574279
0.0001902	0.00020804		0.00309513	0.00345872
0.00031237	0.00031763		0.00541016	0.00545181
0.00022466	0.0002289		0.00364794	0.00317906
0.00012031	0.00033522		0.00255686	0.00519965
0.00021239	0.00011818		0.0038787	0.0021882
0.00012606	0.00027911		0.0017166	0.00458979
0.00028481	0.0002375		0.00393651	0.00386541
0.000125	0.00012509		0.00317501	0.00335423
0.00046389			0.00735383	
0.00013166	0.00016353		0.00217314	0.00220772
0.00016725	0.00010218		0.00200669	0.0019888
9.7451E-05	0.0001359		0.0019668	0.00255593
0.00015393	0.00025131		0.00287195	0.0037267
0.00017113	0.0002399		0.00243988	0.00293469

T

Date		Punta Count		Brain lobe vo
12/18/21		L	R	L
	dSARM ARM-TIR_1	39	59	1.63E+05
	dSARM ARM-TIR_2	185	110	1.15E+06
	dSARM ARM-TIR_3	25	59	3.47E+05
	dSARM ARM-TIR_4	46	44	3.06E+05
	dSARM ARM-TIR_5	31	23	2.32E+05
	dSARM ARM-TIR_6	27	58	5.00E+05
	dSARM ARM-TIR_7	20	28	1.63E+05
	dSARM Rescue_1	44	55	1.83E+05
	dSARM Rescue_2	48	58	2.90E+05
	dSARM Rescue_3	39	11	1.99E+05
	dSARM Rescue_4	47	55	2.64E+05
	dSARM Rescue_5	28	33	2.33E+05
	dSARM Rescue_6	59	61	2.89E+05
	OR_1	139	196	1.16E+06
	OR_2	66	96	4.51E+05
	OR_3	82	20	2.90E+05
	OR_4	68	35	4.55E+05
	OR_5	117	208	1.27E+06
	OR_6	57	79	4.06E+05
	OR_7	98	72	5.24E+05

olume		Normalized Count	
R		L	R
2.09E+05		0.00023854	0.00028217
6.12E+05		0.00016099	0.00017961
2.91E+05		7.2094E-05	0.00020265
3.04E+05		0.00015011	0.00014461
1.36E+05		0.00013346	0.00016881
5.66E+05		0.000054	0.00010254
1.55E+05		0.00012305	0.00018037
1.82E+05		0.00024034	0.00030279
2.49E+05		0.00016558	0.00023333
1.71E+05		0.00019606	6.4492E-05
2.73E+05		0.00017814	0.00020144
2.14E+05		0.00012002	0.00015454
3.07E+05		0.00020431	0.00019857
1.24E+06		0.00011995	0.00015819
4.10E+05		0.00014649	0.00023393
3.06E+05		0.00028287	6.5373E-05
4.32E+05		0.0001494	8.0972E-05
1.37E+06		9.1772E-05	0.00015234
4.34E+05		0.00014042	0.00018193
5.35E+05		0.00018694	0.00013461

Date		Punta Count		Brain lobe vo
12/19/21		L	R	L
	dSARM Rescue_1	7	4	6.01E+04
	dSARM Rescue_2	6	9	4.63E+04
	dSARM Rescue_3	6	7	6.84E+04
	dSARM Rescue_4	13	26	5.70E+04
	dSARM Rescue_5	18	19	7.22E+04
	dSARM Rescue_6	25	21	4.92E+04
	dSARM TIR_1	20	43	4.17E+04
	dSARM TIR_2	42	36	6.48E+04
	dSARM TIR_3	62	51	1.32E+05
	dSARM TIR_4	48	39	8.00E+04
	dSARM TIR_5	19	30	5.93E+04
	dSARM TIR_6	27	39	7.16E+04
	dSARM TIR_7	48	53	1.00E+05

lume		Normalized Count	
R		L	R
8.30E+04		0.0001165	4.8193E-05
4.97E+04		0.00012964	0.00018099
5.26E+04		8.7674E-05	0.00013298
5.88E+04		0.00022807	0.00044255
5.34E+04		0.00024929	0.0003559
4.91E+04		0.00050762	0.00042736
4.47E+04		0.00047962	0.0009624
5.81E+04		0.00064823	0.00061984
7.79E+04		0.0004697	0.00065477
4.98E+04		0.00060008	0.00078359
7.69E+04		0.00032057	0.00039007
1.02E+05		0.00037709	0.00038235
9.70E+04		0.00048	0.00054639

Date		Punta Count		Brain lobe volume	
12/20/21		L	R	L	R
	dSARM Rescue_1	44	23	1.04E+05	6.29E+04
	dSARM Rescue_2	14	11	4.72E+04	5.14E+04
	dSARM Rescue_3	25	32	1.21E+05	5.92E+04
	dSARM Rescue_4	16	8	5.87E+04	7.54E+04
	dSARM Rescue_5	9	24	4.60E+04	6.67E+04
	dSARM Rescue_6	16	15	5.26E+04	7.36E+04
	dSARM Rescue_7	20	11	5.33E+04	3.18E+04
	dSARM TIR_1	29	34	5.54E+04	6.17E+04
	dSARM TIR_2	36	41	8.45E+04	5.15E+04
	dSARM TIR_3	40	37	5.54E+04	5.26E+04
	dSARM TIR_4	44	40	7.39E+04	6.45E+04
	dSARM TIR_5	41	24	1.29E+05	5.88E+04
	dSARM TIR_6	37	27	7.11E+04	4.14E+04
	dSARM TIR_7	11	43	3.25E+04	2.87E+04
	dSARM TIR_8	27	24	6.56E+04	3.93E+04
	dSARM TIR_9	22	45	7.48E+04	5.63E+04
	dSARM TIR_10	36	38	8.84E+04	5.84E+04

Normalized Count

L	R
0.00042478	0.00036552
0.00029688	0.00021382
0.00020609	0.00054015
0.00027266	0.00010617
0.00019559	0.00035995
0.00030423	0.00020372
0.00037553	0.00034547
0.00052358	0.00047002
0.00042604	0.00069878
0.00072149	0.00075981
0.00059508	0.00068169
0.00031882	0.00069769
0.00052069	0.000893
0.00033795	0.00038388
0.00041132	0.00068778
0.00029413	0.00039108
0.00040728	0.00061626
